# Unlocking the Gates: A Novel Diagnostic Molecule for Quantifying Efflux Levels in Gram‐Positive Bacteria

**DOI:** 10.1002/adhm.202404145

**Published:** 2025-03-11

**Authors:** Mrunal Patil, Tatiana Munteanu, Gaël Brasseur, Carolina Ferreira, Sofia Santos Costa, Isabel Couto, Mohd Athar, Elisa Asunis, Attilio Vittorio Vargiu, Miguel Viveiros, Carole DiGiorgio, Frédéric Brunel, Jean‐Manuel Raimundo, Michel Camplo, Olivier Siri, Jean‐Michel Bolla

**Affiliations:** ^1^ Aix Marseille Université CNRS CINaM UMR 7325 Campus de Luminy Case 913, Cedex 09 Marseille 13288 France; ^2^ Aix Marseille Université INSERM SSA MCT Marseille 13385 France; ^3^ Laboratoire de Chimie Bactérienne Institut de Microbiologie de la Méditerranée CNRS Aix‐Marseille Université Marseille 13402 France; ^4^ Global Health and Tropical Medicine GHTM Associate Laboratory in Translation and Innovation Towards Global Health LA‐REAL Instituto de Higiene e Medicina Tropical IHMT Universidade NOVA de Lisboa UNL Lisbon 1349‐008 Portugal; ^5^ Physics Department University of Cagliari SP 8, km 0.700 Monserrato 09042 Cagliari Italy; ^6^ Laboratoire de Mutagénèse Environnementale Aix‐Marseille Université CNRS IRD Avignon Université IMBE UMR 7263 Marseille 13385 France

**Keywords:** chemical synthesis, clinical strains, diagnostic, efflux pump, fluorescence

## Abstract

Efflux‐mediated antibiotic resistance poses a significant global threat, affecting diverse bacterial species. Clinicians recognize the danger of efflux mechanisms during antibiotic treatment, yet precise diagnostic tools remain unavailable. The antibiogram currently infers abnormal efflux pump activity in clinical isolates, which is subsequently confirmed through transcriptomic or genomic analysis. This study harnesses the colorimetric, fluorescence, and solubility properties of phenazinium derivatives to develop a rapid protocol for detecting bacterial efflux. Among several synthesized phenazinium compounds, the compound demonstrating differential MIC in *Staphylococcus* efflux mutants and exhibiting appropriate physicochemical properties is selected. A diagnostic protocol is developed using the selected compound to assess efflux levels, categorized as no, weak, or strong, through colorimetry and spectroscopy techniques. Testing on Gram‐positive efflux mutants and clinical *Staphylococcus* isolates further validates the approach. In‐silico docking analysis confirms the interaction between the chosen compound and the NorA efflux pump in *S. aureus*. Flow cytometry is employed to re‐evaluate the detection assays. The developed molecule and protocol represent the first known method to evaluate efflux levels in any Gram‐positive species through a streamlined and user‐friendly process. This pioneering test significantly advances the epidemiological analysis of efflux mechanisms and enables more informed therapeutic decision‐making, leading to more effective treatment.

## Graphical abstract

Efflux‐mediated resistance compromises antibiotic efficacy, yet rapid detection remains elusive. This study presents a novel phenazinium‐based diagnostic molecule for quantifying bacterial efflux levels in Gram‐positive pathogens. The optimized compound enables precise, real‐time efflux assessment using fluorescence and colorimetric techniques, facilitating clinical decision‐making and antimicrobial stewardship. Molecular docking and flow cytometry further validate its diagnostic potential.

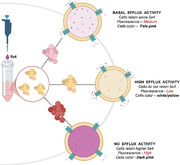

## 1 Introduction

The long‐standing, silent epidemic known as antimicrobial resistance (AMR) has grown to be a serious public health emergency. Selective pressures brought on by overuse or improper use of antibiotics have accelerated the rise of AMR bacteria.^[^ ^1^ ^]^ AMR and its substantial social and economic repercussions have been the subject of serious deliberations at the World Health Organization (WHO), G7, and G20 summits.^[^ ^2^ ^]^ Despite significant breakthroughs in medical technology, detecting and characterizing infections can take several days. As a result, practitioners are required to start empiric antibiotic treatment, which is typically broad‐spectrum. This may not only increase the risk of over‐drug exposure (i.e., microbiome dysbiosis) but also aggravate the ongoing AMR crisis.^[^ ^3^ ^]^ Controlling overuse of antimicrobials requires regular “review and revise” of patients with the goal of de‐escalation (i.e., switching to “narrower spectrum” agents and/or reducing the number of drugs prescribed, etc.). Therefore, providing rapid and reliable diagnostic results can assist in “review and revise” and is a significant way for microbiology laboratories to contribute to antimicrobial stewardship.^[^ ^4^ ^]^ The PHG Foundation and Health Action International (HAI) most recently declared in 2023 that combating antibiotic resistance can greatly benefit from the development and broad application of more precise and quick diagnostic tests.^[^ ^5^ ^]^ In 2019, the Organization for Economic Cooperation and Development (OECD) issued a similar statement.

Although, antibacterial resistance is developed through various mechanisms, increased active efflux of drugs is of particular importance, especially as a single type of multidrug (MDR) efflux pump can induce resistance to multiple drugs simultaneously, resulting in an MDR phenotype. Bacterial efflux pumps contribute to antibiotic resistance and play an important role in fundamental bacterial physiology (regulation of pH, transport of toxins, etc.) and pathogenicity. Efflux pumps belong to two main classes: i) primary transporters, also known as ATP‐binding cassette (ABC) transporters, which obtain their energy from ATP, and ii) secondary transporters, which obtain their energy from the gradient of protons or sodium. Secondary transporters are further classified into four families: the major facilitator superfamily (MFS), the resistance‐nodulation and cell division (RND) superfamily, the small multidrug resistance (SMR) family, and the multidrug and toxic compound extrusion (MATE) family. These families are based on sequence conservation and functional similarities.^[^ ^6^ ^]^ These efflux proteins are exquisite bacterial weapons for evading traditional antibiotic therapy and hence should be considered clinically significant in the development of efficient treatment adjuvants such as efflux inhibitors.^[^ ^7^, ^8^ ^]^ Understanding these mechanisms necessitates the ability to rapidly quantify efflux pump activity. Unfortunately, clinicians often overlook efflux‐mediated resistance due to the lack of rapid efflux detection kits. Conventionally, Antimicrobial Susceptibility Technologies (AST) have been used by clinicians; However, this methodology fails to provide an assessment of resistance due to efflux mechanisms, and the regulatory requirements for AST vary depending on the country or region.^[^ ^9^ ^]^ Many literatures describe a variety of methods for assessing efflux in bacteria. These techniques fall into two groups: i) those that quantify efflux directly, or the amount of a substrate that is pumped out; and ii) those that quantify the amount of a substrate molecule that accumulates inside the cell, the measurement of which is used to indirectly deduce the efflux activity.^[^ ^10^ ^]^ Both methods make use of fluorescent probes like Hoechst‐33342, Ethidium bromide (EtBr), or fluorogenic substances like Fluorescein‐di‐β‐D‐galactopyranoside, as well as lipophilic dyes like Nile Red. The most commonly used dyes are EtBr/Hoechst 33342, which fluoresce when bonded to DNA. However, this DNA‐intercalating nature of the dye causes its efflux to be delayed since it requires more dissociation steps and most likely more than one efflux event to quantify the efflux amount appropriately.^[^ ^11^ ^]^ A solid agar method‐based EtBr cart‐wheel method (EtBrCW) was designed, however, it requires overnight incubation of bacteria to visualize the result and does not account for compounds’ diffusion. Furthermore, EtBr is mutagenic, making its management challenging. Lipophilic dyes, such as Nile Red, are known to adsorb on plasticware^[^ ^12^ ^]^ and have been less successful in measuring efflux in *Pseudomonas* spp.^[^ ^10^ ^]^ Alternatively, fluoroquinolones (such as ciprofloxacin and norfloxacin) have been used to detect efflux. The accumulation within the cells is accessed by utilizing their inherent fluorescence.^[^ ^13^ ^]^ Although this method is more clinically relevant than some dyes, its procedure is time‐consuming and involves several steps such as loading the drug followed by cell lysis and comparing the fluorescence to its supernatant concerning its dry cell weight. Another time‐consuming and laborious method involves preloading the dyes in energy‐deficient media and then triggering efflux by the addition of glucose.^[^ ^14^ ^]^ Most recently, matrix‐assisted laser desorption ionization‐time of flight mass spectrometry (MALDI‐TOF MS) has been used to monitor drug efflux by a multidrug transporter.^[^ ^15^ ^]^ However, this technique is not easily accessible and is expensive. In conclusion, many assays used today are time‐consuming and require a range of instruments or reagents, which may not be available to clinicians, particularly in war zones or low‐income countries.

In the pursuit of advancing bacterial efflux detection methodologies, the authors of this paper have synthesized and developed a novel phenazinium compound specifically designed for this purpose. At present, no single dye offers both quantitative and qualitative (visual) detection of efflux. Our phenazinium compound (Granted patent FR3132099A1) marks a significant advancement by enabling precise quantitative differential staining of cell pellets to assess bacterial efflux activity. This innovative approach allows for the rapid identification of efflux‐mediated multidrug‐resistant (MDR) phenotypes within minutes, eliminating the need for complex instruments or reagents. While the current scope of our study is limited to Gram‐positive bacteria, we intend to include additional species in future research. Our optimized phenazinium compound has been licensed and pre‐marketed under the name “ColorFlux” by Idylle Labs in France, marking a significant step forward in antimicrobial resistance research and diagnostics.

## 2 Results and Discussion

### 2.1 Synthesis

**Scheme 1:**
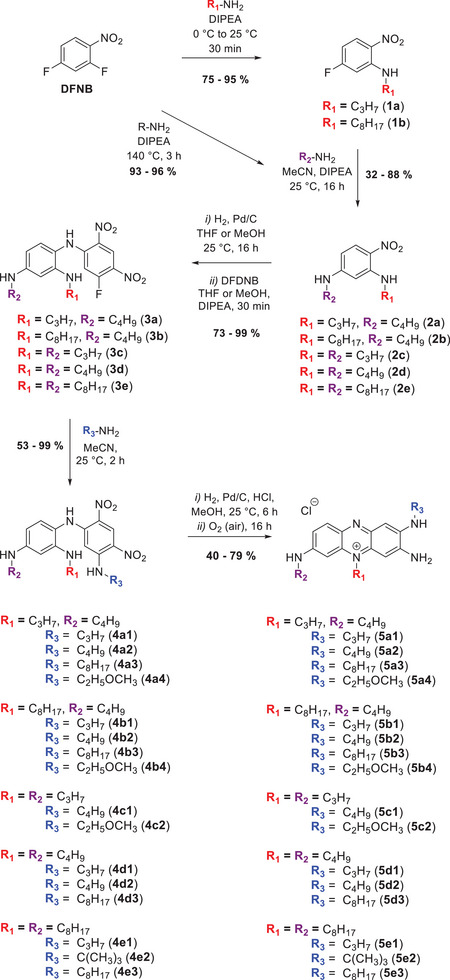
Synthesis strategy towards triamino‐phenazinium dyes.

The synthesis strategy for the triamino‐phenazinium dyes followed a reported protocol^[^ ^16^ ^]^ that we extended to produce a library of dyes bearing identical or distinct alkyl substituents R_1_‐R_2_‐R_3_ (**Scheme** **1**). We started with a simple one or two‐step nucleophilic substitution of the commercially available 2,4‐difluoro‐1‐nitrobenzene with an excess of the corresponding amine, which afforded the 2,4‐diamino‐1‐nitrobenzene derivatives **2c‐e** (one step) and **2a‐b** (two‐step) in excellent yields. The reduction of the nitro function was carried out using the published procedure, leading to the intermediates **3a‐e**, being isolated as brown solids. For the compounds **3a‐b** replacing THF with MeOH increased the yield from 77% to 81% and from 53% to 99% respectively. The remaining fluorine moiety was further reacted with an excess of the amine at room temperature. This way the phenazinium precursors **4a‐e** were isolated in good to quantitative yields. The derivatives **4a‐e** were finally subjected to reduction according to the protocol mentioned before, affording the desired triamino‐phenazinium fluorophores **5a‐e** in yields spanning from 40% to 79%.

### 2.2 Fluorescence Intensity Estimation

**Figure 1:**
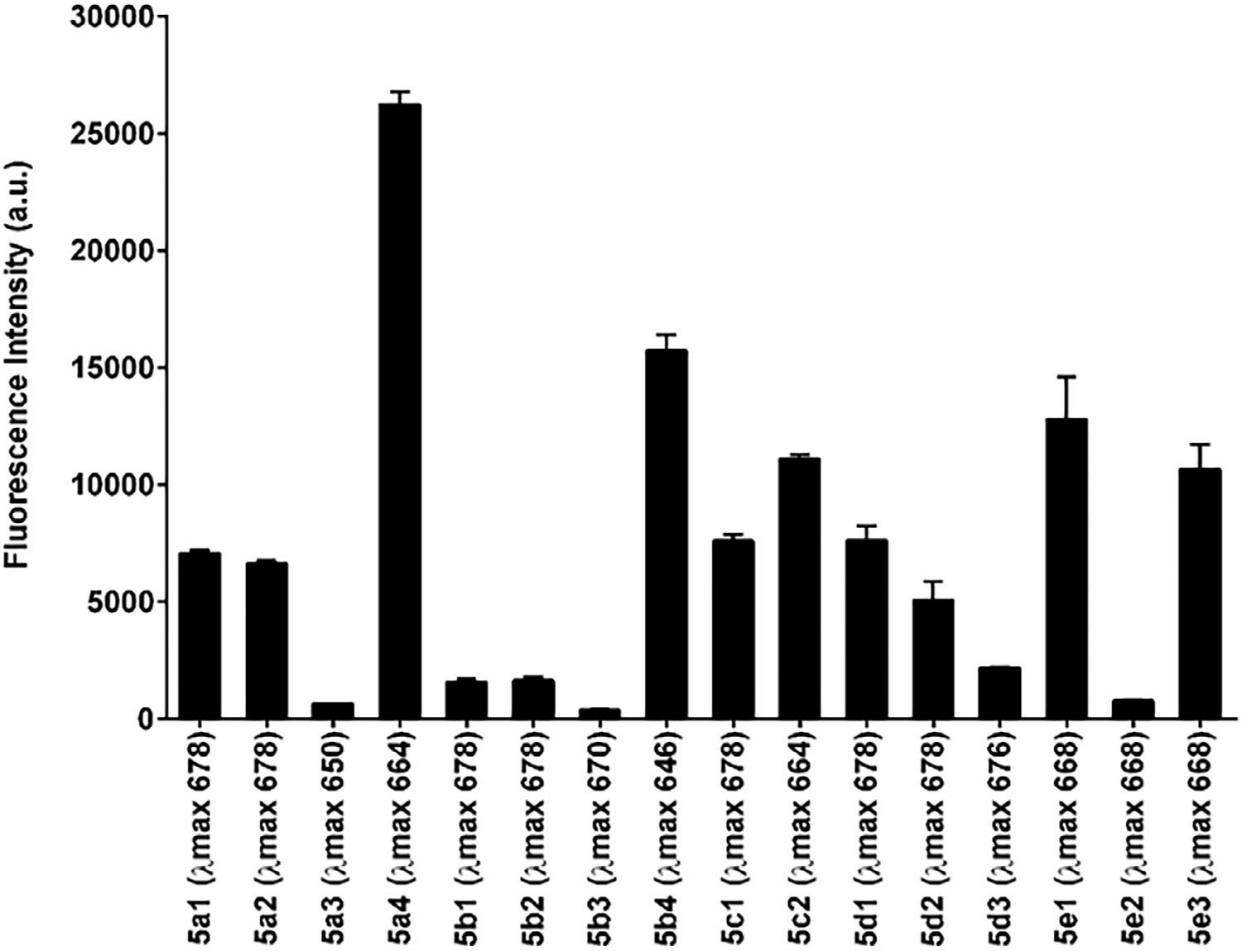
The average fluorescence intensity of the phenazinium derivative was measured using a fluorescence reader set to an excitation wavelength of 530 nm, with emission scanned between 560 and 800 nm. The values in parentheses on the x‐axis indicate the wavelengths at which the maximum emission intensity was observed.

Fluorogenic molecules are essential for detecting a wide range of dynamic intracellular activities. To develop a fluorogenic probe with high sensitivity, it is important to ensure it has sufficient fluorescence properties.^[^ ^17^ ^]^ We anticipate that our diagnostic molecule to show more fluorescence intensity to facilitate its detection, particularly in our experiment where the goal is to quantify residual fluorescence in the cell after efflux. Considering the said, we estimated fluorescent intensities of each phenazinium derivative at a fixed concentration (1 µg mL^−1^). Compound **5a4** had the maximum fluorescence intensity, followed by **5b4, 5e1** and **5e3** (**Figure** **1**). Both **5a4** and **5b4** compounds contained a methoxy group at the R3 position and a butyl group at the R2 position. We believe that the presence of methoxy at the R3 position might have contributed to the better water solubility of these compounds thus increasing their fluorescent intensity. Interestingly, compounds **5e1** and **5e3** also showed significant fluorescence, but this fluorescence appeared to be restricted because of the presence of octyl chains at positions R1 and R2, which also reduced their solubility.

### 2.3 Solution State Stability and Minimal Inhibitory Concentration (MIC)

The final test diagnostic protocol will be designed in MH‐II broth; therefore, it is crucial to ensure that the molecules remain stable in this medium. Interestingly, all the compounds (**5a4**, **5b4**, and **5c2**) with a methoxy group at the R3 position showed excellent solubility and stability in the test media. In contrast, molecules containing a hydrophobic group (the C8 chain) on the R3 site exhibited precipitation in MH‐II. Thus, the presence of a methoxy group at the R3 position is responsible for the compound's solubility and stability, but the presence of a higher chain makes the molecule hydrophobic, reducing its solubility in water. Furthermore, all molecules with C8 on the R1 or R2 position except **5b4** and **5e2** precipitated in the MH‐II medium. Despite the C8 chain on the R1 position, the methoxy on the R3 position may have accounted for **5b4**’s solution state solubility. However, the solution state stability of **5e2** could not be explained. Solution state stability also affects the MIC values. **Table** **1** shows that compounds **5d3**,**5b2** and **5b1** had higher MIC values and that they precipitated at concentrations above 64 µg mL^−1^, making it difficult to anticipate their MIC values. The objective of investigating MIC values on NorA efflux mutants was to determine whether any phenazinium derivatives were the substrates of these pumps. We investigated the NorA efflux mutant because it is well‐characterized and a reliable model for researching efflux in Gram‐positive bacteria. A detailed description of NorA efflux mutants and other mutants used in further studies are enlisted and described in **Table** **2**. The drug Norfloxacin (NOR) was used as a control for this experiment as it is an excellent substrate of NorA.^[^ ^18^ ^]^ Although several methods for evaluating efflux pump activity in bacterial cells are described in the literature, ethidium bromide (EtBr), an efflux pump substrate, has been widely used.^[^ ^19^, ^20^ ^]^ EtBr is a strong efflux substrate, but it still requires a proton motive force uncoupler to remain in the cell. Some alternative ideas were generated using the EtBrCW method, but this delayed the efflux measurement.^[^ ^21^ ^]^ Given the innate property of phenazinium derivatives' color and fluorescence, we investigated their efflux substrate selectivity in the *norA* efflux mutant. As observed from Table 1, we concluded that compound **5a4** is a good substrate for the NorA efflux pump, as it exhibited a fourfold or greater difference between the NorA wild‐type, overexpressing, and deleted mutants. Furthermore, the solution‐state stability of **5a4** makes it an ideal candidate for developing a diagnostic molecule for efflux testing.

**Table 1:** MIC values of phenazinium derivatives against *Staphylococcus aureus* reference strain and *norA* mutants.

MIC values expressed in µg mL^−1^					Solution state stability
Test molecules	ATCC 25923 (Reference, basal)	SA 1199 (Wild‐type, basal)	SA 1199‐B (*norA* overexpressing)	SA‐K1758 (∆*norA*)	
**5a1**	1	1	2	0.5	Visible precipitation was observed at 128 µg mL^−1^ and beyond
**5a2**	1	1	2	0.25	Visible precipitation was observed at 512 µg mL^−1^ and beyond
**5a3**	64	64	64	8	Visible precipitation was observed at 128 µg mL^−1^ and beyond
**5a4**	2	2	8‐16	0.5‐1	Stable
**5b1** ^b)^	NA	NA	NA	NA	Visible precipitation was observed at 64 µg mL^−1^ and beyond
**5b2** ^b)^	NA	NA	NA	NA	Visible precipitation was observed at 64 µg mL^−1^ and beyond
**5b3**	32	32	32‐64	32	Visible precipitation was observed at 128 µg mL^−1^ and beyond
**5b4**	1	1	1	2	Stable
**5c1**	2	2	2	0.5	Stable
**5c2**	4	8	8	4	Stable
**5d1**	16	16	16	8	Visible precipitation was observed at 128 µg mL^−1^ and beyond
**5d2**	1	1	0.5–1	0.5	Visible precipitation was observed at 512 µg mL^−1^ and beyond.
**5d3** ^b)^	NA	NA	NA	NA	Visible precipitation was observed at 64 µg mL^−1^ and beyond
**5e1**	16	16	16	8	Visible precipitation was observed at 128 µg mL^−1^
**5e2**	8	8	8	8	Stable
**5e3**	32	64	64	64	Visible precipitation was observed at 128 µg mL^−1^
**NOR** ^a)^	2	2	32	0.25	Control

^a)^ NOR; norfloxacin;

^b)^ Some derivatives’ MIC values could not be determined since their MIC values were higher than their precipitation concentrations. The MIC values of such derivative are provided as NA (not applicable).

**Table 2:** Strains and efflux mutants were used in this study.

Strain	Source	Relevant characteristics	Ref.
*Staphylococcus aureus*			
ATCC 25923	American‐type culture collection	Reference strain	[22, 23]
SA‐1199	Endocarditis patient	Clinical isolate, Methicillin susceptible	
SA‐1199B	Derivative of SA1199	*norA*‐overproducing derivative of SA‐1199	[19]
SA‐K1758	Derivative of NCTC‐8325‐4	Knocked out NorA (Δ*norA)*	
SA‐K1712	Derivative of NCTC‐8325‐4	NCTC 8325‐4 with *norA* disrupted by *tet*(K)	[24]
SA‐K1748	Derivative of SA‐K1712	NorA multidrug efflux transporter, MDR mutant of SA‐K1712, *mepA*‐overexpressing strain	
SA‐K2068	Derivative of NCTC‐8325‐4	*mepA‐*overexpressing strain, MDR mutant of NCTC 8325‐4, Non‐NorA multidrug (organic cations, moxifloxacin)	
*Bacillus subtilis*			
DSM 402	German Collection of Microorganisms and Cell Cultures GmbH	Reference strain	[25]
BmrA +++	Derivative of DSM 402	strain with overexpression of the *bmrA* gene	
ΔBmrA	Derivative of DSM 402	strain with deletion of *bmrA* gene	
*Streptococcus pneumoniae*			
R6 (ATCC BAA‐255)	American‐type culture collection	Wild type, Fluoroquinolone susceptible laboratory strain	[26]
H4	R6 derivative issue from transformation of a *S. pneumoniae* DNA of a clinical isolate with over‐expression of patA/patB into R6	Strain with increased *patA/patB* expression	[26]
ΔPat A/B	R6 derivative with *patA/ patB* knocked out	Strain with no *patA/patB* expression	[27]

### 2.4 Assessing the Diagnostic Efficiency of Compound 5a4 Against Gram‐Positive Bacterial Efflux Mutants

#### 2.4.1 Detection of Efflux Mediated Resistance in *Staphylococcus aureus*

**Figure 2:**
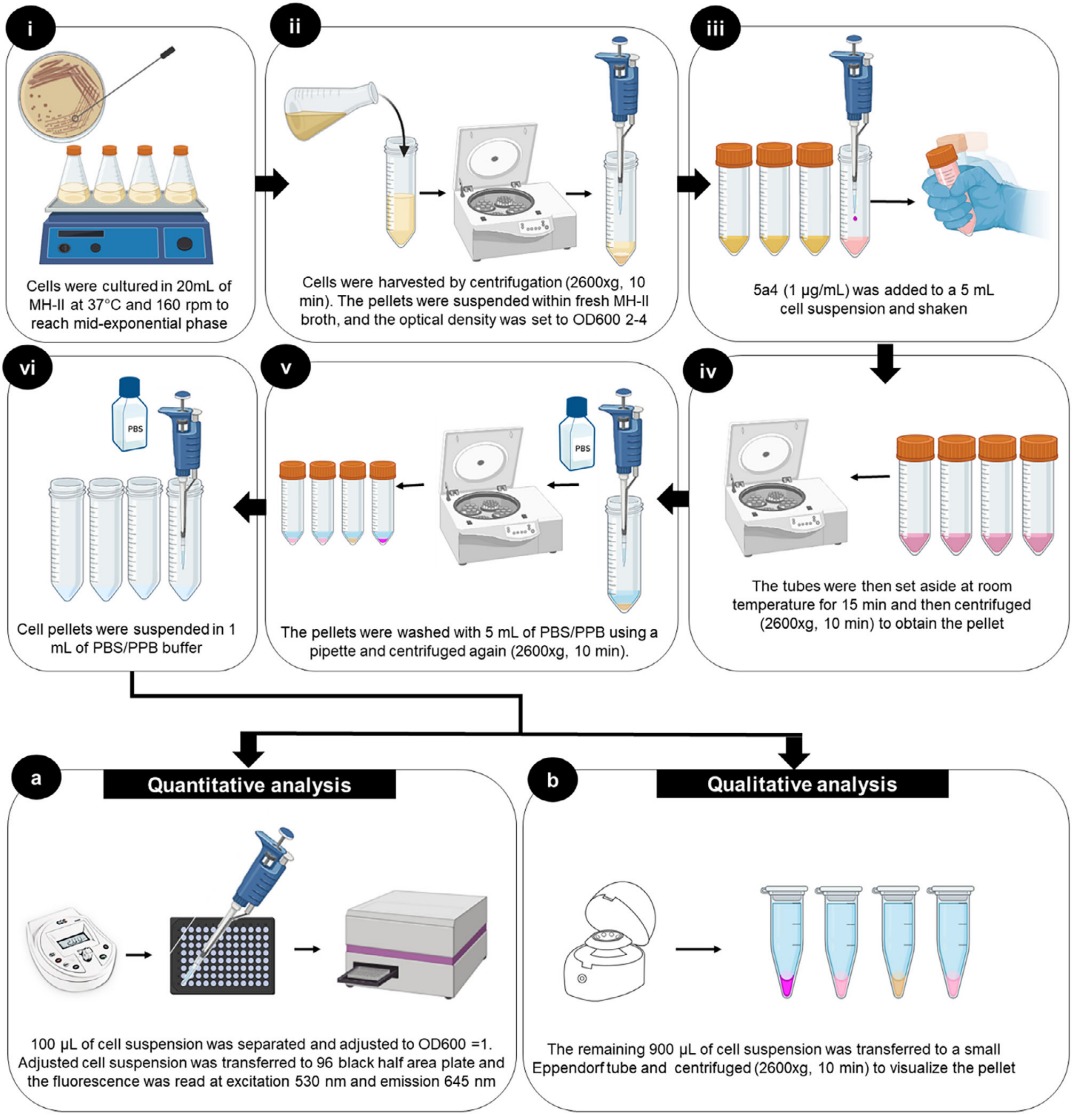
Illustration of the protocol utilizing compound 5a4 to investigate the differential efflux activity of various Gram‐positive bacteria.

The approach for our diagnostic molecule **5a4** was initially devised by using *S. aureus* NorA efflux mutants. We were able to fine‐tune the procedure in MH‐II broth after several attempts (not discussed in the current article). The methodology for using **5a4** for diagnostic purposes is illustrated in **Figure** **2**. This protocol was subsequently validated on various other pumps in Gram‐positive bacteria.

**Figure 3:**
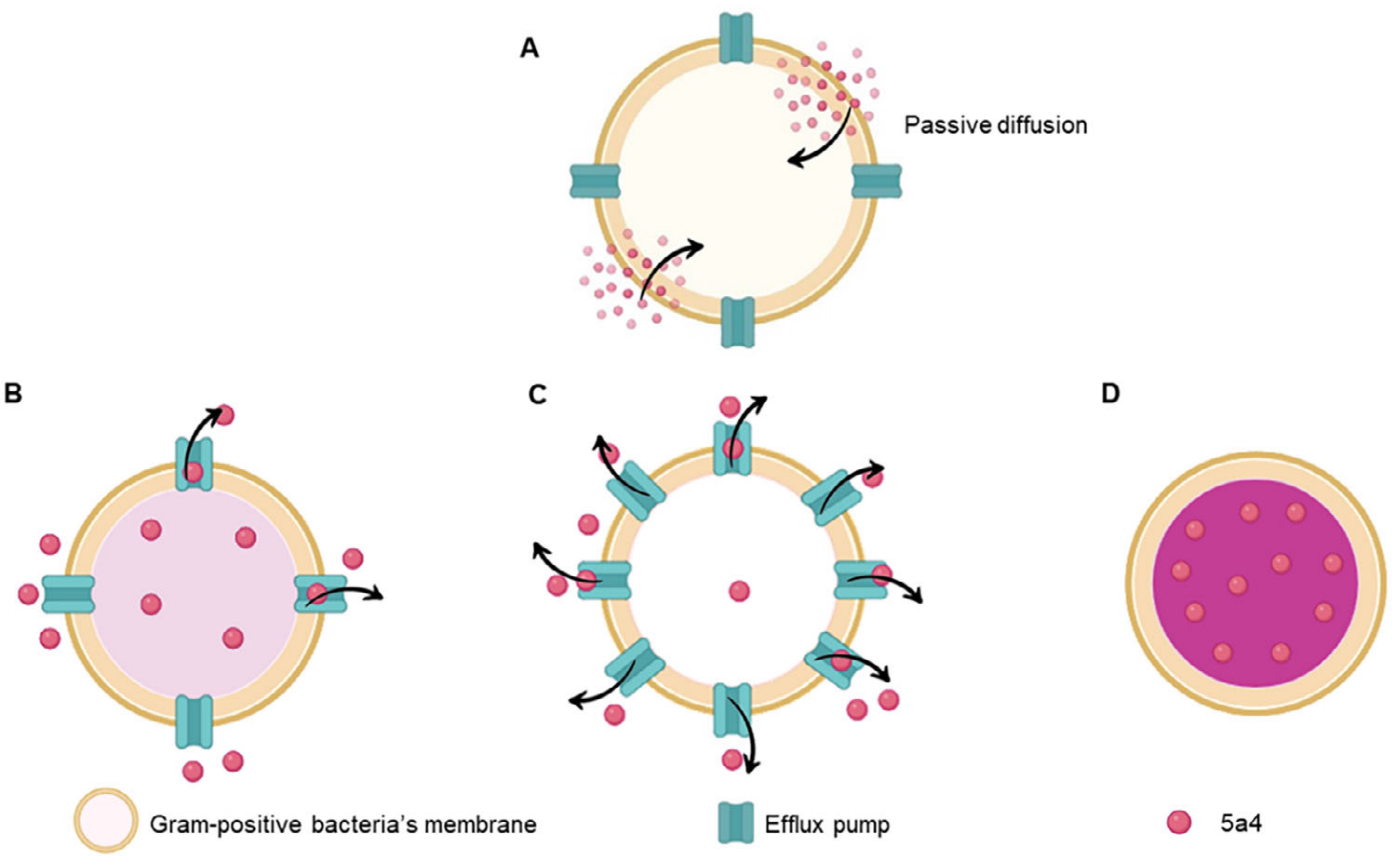
Schematic representation of the process by which compound 5a4 enters cells and stains them based on their efflux capacities: A) Passive transport of 5a4 into the cell; B) Bacterial cells with basal level of efflux gene expression pump out sufficient 5a4 to maintain internal homeostasis. Consequently, a moderate concentration of 5a4 accumulates in the cytoplasm, resulting in a pale pink hue. C) Bacterial cells overexpressing the efflux pump gene level expel nearly all 5a4, leading to a very low intracellular concentration. As a result, these cells either stain minimally or retain their default color (pale yellow or white depending on the microbe used). D) Bacterial cells with the efflux pump gene knocked out, cannot efficiently expel 5a4, leading to a high intracellular concentration and a dark pink coloration.

The core findings of the **5a4** protocol were based on the premise of estimating residual intracellular **5a4** concentrations based on the efflux capacities of cells (**Figure** **3**). It was seen that cells with normal efflux capacity would preserve the equilibrium between their internal and exterior environments, allowing them to retain some color thereby acquiring a light pink color (Figure 3B). Strong efflux capabilities, or overexpression of the efflux pump gene, would allow cells to pump out all the **5a4**, leaving the cells with little to no **5a4** concentration (Figure 3C). These cells would acquire very little stain or none at all, retaining the default color of the bacteria. Cells with efflux gene inactivated efflux pump gene inactivated or cells unable to effectively pump out **5a4**, leading them to stain dark pink (Figure 3D). The stain acquired by the efflux pump gene inactivated strain also confirms that **5a4** is capable of passively entering the cell and that its transit outside the cytoplasm is mainly dependent on the efflux pump (Figure 3A).

**Figure 4:**
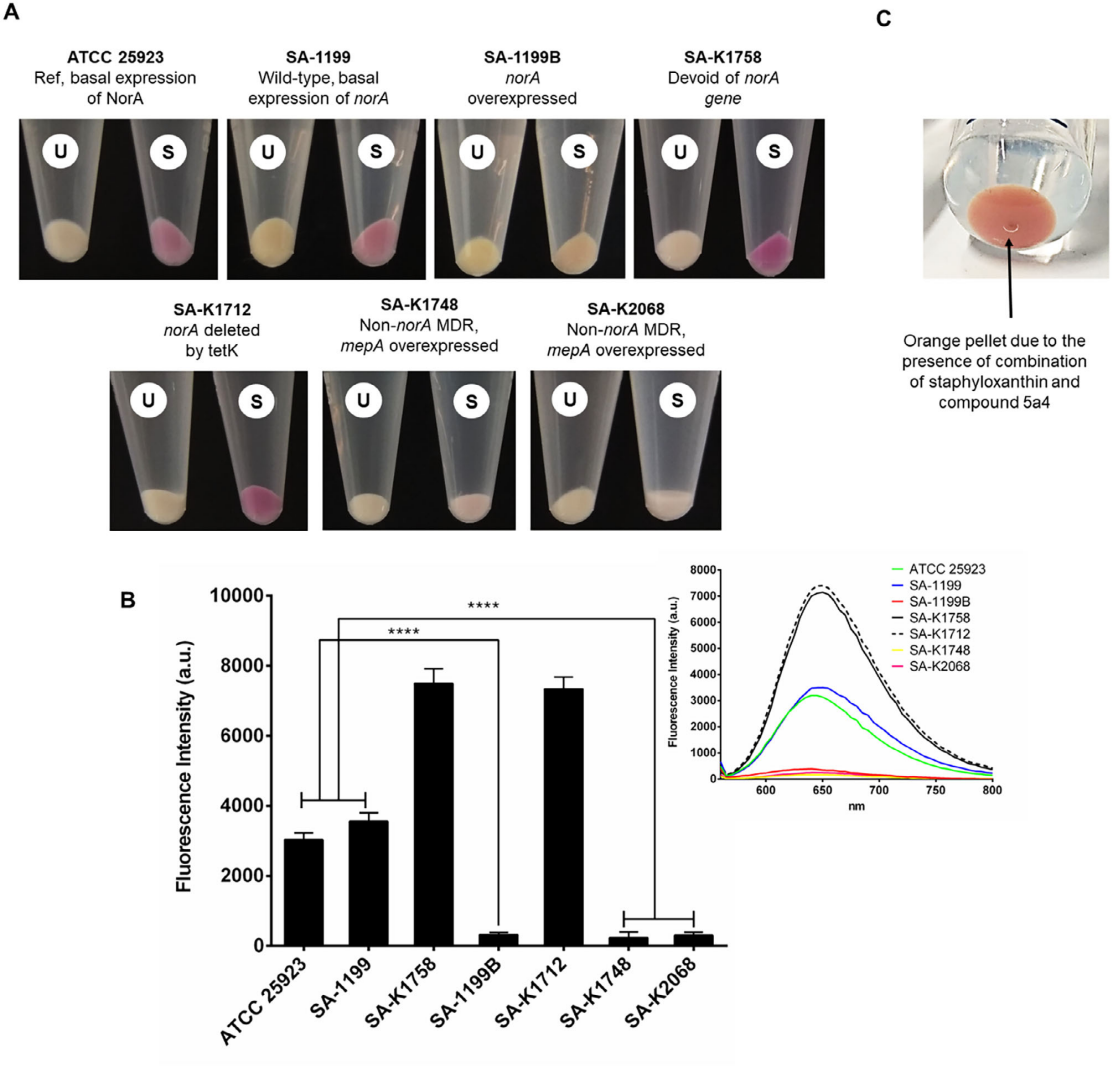
A) Visible distinct colored cell pellets of *S. aureus* dependent on their efflux capacity. The cell pellet of unstained (U; not treated with 5a4 protocol) can be compared to the bacterial pellet of stained cells (S; treated with 5a4 protocol) offering qualitative analysis. B) Bar graph representing the average fluorescence intensities of 5a4 treated pellets obtained by a fluorescence spectrometer offering qualitative analysis. The inset picture depicts the emission spectra of each test strain scanned from 560 to 800 nm at an excitation of 530 nm. C) An example of an occasionally observed orange pellet seen when 5a4 interacts with “staphyloxanthin” produced by *S. aureus* cells. The pellet seen in this figure belongs to the SA‐1199 stain grown in MH‐II until the late log phase.

Since the mentioned protocol was initially tested on *S. aureus norA* efflux mutants, we address its results first. Overexpression of the *norA* gene is linked to enhanced resistance to a wide range of resistance patterns, including to hydrophilic quinolones, dyes, benzalkonium chloride, and tetraphenylphosphonium ions.^[^ ^28^, ^29^ ^]^ It is anticipated that the *norA* gene is overexpressed in ≈40% of *S. aureus* strains, especially in Methicillin‐resistant *Staphylococcus aureus* (MRSA) strains.^[^ ^30^, ^31^ ^]^ **Figure** **4** shows the results of **5a4** and our optimized protocol on *S. aureus* reference strain, wild‐type, *norA* overexpressing, and inactivated mutant. As depicted in Figure 4A, cells subjected to **5a4** developed visibly distinct color cell pellets dependent on their efflux capacity. The cell pellet of the *norA* overexpressing mutant (SA‐1199B) remained unstained depicting a pale white hue due to the complete efflux of **5a4** from the cell. This pellet was distinct from cells with basal *norA* efflux activity (*S. aureus* ATCC 25923 and SA‐1199, stained light pink). Cells devoid of a functional NorA (SA‐K1758) developed a vivid pink stain. The Figure 4B depicted that, the fluorescence intensity of SA‐1199B (*norA* overexpressing strain) was significantly (p<0.0001) low than that of wild‐type and the reference strain bacteria exhibiting basal efflux activity. It was noted that some pellets may occasionally appear orange because of the presence of both **5a4** and “staphyloxanthin,” a carotenoid pigment that gives certain *S. aureus* strains their peculiar golden color. One such example of an orange pellet of SA‐1199 grown until the late log phase is depicted in Figure 4C.

Another set of *S. aureus* efflux mutants tested in the current study focused on the class of the MATE proteins. MATE transporters are comparable to major facilitator superfamily (MFS) concerning their energy sources. They utilize the energy obtained from H+ or Na+ (only MATE) electrochemical gradients to transport antimicrobial agents and organic cations outside the cell, thus reducing their intracellular concentrations.^[^ ^32^ ^]^ MepA belongs to the chromosomally‐encoded MATE family among which MepA is widely studied and well‐characterized.^[^ ^33^ ^]^ The SA‐K1748 and SA‐K2068 (*mepA* overexpressing mutants) pellets showed a pale yellow (default color) appearance as the cells fully pumped out **5a4**, as shown in Figure 4A. Both SA‐K1748 and SA‐K2068 when compared to wild‐type and the reference strains exhibited significantly (*p* < 0.0001) low fluorescence intensity. SA‐K1748 is a mutant derived from a *norA*‐disrupted parent (SA‐K1712), ruling out the role of NorA in its MDR phenotype. The genome‐wide transcriptional profiles conducted by Kaatz et al.,^[^ ^34^ ^]^ revealed that SA‐K2068 has no known topoisomerase mutations and that its MDR phenotype is unrelated to NorA; as a result, it was inferred that **5a4** could also detect efflux activity mediated by MepA.

#### 2.4.2 Detection of Efflux Mediate Resistance in *Bacillus subtilis*

**Figure 5:**
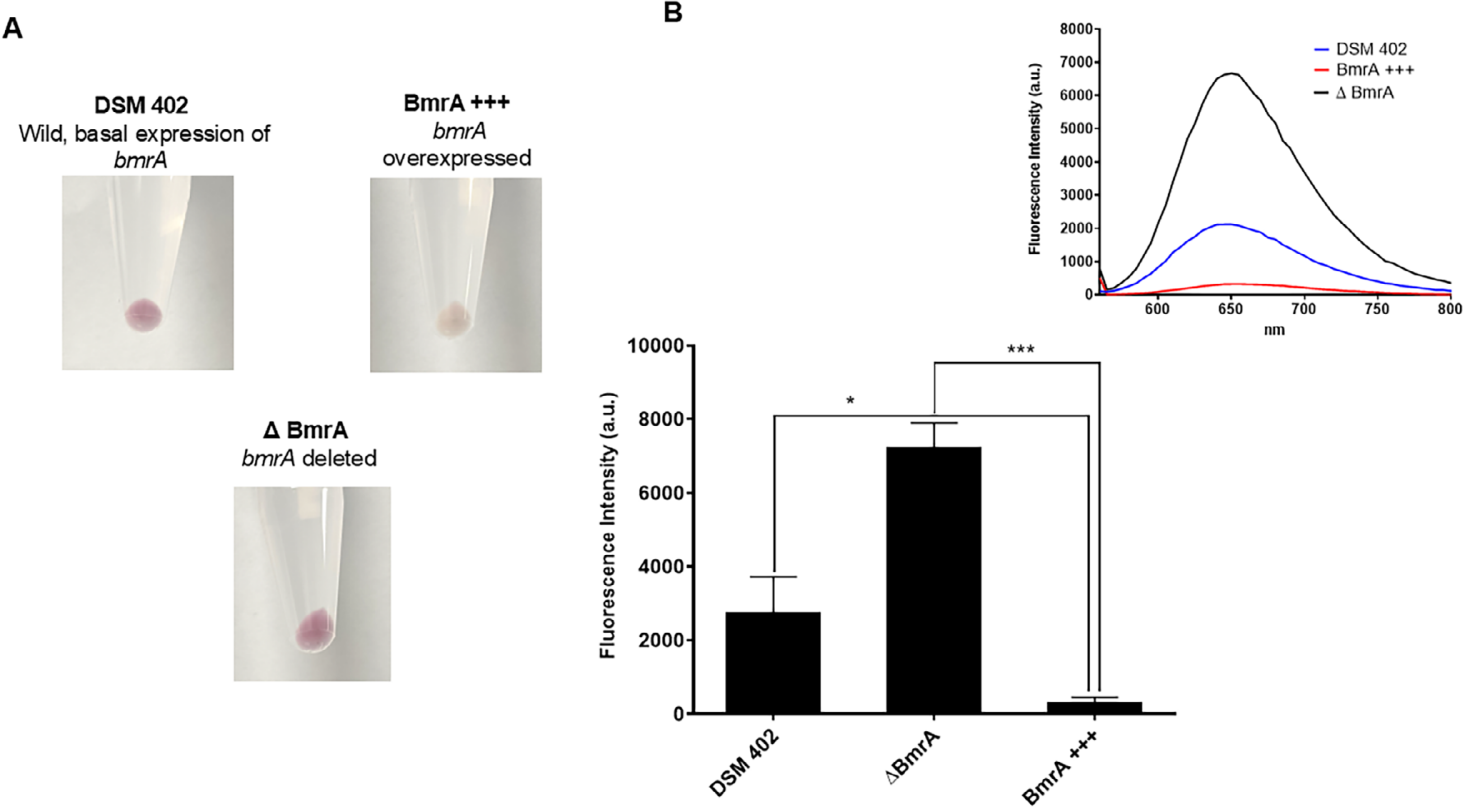
A) Visible distinct colored cell pellets of *B. subtilis* dependent on their efflux capacity after treatment of 5a4. B) Bar graph representing average fluorescence intensities of each pellet obtained by a fluorescence spectrometer. The inset picture depicts the emission spectra of each test strain scanned from 560 to 800 nm at an excitation of 530 nm.

Steinfeld et al.,^[^ ^25^ ^]^ were the first to demonstrate that the ABC transporter is constitutively expressed in a wild‐type strain and can actively pump drugs out of *B. subtilis*. The BmrA and NorA proteins are both structurally and functionally identical. However, the energy for transport in these proteins comes from a catalytic cycle in the nucleotide‐binding domains (NBDs), that involves ATP binding, hydrolysis, and ADP/inorganic phosphate (Pi) release.^[^ ^25^ ^]^ Like the NorA pump, these pumps are typically monitored using an intracellular EtBr accumulation test.^[^ ^35^ ^]^ Given this, we tested **5a4** on BmrA efflux mutants and found outcomes comparable to those described in staphylococcus *norA* mutants (**Figure** **5**). It was found that *bmrA* overexpressed strains were white and retained much less **5a4** than their wild‐type strain with normal efflux activity. The Δ*bmrA* mutant retained more **5a4** and thus developed an intense pink color.

#### 2.4.3 Detection of Efflux Mediate Resistance in *Streptococcus pneumoniae*

**Figure 6:**
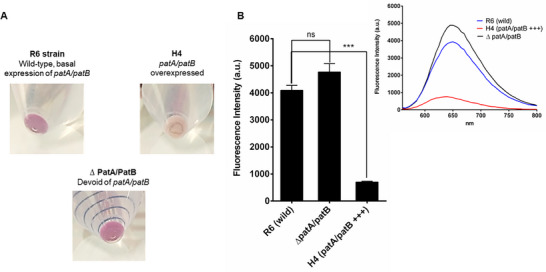
A) Visibly distinct colored cell pellets of *S. pneumonia* dependent on their efflux capacity after treatment of 5a4. B) The bar graph depicting the average fluorescence intensities of each pellet obtained by a fluorescence spectrometer. The inset picture depicts the emission spectra of each test strain scanned from 560 to 800 nm at an excitation of 530 nm.

*S. pneumoniae* is an important cause of community‐acquired respiratory infections. It was observed that active efflux mediated by PmrA or PatA/PatB imparted low‐level resistance to fluoroquinolones.^[^ ^36^ ^]^ The first genetic data suggests that the half‐ABC transporters SP2073 (PatB) and SP2075 (PatA) likely work in tandem to give intrinsic resistance.^[^ ^27^ ^]^ **Figure** **6**A shows that the pellets of the wild‐type strain (R6) and the ΔpatA/ΔpatB strain were visually indistinguishable. However, the H4 strain overexpressing patA/patB had a pale‐yellow hue. This implies that **5a4** had a low residential concentration and was efficiently removed from the H4 strain. The bar graph illustration (Figure 6B) suggests **5a4** could significantly, distinguish overexpressing *patA/patB* mutant from that of wild‐type (*p* < 0.001) and *patA/patB* knocked‐off mutant (*p* < 0.0001).

### 2.5 Interaction of Reserpine and 5a4

**Figure 7:**
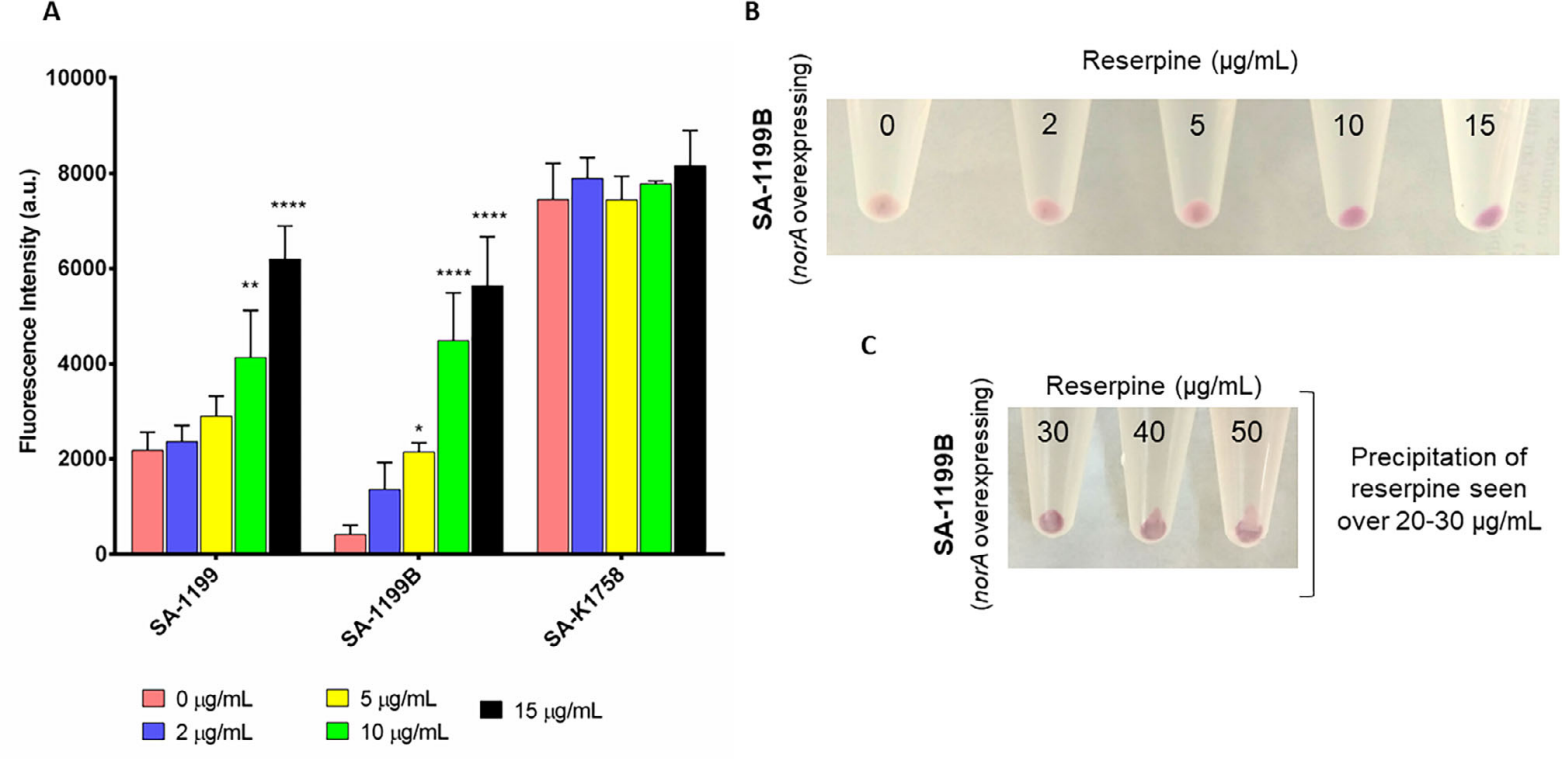
A) Bar graphs illustrating fluorescence intensities of SA‐1199, SA‐1199B, and SA‐K1758 subjected to 5a4 (1 µg mL^−1^) and increasing concentration of reserpine. B) Eppendorf tubes containing visibly distinct colored cell pellets of SA‐1199B (*norA* overexpressing) strain after the addition of 5a4 (1 µg mL^−1^) and increasing concentration of reserpine. The change of the pellet color from pale yellow to shades of pink hue can be observed. C) Precipitation of reserpine in the presence of 5a4 (1 µg mL^−1^) seen in tubes after increasing its concentration beyond 15 µg mL^−1^.

Reserpine was the first established plant‐based alkaloid inhibitor of the NorA efflux pump. Numerous investigations^[^ ^37^, ^38^, ^39^ ^]^ have demonstrated that reserpine caused at least a four‐fold increase in the efficacy of fluoroquinolones in efflux‐resistant strains of *S. aureus*. Utilizing reserpine's inhibitory effect on the NorA efflux pump, an experiment was designed to investigate whether **5a4** is pumped out through the NorA efflux pump, assessing its substrate specificity. As observed in **Figure** **7**A, an increase in fluorescent intensity was observed in the SA‐1199 and SA‐1199B strains. However, cells treated with reserpine at 10 µg mL^−1^ or higher showed a significant increase (*p* < 0.01) in fluorescence intensity compared to untreated cells.

There was a drastic increase in the fluorescent intensity of SA‐1199B. Since SA‐K1758, a *norA*‐deficient cell, showed no discernible change, it is likely that **5a4** is a substrate of the NorA pump and is efficiently pumped out via the NorA pump. In addition, it is evident from the color change observed in the pellets of SA‐1199B (from pale white to pink hue) after treatment of reserpine (Figure 7B) that it affects the efflux pump thereby causing an increase in **5a4**’s residence within the cytoplasm. Furthermore, it is apparent from the color shift in SA‐1199B pellets (from pale white to pink hue) after treatment with reserpine that it affects the efflux pump, increasing **5a4**’s cytoplasmic residency.

### 2.6 Molecular Validation of Interactions Between NorA and 5a4

**Figure 8:**
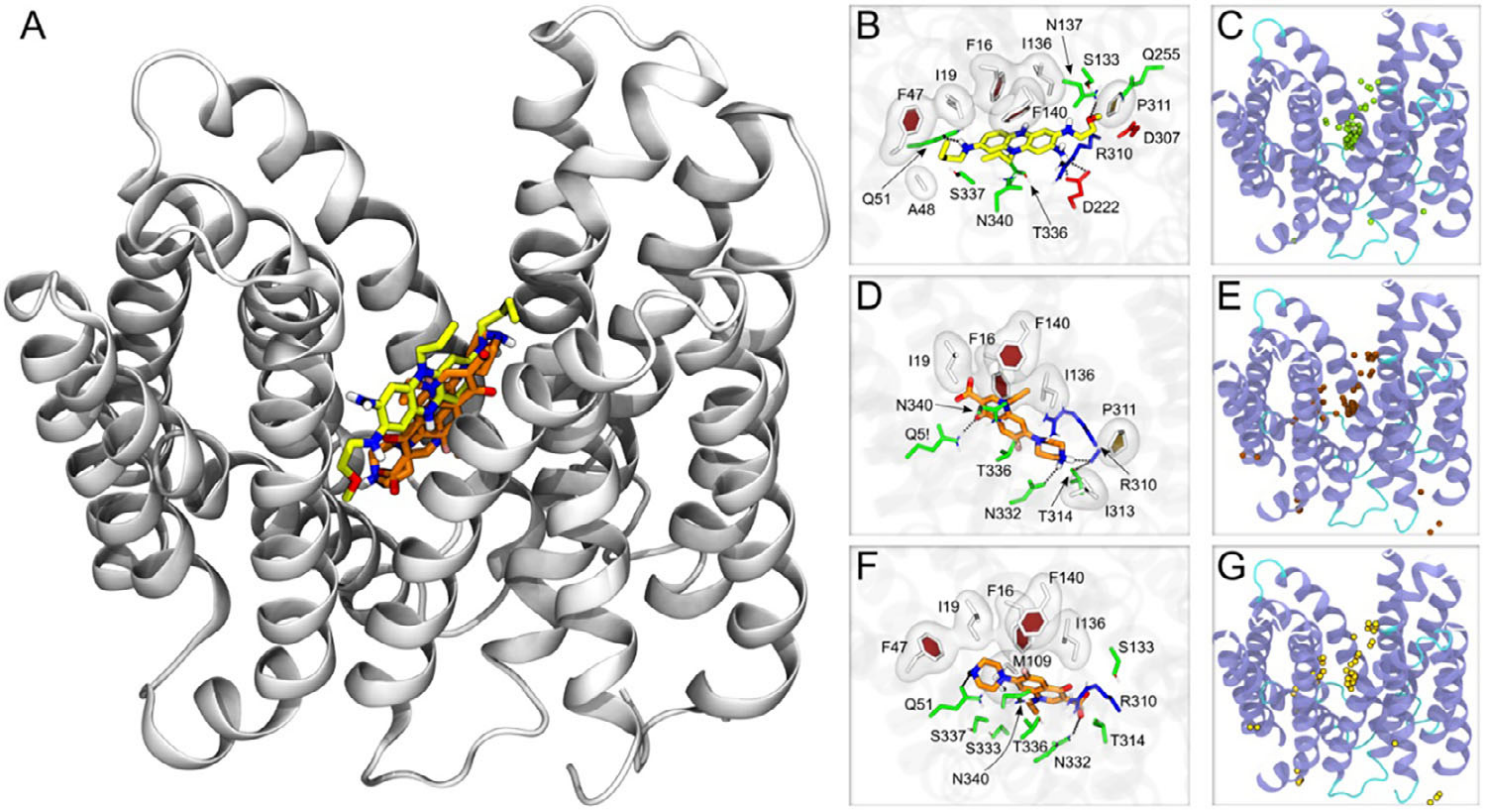
Molecular interactions between NorA and 5a4 and norfloxacin. A) Superposition of the top docking poses of 5a4 and norfloxacin on NorA. The protein is shown in grey ribbons, while the compounds are shown in sticks colored by atom type (N, O, H, F, and C in blue, red, white, pink, and yellow or orange for 5a4 or norfloxacin, respectively). Two poses are shown for norfloxacin, corresponding to neutral and anionic protomers. B,D,F) Details of the interactions between 5a4 (B), neutral (D), and anionic (F) norfloxacin. The compounds are shown as in (A), and the sidechains (or backbone when involved in H‐bonds with the ligands) are shown as thinner sticks colored by amino acid type (white, green, blue, and red for hydrophobic, polar, basic, and acidic residues, respectively). Hydrophobic residues are further shown with semi‐transparent surfaces. C,E,G) Distribution of all docking poses on NorA. The centers of mass of the poses are shown as spheres colored greenish yellow (C), dark orange (E), and light orange (G) for 5a4, neutral and anionic norfloxacin respectively.

To further validate the hypothesis that **5a4** is an effective substrate of NorA, we performed molecular docking calculations of this compound and of the well‐known substrate norfloxacin on the recently resolved experimental structures of NorA.^[^ ^40^ ^]^ **Figure** **8**A depicts the findings of this experiment, which reveal a significant overlap between the top poses of the two compounds. The detailed analysis of the binding poses confirms that **5a4** and norfloxacin interact similarly with the transporter, involving hydrophobic, polar, and basic amino acids to stabilize the complex (Figure 8B,D,F). The more extended structure of **5a4** allows this compound to interact also with acidic residues (Figure 8B). Overall, 9 (11) out of 12 (14) direct interactions made by the neutral (anionic) norfloxacin protomer with NorA are detected also for **5a4** (establishing 17 such interactions in the top docking pose). The agreement between the interaction patterns is conserved when considering the distribution of all the calculated binding poses of the two compounds on the transporter (Figure 8C,E,G). Finally, the docking scores of the top poses are comparable for the two compounds, being −6.7, −7.2, and −6.9 kcal mol^−1^ respectively for **5a4** and neutral or negatively charged norfloxacin. It is worth mentioning that our results are also in good agreement with previous literature reporting on the details of the interactions between NorA and reserpine and resveratrol^[^ ^28^ ^]^ or ciprofloxacin.^[^ ^41^ ^]^

### 2.7 Flow Cytometry

**Figure 9:**
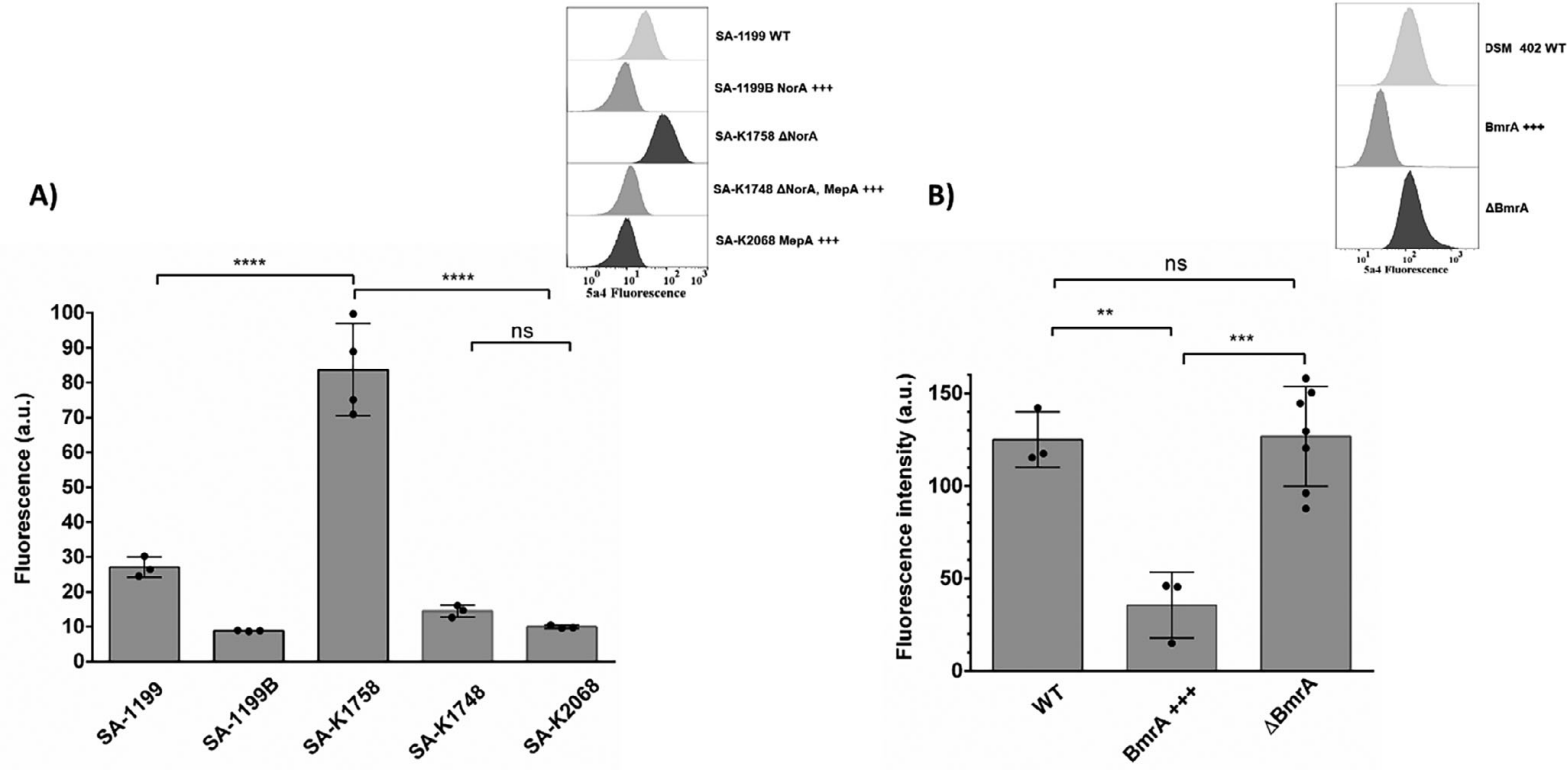
A) Intracellular level of 5a4 molecule in *S. aureus* wild‐type SA‐1199, norA overexpressing SA‐1199B, norA deleted SA‐K1758, MepA overexpressing strains SA‐K 1748 and SA‐K2068. Insert represents one example of the cytometry data with an x‐axis log scale B) Intracellular level of 5a4 in *Bacillus subtilis* WT, BmrA overexpressing and BmrA deleted strains. Cells were harvested in the mid‐exponential phase and fluorescence was measured at 615/25 nm. A total number of 300K cells were measured with 3–7 replicates. Statistical data were obtained using a One‐way ANOVA and a Tukey test.

The results obtained from using **5a4** as a diagnostic molecule were validated by using flow cytometry. With this technique, it is possible to monitor the fluorescence of hundreds of cells per second. Moreover, the **5a4** does not need to be removed from the experiment; instead, cells can remain continuously in equilibrium with a preset extracellular fluorochrome **5a4** concentration. As observed from **Figure** **9**A SA‐1199B; the overexpressing NorA strain exhibited about three‐fold less intracellular fluorescence (measured at 615/25 nm) compared to that of the wild‐type strain SA‐1199 which showed higher efflux due to the basal expression of the NorA pump. On the contrary, the SA‐K1758 strain devoid of NorA exhibited approximately three‐fold higher intracellular fluorescence of **5a4** compound as compared to the WT strain and nine‐fold compared to the SA‐1199B; NorA overexpressing strain. We found that these results were in strong compliance with that observed in Figure 4B. Strains SA‐K1748 and SA‐K2068 overexpressing the MepA in *S. aureus* also exhibit a lower amount of intracellular retention of **5a4** thereby confirming MepA's contribution towards actively effluxing out **5a4** molecules. Another Gram‐positive bacterium, *Bacillus subtilis*, was also tested with the **5a4** compound. Figure 9B shows that the strain overexpressing BmrA exhibits fourfold lower intracellular fluorescence compared to the WT strain, suggesting a higher efflux of **5a4** in the former. However, we were unable to detect a significant difference between the WT and the *BmrA*‐deleted strain in this experiment.

### 2.8 Clinical Strains

The set of *S. aureus* clinical strains tested in this study was previously evaluated by their efflux activity by the EtBrCW method and EtBr MIC determination. ^[^ ^20^, ^42^ ^]^ The first approach uses fluorescence generated by cultures swabbed on EtBr‐containing agar plates to determine each isolate's ability to expel EtBr from the cells via efflux. The complete characterization of each clinical strain is shown in Table S1 (Supporting Information). A total of 105 clinical strains and 10 reference strains, comprising both EtBrCW‐positive (efflux‐positive) and EtBrCW‐negative (efflux‐negative) strains, were evaluated for efflux activity using **5a4** and the resulting data from the two methods compared. The susceptibility profiles towards ciprofloxacin, norfloxacin, and moxifloxacin were determined or confirmed for all clinical strains.

**Figure 10:**
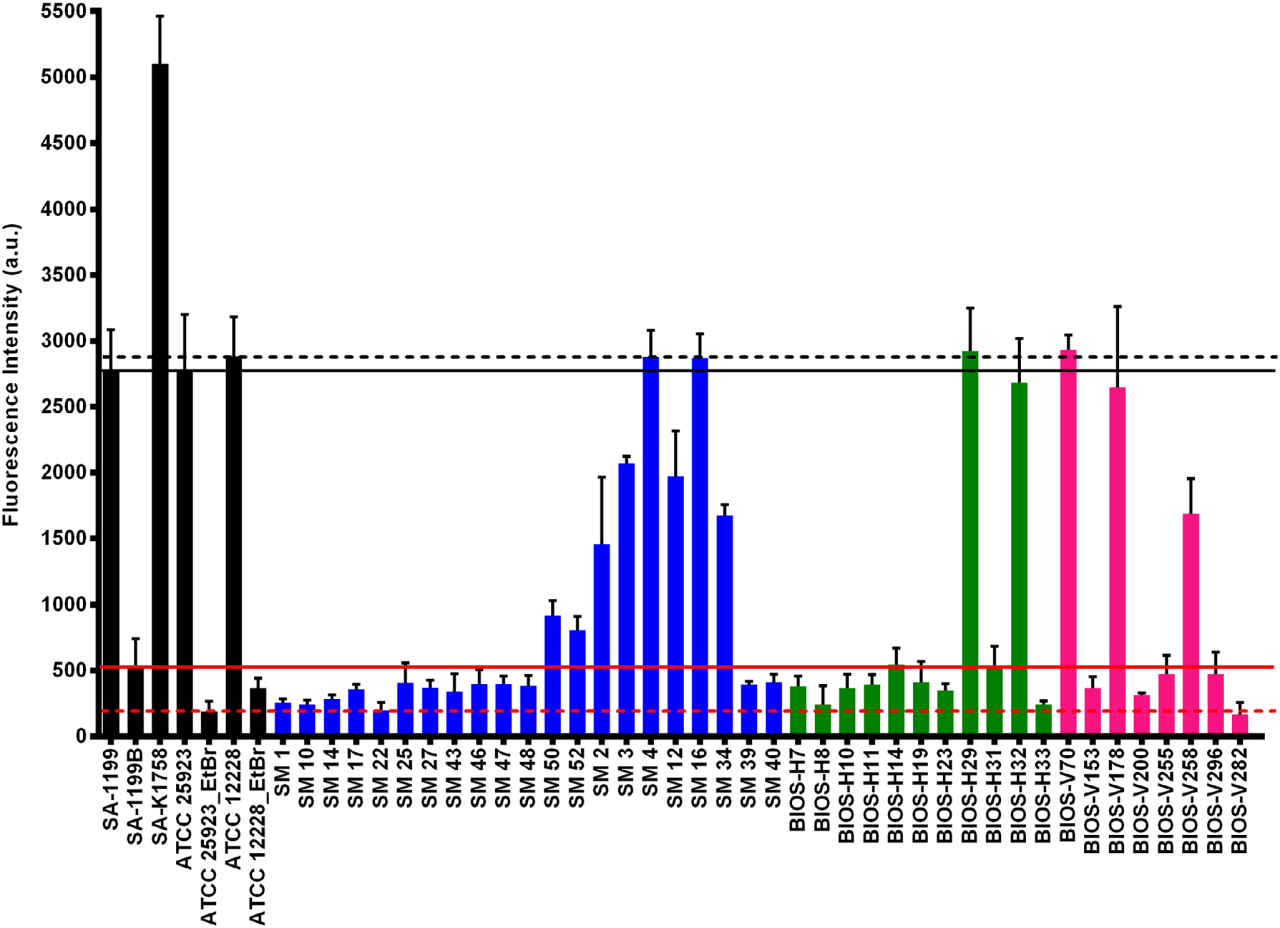
A. Bar graphs depicting fluorescence intensities of reference strains (black bars), clinical strains of human origin labeled as “SM” series (blue bars), clinical strains of human origin labeled as “BIOS‐H” series (green bars), and clinical strains of veterinary origin labeled as “BIOS‐V” series (pink bars). The black solid and dotted lines represent the fluorescence thresholds observed for the basal wild‐type strains of *S. aureus* and *S. epidermidis*, respectively. The red solid and dotted lines indicate the fluorescence thresholds for strains with increased efflux activity of *S. aureus* and *S. epidermidis*, respectively. These thresholds were established based on a lab‐designed NorA efflux mutant. Clinical strains exhibiting fluorescence intensities below or close to the red solid or dotted lines are suspected to have higher efflux activity.

Among the investigated clinical strains, we will describe the results of 40 strains in detail for the outcomes obtained by **5a4**. A bar graph **Figure** **10** illustrates the fluorescence intensities of clinical strains diagnosed using **5a4**. We used fluorescence responses from their respective wild‐type strains to determine a threshold for baseline efflux activity in *S. aureus* (solid black lines) and *S. epidermidis* (dotted black lines). The fluorescence response of known lab‐standard norA overexpressing mutants of *S. aureus* (solid red line) and *S. epidermidis* (dotted red line) was used to define the high efflux activity threshold. We anticipated that any response below or near the solid or dotted red lines would indicate the presence of efflux activity in the respective clinical strain. In the SM series, it was observed that thirteen clinical strains (SM 1, SM10, SM14, SM17, SM22, SM25, SM27, SM43, SM46, SM47, SM48, SM39, and SM40) were found to test positive using the **5a4** method. In the BIOS‐H series, nine strains, BIOS‐H7, H8, H10, H11, H14, H19, H23, H31, and H33, were found positive whereas, in BIOS‐V series, only four isolates, namely V153, V200, V255 and V296 were found to be positive for strong efflux.

Four *S. epidermidis* clinical strains of animal origin were also included in this screening, of which one, BIOS‐V282, was positive for efflux activity.

A good correlation was found between the two methods (**5a4** and EtBrCW method) for the SM series, whereas a higher capacity for identification of efflux activity was observed for the **5a4** method in the two BIOS series. In sum, the **5a4** method proved to be more sensitive in the detection of efflux activity.

## 3 Conclusion

Implementing rapid diagnostic technology in primary care settings holds great promise for enhancing the effectiveness and precision of therapeutic approaches. Our diagnostic molecule has the potential to revolutionize the identification of efflux‐mediated resistance, providing rapid and precise insights for critically ill patients and offering a transformative approach to managing antimicrobial resistance (AMR). The advantages of **5a4**, as detailed in this article, underscore its potential for both qualitative and quantitative assessments. Its seamless application could facilitate widespread use across medical testing facilities. We are confident that this molecule can offer clinicians valuable preliminary information on overactive efflux systems in isolated bacteria, guiding timely patient isolation and appropriate treatment decisions. **5a4** exhibited dose‐dependent cytotoxicity on the immortalized human keratinocyte cell line (HaCaT) (Section S4, Supporting Information), with significant viability reduction at higher concentrations. However, no clear genotoxicity was observed, suggesting cytotoxic effects without mutagenic potential. Slight phototoxicity was noted under UVA/visible light exposure, but it remained statistically insignificant. Handling recommendations are detailed in Section S5 (Supporting Information). Although our current tests have focused on a limited range of Gram‐positive bacteria, we are excited about the prospects of extending our research to include Gram‐negative bacteria. We anticipate that a mutant library may be necessary to fully confirm overexpression, but we are optimistic about the potential to overcome this challenge. Looking ahead, we are eager to test these diagnostic molecules and their derivatives against a broader spectrum of bacterial strains, paving the way for significant advancements in diagnostic accuracy and AMR management.

## 4 Experimental Section

### Bacteria, Media, and Chemicals

All *Staphylococcus aureus* strains were maintained and cultured using Mueller Hinton cation‐adjusted (MH‐II) agar and broth (Merck Millipore, USA) respectively. *Bacillus subtilis* strains were routinely grown at 37 °C in Luria Broth (LB) agar except the BmrA deleted strain that was grown in LB supplemented with 10 µg mL^−1^ of tetracycline. *Streptococcus pneumoniae* strains were grown and maintained in brain heart infusion agar supplemented with 5% defibrinated sheep blood. These cells were grown for 24 h at 37 °C in a 5% CO_2_ atmosphere. All other clinical strains mentioned in this article were grown and maintained in MH‐II agar and broth. Clinical strains tested are enlisted in Table S1 (Supporting Information). All the chemicals used in the present articles were purchased from Sigma Aldrich.

### Fluorescence Intensity Measurement

A stock solution of synthesized phenazinium compounds (10 mg mL^−1^) was prepared in DMSO solvent. The stock was gradually diluted with distilled water to achieve working stock (1 µg mL^−1^). An aliquot (100 µL) of this working stock was then added to the black flat bottomed, half‐area, 96‐well microplates (CELLSTAR, Greiner Bio‐One, France). The plate was then placed on a fluorescence spectrometer (Tecan, Infinite M200 Pro, Lyon, France) and scanned for emission spectra (560–800 nm) at a constant excitation of000A0;nm.

### Solution‐State Stability and Minimal Inhibitory Concentration (MIC)

Before accessing the minimal inhibitory concentration (MIC), each phenazinium derivative was subjected to a solution‐state stability test. For this experiment, each derivative was solubilized in DMSO to achieve a primary stock solution (20 mg mL^−1^). This stock was further diluted in MilliQ to achieve a working stock solution (10 mg mL^−1^). Later, the prepared working stock solution was serially diluted (512–1 µg mL^−1^) in MH‐II broth in an Eppendorf tube. The tubes were placed in an incubator at 37 °C for a day and later centrifuged at 25 000 rpm for 25 min. The tubes were observed for precipitation.

MIC was performed using micro‐broth dilution procedures following the Clinical and Laboratory Standards Institute (CLSI) guidelines M07‐A9.^[^ ^43^ ^]^ The broth microdilution tests were performed in sterile, flat‐bottomed, 96‐well microplates (Corning CoStar, Merck, Molsheim, France). The test was carried out using the BAC‐SCREEN platform in the lab. At a preliminary level, this test was conducted on *S. aureus* parent and efflux mutants namely SA‐1199, SA‐1199B, and SA‐K1758. Briefly, phenazinium derivatives were solubilized in DMSO and further diluted with sterile distilled water to obtain two‐fold serial dilutions ranging from 128 to 0.25 µg mL^−1^. The final test concentration consisted of <1% v/v DMSO. An aliquot (100 µL) of the prepared concentrations was transferred to the well of the microplate. Optimally, within 15 min of preparation, the inoculum was adjusted in MH‐II broth to obtain approximately 5 × 10^5^ CFU mL^−1^. The prepared inoculum (100 µL) was then transferred to each well of the microplate. The plates were incubated for 18 h at 37 °C. Since the compounds were colored an additional control plate was prepared by replacing the inoculum volume with MH‐II broth. MIC was determined by comparing the turbidity of the control plate and test plate. The lowest concentration of the phenazinium derivatives that prevented visible bacterial growth was regarded as the MIC.

### Assessing the Diagnostic Efficiency of Phenazinium Derivative 5a4 Against Gram‐Positive Bacterial Efflux Mutants

The protocol designed for assessing efflux activity in Gram‐positive bacteria is as follows:

- One or two colonies were taken from a freshly cultivated plate and inoculated in a conical flask containing MH‐II broth (20 mL). These flasks were placed in an orbital shaker incubator at 37 °C and 160 rpm for 3–4 h. (Note: This step was carried out to achieve cells in the mid‐exponential phase wherein the efflux pump is sufficiently active. Therefore, one must consider the growth curves of the bacteria in use).
- Following incubation, cells were transferred to 50 mL centrifuge tubes and spun for 10 min at 2600×g. The supernatant thus obtained was removed and fresh MH‐II broth was added to the pellet to achieve an optical density ranging from 2 to 4. (Note: Optical density should be constant for test samples and reference. Low density can interfere with the visibility of cell pellet).
- An aliquot (5 mL) of previously described optical density‐adjusted cell suspension was then transferred to a new centrifuge tube (15/50 mL). To this suspension, 5 µL of **5a4** stock solution (1000 µg mL^−1^) was added. The tubes were quickly capped and shaken to mix the compound (Note: Ideally, 50 mL tubes were preferred as they have greater surface area to aid shaking. Alternatively, one can prefer performing this step in a small conical flask too).
- The centrifuge tubes were then set aside on the table at room temperature for 15 min. (Caution should be taken not to incubate more than 15 min). Past 15 min of contact time, centrifuge tubes were spined down for 10 min at 2600 g and the supernatant containing **5a4** was discarded.
- The cell pellets were washed with 5 mL of phosphate buffer saline (PBS, pH 7) or potassium phosphate buffer (PPB, pH 7) and re‐pelleted by centrifugation (10 min at 2600 g). (Note: washing must be given with the help of 5 mL or 1 mL pipette. It should be ensured that no lumps of cells were left after washing. The purpose of washing is to remove excess **5a4** from the cell surface).
- PBS or PPB (1 mL) was added to the pellet and cells were re‐suspended. At this point:
  - For quantitative analysis, 100 µL of the prepared suspension was separated and adjusted to OD_600_ = 1. The OD‐adjusted suspension (100 µL) was then plated in the black half area 96 well plate and the fluorescence (Tecan, Infinite M200 Pro, Lyon, France) was read at excitation 530 nm and emission 645 nm. Results were computed using Magellan Pro 7.3 software.
  - The remaining 900 µL of aliquot can be centrifuged again to visualize the color of the cell pellets (with eyes) that corresponds to the state of their efflux activity as described below:
    - Cell pellet that exhibited white or off‐white hue (default color of cells) indicates an overexpressed efflux pump.
    - Cell pellet that exhibited a slight pink color indicates normal efflux activity.
    - Cell pellet that stained dark pink indicates low efflux activity or dysfunctional efflux pumps.
    - Alternatively, cell pellets may sometimes appear orange, indicating normal efflux activity. This happens because cells were inherently yellow. *S. aureus*, for example, produces the carotenoid pigment “staphyloxanthin”, which gives the cells their typical golden color.^[^ ^44^ ^]^

### Detection of Efflux Mediated Resistance in *Staphylococcus aureus*

Two sets of efflux mutants were examined in this investigation. In the first set, the efflux activity of NorA efflux mutants was detected. NorA was the most‐studied efflux pump in *S. aureus* belonging to the major facilitator superfamily (MFS). The protocol mentioned above was executed on strains SA‐1199B and SA‐K1758 (Table 1), featuring overexpression and inactivation of the *norA* gene, respectively. Wild‐type control strain SA‐1199, having basal *norA* expression and parent strain to SA‐1199B was also included in this study. The second set includes strains SA‐K1748 and SA‐K2068 having upregulation of the *mepA* gene. This gene belongs to the Multidrug and Toxic Compound Extrusion (MATE) family. In a published study,^[^ ^34^ ^]^ the authors confirmed the presence of upregulation of *mepA* in these strains. Additionally, strain SA‐ K1712 was included in this study as it was parent to SA‐K1748 (Table 1). The experiments were conducted in triplicates.

### Detection of Efflux Mediated Resistance in *Bacillus subtilis*

*Bacillus subtilis* was a commonly used model organism for research on multidrug ATP‐binding cassette (ABC) transporters. BmrA, previously known as YvcC, was a half‐size multidrug ABC transporter. This transporter exhibits the highest homology to each half of P‐gps (MDR1), HorA (40% identity), and LmrA (42% identity), which makes it an excellent choice to study multidrug ABC transporter.^[^ ^45^ ^]^ In the present study, compound **5a4** was tested on a few *B. subtilis* strains (Table 1) to detect the efflux activity. Briefly, *B. subtilis* wild‐type 168 (DSM 402), ΔBmrA (strain with deletion of *bmrA*), and BmrA++ (strain with overexpressed *bmrA* gene) were grown on LB plates, and the colonies so formed were suspended in fresh liquid MH‐II medium. Thereafter, previously described protocol designed for assessing efflux activity in Gram‐positive bacteria was performed.

### Detection of Efflux‐Mediated Resistance in *Streptococcus pneumonia*

*Streptococcus pneumoniae* ATP binding cassette (ABC) superfamily was composed of two transporters encoded by *patA* and *patB*. PatA/PatB was a deviant ABC transporter that has evolved to favor GTP as a cellular fuel.^[^ ^46^ ^]^ Overexpression of these two genes was also reported in ciprofloxacin‐resistant or linezolid‐resistant laboratory‐selected mutants as well as in clinical isolates. ^[^ ^27^ ^]^ Strain H4 showed higher levels of expression of both *patA* and *patB* than R6. Therefore, this experiment was performed on wild‐type *S. pneumoniae* strain (R6; Parent, basal *patA/patB*), strain overexpressing *patA/patB* (H4), and strain devoid of a functional *patA*/*patB* (Δ*patA*/Δ*patB*) (Table 1). Since these strains were anaerobic, they were grown statically in 50 mL of brain heart infusion broth supplemented with 5% defibrinated sheep blood under anaerobic conditions. After the culture attained OD_600_ = 0.6, cells were harvested and re‐suspended in fresh liquid MH‐II broth to conduct the protocol. Three sets of independent experiments were performed.

### Interaction of Reserpine and 5a4

The purpose of this investigation was to confirm if the efflux of **5a4** in *S. aureus* was instigated by the efflux pump protein NorA. To verify the above, new cell suspensions (OD600 = 2) in MH‐II of the *S. aureus* parental and *norA* efflux mutant strains SA1199, SA1199B, and SA‐K1758 were made. Several aliquots of each prepared cell suspension (1.9 mL) were added to Eppendorf tubes. These aliquots were subsequently exposed to reserpine, a recognized NorA pump efflux inhibitor, ^[^ ^47^ ^]^ at increasing concentrations (1 to 50 µg mL^−1^). Post‐addition of reserpine, the volume of cell suspension was made up to 2 mL with MH‐II broth. An aliquot of **5a4** (final concentration 1 µg mL^−1^) was added to each Eppendorf tube and allowed to sit on the table for 15 min. After contact time, the suspension was centrifuged and washed with PBS according to the protocol described before.

### Molecular Validation of Interaction Between NorA and 5a4

To investigate the molecular mechanism of action by **5a4**, blind docking calculations of this compound and of norfloxacin on NorA were performed using the software VINA1.2.^[^ ^48^ ^]^ The molecular structure of **5a4** was generated from a 2D sketch using MarvinSketch (version 14.9.1.0, calculation module developed by ChemAxon) and further optimized with the Gaussian 16 suite of programs^[^ ^49^ ^]^ in the presence of implicit water solvent [keyword: #b3lyp/6‐31G(d, p) opt(tight) scf(MaxCycle = 512) scrf = (pcm, solvent = water) freq]. The molecular structures of norfloxacin both neutral and with net charge ‐1 were taken respectively fromMalloci et al.,^[^ ^50^ ^]^ and Gervasoni et al.,^[^ ^51^ ^]^ Semiflexible blind docking was performed, in which 11 and 3 torsional angles were allowed to rotate for **5a4** and norfloxacin respectively. Two recently published experimental structures of NorA in complex with synthetic antigen‐binding fragments (Fabs)^[^ ^40^ ^]^ were used to account for the limited flexibility of the transporter (PDB IDs 7LO7 and 7LO8).

The ad4 scoring function was used to find and rank docking poses, and to improve sampling the exhaustiveness was set to 1024 (default 8). Up to 50 poses were generated for each NorA conformation, searched within a 0.375 Å spaced grid of dimensions 1.15 times those of NorA along x, y, and z.

### Flow Cytometry

The results obtained from using **5a4** as a diagnostic molecule were validated by flow cytometry. This experiment was conducted on NorA and MepA efflux mutants of *S. aureus*. Briefly, freshly cultured mid‐exponential phased cells were harvested and re‐suspended to 2 mL fresh MH‐II at OD_600_ = 1. The entire procedure was performed in an Eppendorf tube (2 mL) for flow cytometry as not many cells were required. **5a4** (1 µg mL^−1^) was added to each tube and kept aside for 15 min. Cells were centrifuged and pellets were adjusted to 1–5 × 10^6^ cells mL^−1^ in PBS. The cells were first analyzed for their light scattering (forward scattering; FSC versus side scattering SSC signal): the density plot obtained was first gated on the population of cells and then filtered to remove multiple events. **5a4** labeling was followed by fluorescence (FL3: 615/25 nm) and was quantified in each sample. The low auto‐fluorescence level of the unstained sample was subtracted from the total **5a4** treated samples. A total number of 300 K events were collected per sample and data analyses were carried out with at least three replicate samples. Data were acquired with a Bio‐Rad S3E cells sorter using 488 and 561 nm lasers and were analyzed using FlowJo v10.6. Statistical analyses were conducted with Prism.v8.2 to estimate the significance level using ANOVA tests.

### Clinical Strains

Rapid diagnostic ability of **5a4** to identify efflux‐mediated resistant bacteria was performed in Instituto de Higiene de Medicina Tropical, Universidade NOVA de Lisboa (IHMT‐NOVA) using the optimized protocol (section 3.3). Clinical *Staphylococcus* strains were sourced from human and veterinary samples. These strains were pre‐analyzed and published by the IHMT group (details in Table S1, Supporting Information). All the clinical isolates were evaluated for their efflux activity using the EtBrCW method and by determining the MICs of EtBr and effluxable antibiotics viz. Ciprofloxacin (CIP), norfloxacin (NOR) and Moxifloxacin (MFX). Isolates classified as EtBrCW‐positive or EtBrCW‐negative were screened for the presence of chromosomal mutations most commonly associated with fluoroquinolone resistance in *S. aureus*, namely the ones occurring in the QRDRs of both *grlA* and *gyrA* genes. Efflux activity was investigated using the **5a4** protocol. Both qualitative and quantitative analyses were performed and their results were compared to those obtained with the EtBrCW method. As controls, two pairs of isogenic strains differing in *norA* expression were included, *S. aureus* ATCC 25923 and its progeny adapted to EtBr, *S. aureus* ATCC 25923_EtBr,^[^ ^52^ ^]^ as well as *S. epidermidis* ATCC 12228 and its progeny adapted to EtBr, *S. epidermidis* ATCC 12228_EtBr.^[^ ^53^ ^]^

### Statistical Analysis

Graphs were plotted using GraphPad Prism 6 (GraphPad Inc.). Multiple groups were compared by an ordinary one‐way ANOVA by Tukey's multiple comparisons test. The p‐reported suggests (∗*p* < 0.05; ∗∗*p* < 0.01; ∗∗∗*p* < 0.001; ∗∗∗∗*p* < 0.0001 and ns stands for non‐significant).

## Conflict of Interest

The authors declare the following conflict of interest: Jean‐Michel Bolla, along with Olivier Siri, Mrunal Patil, Michel Camplo, Serhii Krykun, Jean‐Manuel Raimundo, Frédéric Garzino, Eric Garnotel, and Frédéric Brunel, are cited as inventor in a patent (French Patent Number: FR3132099A1, granted on December 8, 2023, as well as international patent application PCT/EP2023/051578) related to the topic discussed in this manuscript.

## Supporting information



Supporting Information

## Data Availability

The data that support the findings of this study are available from the corresponding author upon reasonable request.
