# Supplementary material for: Unlocking the Gates: A Novel Diagnostic Molecule for Quantifying Efflux Levels in Gram‐Positive Bacteria
Source: Adv Healthc Mater. 2025 Mar 11;14(11):2404145. doi: 10.1002/adhm.202404145 (PMC12023841; doi:10.1002/adhm.202404145)
Supplement: Supplementary file 1 — Supporting Information [file ADHM-14-0-s001.docx]

**Supporting Information**

**Unlocking the Gates: A Novel Diagnostic Molecule for Quantifying Efflux Levels in Gram-Positive Bacteria**

*Mrunal Patil, Tatiana Munteanu, Gaël Brasseur, Carolina Ferreira, Sofia Santos Costa, Isabel Couto, Mohd Athar, Elisa Asunis, Attilio Vittorio Vargiu, Miguel Viveiros, Carole DiGiorgio, Frédéric Brunel, Jean-Manuel Raimundo, Michel Camplo, Olivier Siri*, Jean-Michel Bolla**

**TABLE OF CONTENT**

1. [General Remarks and Analysis Conditions 1](file:///C:\Users\stagimmf\Desktop\support.docx#_Toc159410739)
2. [Synthetics Protocols and Characterization 2](file:///C:\Users\stagimmf\Desktop\support.docx#_Toc159410740)

2.1.[NMR Spectra 20](file:///C:\Users\stagimmf\Desktop\support.docx#_Toc159410741)

2.2. [Mass Spectrometry 64](file:///C:\Users\stagimmf\Desktop\support.docx#_Toc159410742)

1. [Strains Related Supplementary Information …73](file:///C:\Users\stagimmf\Desktop\support.docx#_Toc159410743)
2. In-vitro cytotoxicity test and micronucleus assay on HaCaT keratinocytes cells……….78
3. Safety handling recommendations………………………….……………………………79
4. [References…………………………………………………………………………….….80](file:///C:\Users\stagimmf\Desktop\support.docx#_Toc159410744)

1. **General Remarks and Analysis Conditions**
   1. ***Reagents:*** Derivatives **2e, 3e, 4e2** and **4e3, OOtB and OOO** were synthesised according to literature protocols. ^[1]^ All reagents and solvents were purchased from Alfa-Aesar or Sigma-Aldrich and used as received. When heating was required, oil baths were used. Column chromatography was performed using silica gel (60-120 mesh) and alumina 90 neutral (63−200 μm, Beckmann grade I). Analytical thin layer chromatography (TLC) was performed on precoated silica gel-60 F254 (0.5 mm) aluminium plate or precoated Al_2_O_3_ gel-60 neutral (0.2mm) aluminium plate. Visualisation of the spots on TLC plates was achieved by exposure to UV light. Filter aid was performed using Celite AW standard Supercel or Celite type 545. Unless otherwise specified, the desired product was dried under vacuum (<10 mbar) over 5 h at room temperature.
   2. ***Analytical Methods and Apparatus.*** NMR spectra were recorded at room temperature on a JEOL ECS400 spectrometer operating at 400 MHz for ^1^H and 100 MHz for ^13^C respectively. NMR chemical shifts are given in ppm (δ) relative to Me_4_Si with solvent resonances used as internal standards (CDCl_3_: 7.26 ppm for ^1^H and 77.2 for ^13^C; CD_3_OD: 3.31 ppm for ^1^H and 49.0 for ^13^C). The multiplicity of signals is designated by the following abbreviations: s, singlet; br s, broad singlet; d, doublet; t, triplet; q, quartet; quint, quintet; sext, sextet; m, multiplet, br m, broad multiplet. Coupling constants, *J*, are reported in Hertz (Hz). High-resolution mass spectrometry (HRMS) analysis was performed by the “Spectropole” of Aix-Marseille University.
2. **Synthetics Protocols and Characterization**

**1a**

To a stirred solution of DFNB (2 mL, 2.9 g, 18.23 mmol, 1 equiv.) in DCM (50 mL) at 0 °C, was added propylamine (1.5 mL, 1.08 g, 18.23 mmol, 1 equiv.), followed by addition of DIPEA (3.49 mL, 2.6 g, 20 mmol, 1.1 equiv.). The reaction mixture was allowed to warm up to room temperature (over 30 min), after which time the reaction was complete (monitored by TLC). The solvent was evaporated in vacuo to afford a yellow residue that was purified over silica gel column gel chromatography (petroleum ether/ ethyl acetate, 96/4; Rf = 0.5) to afford the desired product as a yellow oil (2.74 g, 13.8 mmol, 76%).

**^1^H NMR** (**400 MHz, CDCl_3_):** δ = 8.17 (m, 2H), 6.47 (dd, *J* _=_ 11.6 Hz, *J* = 2.4 Hz, 1H), 6.35 (ddd, *J* _=_ 9.6 Hz, 4.8 Hz, 2.4 Hz, 1H), 3.22 (td, *J*  = 7 Hz, *J* = 5.2 Hz, 2H), 1.76 (sext, *J* = 7.3 Hz, 2H), 1.06 (t, *J*  = 7.4 Hz, 3H). **^13^C NMR** (**101 MHz, CDCl_3_**)**:** δ = 167.7 (d, *J_C-F_* = 257 Hz), 147.7 (d, *J_C-F_* = 14 Hz), 130.1 (d, *J_C-F_* = 13 Hz), 128.8, 103.9 (d, *J_C-F_* = 25 Hz), 99.3 (d, *J_C-F_* = 27 Hz), 45.1, 22.1, 11.6.

**1b**

To a stirred solution of DFNB (2 mL, 2.9 g, 18.23 mmol, 1 equiv.) in DCM (50 mL) at 0 °C, was added octylamine (3 mL, 2.35 g, 18.23 mmol, 1 eq.), followed by addition of DIPEA (3.49 mL, 20 mmol, 2.6 g, 1.1 eq.). The reaction mixture was allowed to warm up to room temperature (over 30 minutes), after which the reaction was complete (monitored by TLC). The solvent was evaporated under vacuum to afford a yellow residue that was purified over silica gel column gel chromatography (petroleum ether/ ethyl acetate, 96/4; Rf = 0.5) to afford the desired product as a yellow oil (4.19 g, 15.6 mmol, 86%).

**^1^H NMR** (**400 MHz, CDCl_3_):** δ = 8.21 (m, 2H), 6.46 (dd, *J* = 11.6 Hz, *J* = 2.4 Hz, 1H), 6.34 (ddd, *J* = 9.6 Hz, 4.8 Hz, 2.6 Hz, 1H), 3.24 (q, *J* = 6.4 Hz, 2H), 1.73 (quint, *J* = 7.2 Hz, 2H), 1.44 (m, 2H), 1.29 (m, 8H), 0.89 (t, *J* = 6.8 Hz, 3H). **^13^C NMR** (**101 MHz, CDCl_3_**)**:** δ = 168.4 (d, *J*_C-F_ = 258 Hz), 148.4 (d, *J*_C-F_ = 13 Hz), 130.9 (d, *J*_C-F_ = 13 Hz), 129.5, 104.6 (d, *J*_C-F_ = 25 Hz), 100.1 (d, *J*_C-F_ = 27 Hz), 44.1, 32.6, 30.1, 30.0, 29.5, 27.9, 23.5, 14.9.

**2a**

To a solution of **1a** (1.216 g, 6.13 mmol, 1 equiv.) in MeCN (20 mL) was added butylamine (1.2 mL, 12.27 mmol, 2 equiv.) and DIPEA (1.8 mL, 10.42 mmol, 1.7 equiv.). The reaction mixture was stirred at 80 °C overnight. Then the solvent was evaporated and the residue was taken up into EtOAc (70 mL), washed with saturated ammonium chloride aqueous solution (2 x 700 mL) and brine (45 mL). The combined organic layers were then dried over Na_2_SO_4_, filtered and evaporated under vacuum. The obtained crude was purified over silica gel column gel chromatography (petroleum ether/ ethyl acetate, 9/1 to 8/2; R_f_ (petroleum ether/ ethyl acetate, 8/2) = 0.56) to afford the desired product as an orange oil (1.36 g, 5.41 mmol, 88 %).

**^1^H NMR (400 MHz, CDCl_3_):** δ = 8.52 (br s, 1H), 7.99 (d, *J*  = 9.2 Hz, 1H), 5.89 (d, *J* = 9.6 Hz, 1H), 5.61 (s, 1H), 4.43 (br s, 1H), 3.18 (m, 4H), 1.75 (sext, *J* = 7.2 Hz, 2H), 1.62 (quint, *J* = 7.2 Hz, 2H), 1.43 (sext, *J* = 7.5 Hz, 2H), 1.04 (t, *J* = 7.4 Hz, 3H), 0.96 (t, *J* = 7.2 Hz, 3H).**^13^C NMR (101 MHz, CDCl_3_):** δ = 154.5, 148.7, 129.3, 123.7, 104.8, 90.0, 44.8, 43.0, 31.3, 22.2, 20.3, 13.9, 11.8.

**2b**

To a solution of **1b** (3 g, 11.2 mmol, 1.0 equiv.) in THF (30 mL) was added butylamine (2.45 g, 3.32 mL, 33.5 mmol, 3 equiv.) and DIPEA (2.9 g, 3.9 mL, 22.4 mmol, 2 equiv.). The reaction mixture was stirred at 80 °C overnight. Then the solvent was evaporated, and the residue was taken up into ethyl acetate (150 mL), washed with saturated NH_4_Cl *aq.* solution (2 x 150 mL) and brine (100 mL). The combined organic layers were then dried over MgSO_4_, filtered and evaporated under vacuum. The obtained crude was purified over silica gel column gel chromatography (petroleum ether/ ethyl acetate, 98/2; Rf = 0.2) to afford the desired product as a yellow oil (1.9 g, 5.9 mmol, 53% yield).

**^1^H NMR (400 MHz, CDCl_3_):** δ = 8.53 (br s, 1H), 8.02 (d, *J* = 9.6 Hz, 1H), 5.88 (dd, *J* = 10 Hz, 2.4 Hz, 1H), 5.61 (d, *J* = 2.4 Hz, 1H), 4.39 (t, *J* = 4.4 Hz, 1H), 3.21 (m, 4H), 1.72 (quint, *J* = 7.6 Hz, 2H), 1.64 (quint, *J* = 7.6 Hz, 2H), 1.44 (sext, *J* = 7.6 Hz, 4H), 1.31 (m, 8H), 0.98 (t, *J* = 7.4 Hz, 3H), 0.88 (t, *J* = 6.8 Hz, 3H). **^13^C NMR (101 MHz, CDCl_3_):** δ = 154.5, 148.7, 129.3, 123.5, 104.9, 89.8, 43.0 (2C overlapped), 31.9, 31.2, 29.4, 29.3, 28.8, 27.2, 22.7, 20.3, 14.2, 13.9.

**2c**

To a pressure bomb, were introduced DFNB (2 mL, 18.23 mmol, 1 equiv.), 1‑propylamine (6.14 mL, 74.7 mmol, 4.1 equiv.) and DIPEA (5.4 mL, 31 mmol, 1.7 equiv.). The bomb was closed with a Teflon seal. The mixture was heated to 145 °C for 3 hrs. After cooling to room temperature, EtOH (5 mL) was added. This suspension was scatted with ultrasound. The resulting solid in the suspension was isolated by filtration, rinsed with hot water and dried under vacuum to afford the desired product as a yellow powder (4.07 g, 17.14 mmol, 94%).

**^1^H NMR (400 MHz, CDCl_3_):** δ = 8.53 (br s, 1H), 8.01 (d, *J* = 8.8 Hz, 1H), 5.90 (dd, *J* = 9.2 Hz, 2 Hz, 1H), 5.63 (d, *J* = 2 Hz, 1H), 4.46 (br s, 1H), 3.18 (m, 4H), 1.75 (sext, *J* = 7.2 Hz, 2H), 1.68 (sext, *J* = 7.6 Hz, 2H), 1.05 (t, *J* = 7.4 Hz, 3H), 1.02 (t, *J* = 7.4 Hz, 3H). **^13^C NMR (101 MHz, CDCl_3_):** δ = 154.5, 148.7, 129.3, 123.6, 104.8, 89.2, 45.1, 44.8, 22.4, 22.1, 11.8, 11.6.

**2d**

To a pressure bomb, were introduced DFNB (2 mL, 2.9 g, 18.23 mmol, 1 equiv.), 1‑butylamine (5.46 g, 7.38 mL, 74.7 mmol, 4.1 equiv.) and DIPEA (4 g, 5.4 mL, 31 mmol, 1.7 equiv.). The bomb was closed with a Teflon seal. The mixture was heated to 145 °C for 3 hours. After cooling to room temperature, EtOH (5 mL) was added. This suspension was scatted with ultrasound. The resulting solid in the suspension was isolated by filtration, rinsed with hot water and dried under vacuum to afford the desired product as a yellow powder (4.5 g, 17 mmol, 93% yield).

**^1^H NMR (400 MHz, CDCl_3_):** δ = 8.52 (br s, 1H), 8.02 (d, *J* = 9.2 Hz, 1H), 5.89 (dd, *J* = 9.6 Hz, 2.4 Hz, 1H), 5.62 (d, *J* = 2.4 Hz, 1H), 4.38 (br s, 1H), 3.21 (m, 4H), 1.72 (quint, *J* = 7.2 Hz, 2H), 1.64 (quint, *J* = 7.6 Hz, 2H), 1.43 (m, 4H), 0.98 (t, *J* = 7.4 Hz, 3H), 0.97 (t, *J* = 7.4 Hz, 3H). **^13^C NMR (101 MHz, CDCl_3_):** δ = 154.5, 148.7, 129.4, 123.7, 104.7, 89.9, 43.0, 42.7, 31.3, 30.9, 20.4, 20.3, 13.9 (2C overlapped).

**2e**

To a pressure bomb, were introduced DFNB (4.72 g, 29.5 mmol, v = 3.25 mL, 1.0 equiv.), 1-octylamine (15.65 g, 121 mmol, 20 mL, 4.1 equiv.) and DIPEA (11.85 g, 9 mL, 51.7 mmol, 1.7 equiv.). The bomb was closed with a Teflon seal. The mixture was heated to 145 °C for 3 hours. After cooling to room temperature, EtOH (10 mL) was added. This suspension was scatted with ultrasound. The resulting solid in the suspension was isolated by filtration, rinsed with hot water and dried under vacuum to afford the desired product as a yellow powder (10.7 g, 28.3 mmol, 96% yield).

**^1^H NMR (400 MHz, CDCl_3_):** δ = 8.53 (br s, 1H), 8.01 (d, *J* = 9.6 Hz, 1H), 5.89 (dd, *J* = 9.6 Hz, 2 Hz, 1H), 5.61 (d, *J* = 2 Hz, 1H), 4.40 (t, *J* = 4.6 Hz, 1H), 3.20 (m, 4H), 1.72 (quint, *J* = 7.2 Hz, 2H), 1.65 (m, 4H), 1.47-1.22 (br m, 18H), 0.88 (t, *J* = 7.2 Hz, 6H). **^13^C NMR (101 MHz, CDCl_3_):** δ = 154.5, 148.7, 129.3, 123.6, 104.8, 89.8, 43.3, 43.0, 31.9, 29.4, 30.0, 29.9, 29.3, 29.2, 28.9, 27.3, 27.1, 22.7, 14.2. Three aliphatic carbon signals overlapping. NMR characterization was comparable to the previously reported results.

**General procedure for compounds 3a-e**

A solution of **2a-e** (5.41 mmol, 1 equiv.) in THF (or MeOH where indicated) (70 mL) was hydrogenated (P = 40 bars) in the presence of Pd/C (5% wt, % 1 mol) overnight. After reducing the pressure, the solution was degassed by bubbling Argon in the mixture. 1,5-difluoro-2,4-dinitrobenzene (5.14 mmol, 0.95 equiv.) was added at 0 °C under stirring and the conditions kept for 30 min. The end of the reaction was detected by TLC. Pd/C was then removed by filtration through Celite® plug. The crude product was purified over silica gel column chromatography to afford the desired product as a brown-reddish solid.

**3a**

Reaction in MeOH. Yield 81%. **R_f_** (DCM) = 0.4

**^1^H NMR** (**400 MHz, CDCl_3_):** δ = 9.31 (br s, 1H), 9.14 (d, *J* = 8.2 Hz, 1H), 6.82 (d, *J* = 8.4 Hz, 1H), 6.52 (d, *J* = 13.6 Hz, 1H), 5.99 (d, *J* = 8.4 Hz, 1H), 5.96 (s, 1H), 3.77 (br s, 1H), 3.70 (br s, 1H), 3.13 (t, *J* = 7.2 Hz, 2H), 3.06 (m, 1H), 1.67-1.51 (m, 4H), 1.46 (sext, *J* = 7.4 Hz, 2H), 0.97 (t, *J* = 7.4 Hz, 3H), 0.92 (t, *J* = 7.4 Hz, 3H). **^13^C NMR (101 MHz, CDCl_3_):** δ = 161.3 (d, *J_C-F_* = 271 Hz), 150.6 (d, *J_C-F_* = 13 Hz), 150.3, 145.2, 128.7, 127.8, 127.0, 126.9, 110.8, 104.1 (d, *J_C-F_* = 27 Hz), 101.7, 95.2, 45.3, 43.6, 31.7, 22.6, 20.4, 14.0, 11.7.

**3b**

Reaction in MeOH. Yield 99%. **R_f_** (petroleum ether/ ethyl acetate, 9/1) = 0.3

**^1^H NMR** (**400 MHz, CDCl_3_):** δ = 9.31 (s, 1H), 9.16 (d, *J* = 7.6 Hz, 1H), 6.84 (d, *J* = 8.4 Hz, 1H), 6.54 (d, *J* = 13.6 Hz, 1H), 6.02 (dd, *J* = 8.4 Hz, 2.4 Hz, 1H), 5.95 (d, *J* = 2.4 Hz, 1H), 3.76 (br s, 1H), 3.66 (t, *J* = 5 Hz, 1H), 3.14 (t, *J* = 7.2 Hz, 2H), 3.08 (q, *J* = 6.4 Hz, 2H), 1.64 (quint, *J* = 7.3 Hz, 2H), 1.56 (m, 2H), 1.46 (sext, *J* = 7.2 Hz, 2H), 1.34-1.20 (br m, 10H), 0.99 (t, *J* = 7.2 Hz, 3H), 0.87 (t, *J* = 6.8 Hz, 3H). **^13^C NMR (101 MHz, CDCl_3_):** δ = 162.0 (d, JC-F = 272 Hz), 151.3 (d, JC-F = 13 Hz), 151.0, 145.9, 129.3, 128.5, 127.7 (d, *J*_C-F_ = 10 Hz), 111.5, 104.8 (d, *J*_C-F_ = 27 Hz), 102.4, 95.9, 44.4, 44.3, 32.6, 32.4, 30.1 (2C overlapped), 30.0, 27.9, 23.4, 21.1, 14.9, 14.7. One aromatic carbon obscuring or overlapping.

**3c**

 Yield 73%. **R_f_** (DCM/ petroleum ether, 6/4) = 0.5

**^1^H NMR** (**400 MHz, CDCl_3_):** δ = 9.31 (s, 1H), 9.15 (d, *J* = 8 Hz, 1H), 6.84 (d, *J* = 8.4 Hz, 1H), 6.55 (d, *J* = 13.6 Hz, 1H), 6.02 (dd, *J* = 8.4 Hz, 2.4 Hz, 1H), 5.97 (d, *J* = 2.4 Hz, 1H), 3.80 (br s, 1H), 3.70 (br s, 1H), 3.12 (t, *J* = 7 Hz, 2H), 3.09 (m, 2H), 1.69 (sext, *J* = 7.2 Hz, 2H), 1.59 (sext, *J* = 7.2 Hz, 2H), 1.03 (t, *J* = 7 Hz, 3H), 0.92 (t, *J* = 7.4 Hz, 3H). **^13^C NMR (101 MHz, CDCl_3_):** δ = 161.3 (d, *J*_C-F_ = 272 Hz), 150.6 (d, *J*_C-F_ = 13 Hz), 150.3, 145.2, 129.6, 127.8, 127.0 (d, *J*_C-F_ = 9 Hz), 110.8, 104.1 (d, *J*_C-F_ = 27 Hz), 101.7, 95.2, 45.8, 45.3, 22.8, 22.6, 11.8, 11.7.

**3d**

 Yield 80%. **R_f_** (DCM) = 0.4

**^1^H NMR** (**400 MHz, CDCl_3_):** δ = 9.30 (s, 1H), 9.16 (d, *J* = 8 Hz, 1H), 6.84 (d, *J* = 8.4 Hz, 1H), 6.55 (d, *J* = 13.6 Hz, 1H), 6.02 (dd, *J* = 8.4 Hz, 2.4 Hz, 1H), 5.96 (d, *J* = 2.4 Hz, 1H), 5.3 (traces of DCM), 3.77 (br s, 1H), 3.66 (t, *J* = 5.2 Hz, 1H), 3.15 (t, *J* = 5.2 Hz, 2H), 3.09 (q, *J* = 6.7 Hz, 2H), 2.17 (traces of acetone), 1.64 (quint, *J* = 7.4 Hz, 2H), 1.54 (quint, *J* = 7.2 Hz, 2H), 1.46 (sext, *J* = 7.4 Hz, 2H), 1.33 (sext, *J* = 7.2 Hz, 2H), 0.99 (t, *J* = 7.2 Hz, 3H), 0.92 (t, *J* = 7.2 Hz, 3H). **^13^C NMR (101 MHz, CDCl_3_):** δ = 161.2 (d, *J_C-F_* = 272 Hz), 150.6 (d, *J_C-F_* = 13 Hz), 150.3, 145.2, 128.6, 127.8, 127.0 (d, *J_C-F_* = 9 Hz), 110.8, 104.1 (d, *J_C-F_* = 27 Hz), 101.6, 95.2, 43.6, 43.2, 31.7, 31.5, 20.4, 20.3, 14.0, 13.9. One aromatic carbon signal obscured or overlapping.

**3e**

 Yield 95%. **R_f_** (DCM) = 0.6

**^1^H NMR** (**400 MHz, CDCl_3_):** δ = 9.30 (s, 1H), 9.16 (d, *J* = 7.6 Hz, 1H), 6.84 (d, *J* = 8.4 Hz, 1H), 6.55 (d, *J* = 13.6 Hz, 1H), 6.01 (dd, *J* = 8.4 Hz, 2.4 Hz, 1H), 5.96 (d, *J* = 2.4 Hz, 1H), 3.77 (br s, 1H), 3.67 (br s, 1H), 3.13 (t, *J* = 7 Hz, 2H), 3.09 (t, *J* = 6.8 Hz, 1H), 1.65 (quint, *J* = 7.2 Hz, 2H), 1.56 (m, 2H), 1.43 (m, 2H), 1.39-1.16 (m, 18H), 0.89 (t, *J* = 6.8 Hz, 3H), 0.87 (t, *J* = 6.8 Hz, 3H). **^13^C NMR (101 MHz, CDCl_3_):** δ = 161.3 (d, *J*_C-F_ = 270 Hz), 150.6 (d, *J*_C-F_ = 13 Hz), 150.3, 145.2, 128.6, 127.8, 127.0 (d, *J*_C-F_ = 10 Hz), 110.8, 104.1 (d, *J*_C-F_ = 28 Hz), 101.7, 95.2, 44.0, 43.5, 31.9, 31.9, 29.6, 29.5, 29.44, 29.41, 29.3, 27.3, 27.2, 22.8, 22.7, 14.2, 14.2. One aromatic and one aliphatic carbon signal obscured or overlapping. NMR characterization was comparable to the previously reported results.

**General procedure for compounds 4a-e**

To a stirred solution of **2a-e** (0.82 mmol, 1 equiv.) in acetonitrile (10-15 mL), the corresponding amine (propylamine, butylamine, *tert*-butylamine, octylamine or methoxyethyl amine,) was added (2.5 mmol, 3 equiv.). The mixture was stirred at room temperature for 2 hours, while completion was monitored by TLC. After evaporation of the solvent, hot ethanol (15-20 mL) was added to the crude residue. An asphalt-like resulting material was collected by filtration and washed with hot water (30 mL), then recovered by dissolution in DCM, dried over anhydrous MgSO_4_ and filtered. After the solvent removed under reduced pressure and the solid dried under vacuum, the desired product was isolated as a brown asphalt-like solid.

**4a1**

Yield 98%.

**^1^H NMR** (**400 MHz, CDCl_3_):** δ = 9.27 (s, 1H), 9.13 (s, 1H), 8.22 (t, J = 4.8 Hz, 1H), 6.88 (d, *J* = 8.4 Hz, 1H), 6.02 (dd, *J* = 8.4 Hz, 2.4 Hz, 1H), 5.97 (d, *J* = 2.4 Hz, 1H), 5.70 (s, 1H), 3.81 (t, *J* = 5.2 Hz, 1H), 3.68 (br s, 1H), 3.15 (t, *J* = 7 Hz, 2H), 3.08 (q, *J* = 4.8 Hz, 2H), 2.99 (q, *J* = 6.4 Hz, 2H), 1.67-1.61 (m, 6H), 1.47 (sext, J = 7.4 Hz, 2H), 0.98 (t, *J* = 7.4 Hz, 3H), 0.93 (t, *J* = 7.4 Hz, 3H), 0.92 (t, *J* = 7.4 Hz, 3H). **^13^C NMR (101 MHz, CDCl_3_):** δ = 149.7, 149.2, 148.6, 145.3, 129.5, 128.8, 125.0, 124.5, 112.1, 101.6, 95.3, 93.1, 45.4, 44.8, 43.9, 31.8, 22.7, 21.7, 20.4, 14.0, 11.7, 11.5.

**4a2**

Yield 96%.

**^1^H NMR** (**400 MHz, CDCl_3_):** δ = 9.27 (s, 1H), 9.12 (s, 1H), 8.21 (t, *J* = 5 Hz, 1H), 6.88 (d, *J* = 8.4 Hz, 1H), 5.99 (dd, *J* = 8.4 Hz, 2.4 Hz, 1H), 5.97 (d, *J* = 2.4 Hz, 1H), 5.70 (s, 1H), 3.81 (t, *J* = 5.2 Hz, 1H), 3.68 (br s, 1H), 3.14 (t, *J* = 7 Hz, 2H), 3.07 (q, *J* = 6.7 Hz, 2H), 3.02 (q, *J* = 6.7 Hz, 2H), 1.68-1.52 (m, 6H), 1.46 (sext, *J* = 7.4 Hz, 2H), 1.34 (sext, *J* = 7.4 Hz, 2H), 0.98 (t, *J* = 7.4 Hz, 3H), 0.92 (t, *J* = 7.4 Hz, 3H), 0.89 (t, *J* = 7.4 Hz, 3H). **^13^C NMR (101 MHz, CDCl_3_):** δ = 149.7, 149.2, 148.6, 145.4, 129.6, 128.8, 125.0, 124.5, 112.1, 101.6, 95.3, 93.1, 45.4, 43.9, 42.9, 31.8, 30.4, 22.7, 20.4, 20.2, 14.0, 13.8, 11.7.

**4a3**

Yield 94%.

**^1^H NMR** (**400 MHz, CDCl_3_):** δ = 9.27 (s, 1H), 9.13 (s, 1H), 8.21 (t, *J* = 5 Hz, 1H), 6.87 (d, *J* = 8.4 Hz, 1H), 6.00 (dd, *J* = 8.2 Hz, 2.4 Hz, 1H), 5.96 (d, *J* = 2.4 Hz, 1H), 5.70 (s, 1H), 3.81 (t, *J* = 5 Hz, 1H), 3.67 (br s, 1H), 3.14 (t, *J* = 7 Hz, 2H), 3.07 (q, *J* = 6.3 Hz, 2H), 3.00 (q, *J* = 6.3 Hz, 2H), 1.98-1.51 (m, 6H), 1.46 (sext, *J* = 8 Hz, 2H), 1.31 (m, 10H), 0.98 (t, *J* = 7.2 Hz, 3H), 0.92 (t, *J* = 7.4 Hz, 3H), 0.88 (t, *J* = 7.4 Hz, 3H). **^13^C NMR (101 MHz, CDCl_3_):** δ = 149.7, 149.2, 148.6, 145.3, 129.6, 128.8, 125.0, 124.5, 112.1, 101.6, 95.3, 93.1, 45.4, 43.9, 43.2, 31.8, 31.8, 29.3, 29.3, 28.4, 27.1, 22.7, 22.7, 20.4, 14.2, 14.1, 11.7.

**4a4**

Yield 98%.

**^1^H NMR** (**400 MHz, CDCl_3_):** δ = 9.28 (s, 1H), 9.14 (br s, 1H), 8.37 (t, *J* = 4.6 Hz, 1H), 6.88 (d, *J* = 8 Hz, 1H), 6.01 (dd, *J* = 8.4 Hz, 2.4 Hz, 1H), 5.96 (d, *J* = 2.4 Hz, 1H), 5.72 (s, 1H), 3.81 (br s, 1H), 3.73 (br s, 1H), 3.55 (t, *J* = 5.4 Hz, 2H), 3.36 (s, 3H), 3.19 (t, *J* = 5 Hz, 2H), 3.14 (t, *J* = 7.2 Hz, 2H), 3.07 (t, *J* = 7 Hz, 2H), 1.68-1.52 (m, 4H), 1.46 (sext, *J* = 7.4 Hz, 2H), 0.98 (t, *J* = 7.4 Hz, 3H), 0.92 (t, *J* = 7.4 Hz, 3H). **^13^C NMR (101 MHz, CDCl_3_):** δ = 149.7, 149.3, 148.6, 145.4, 129.5, 128.8, 125.2, 124.5, 112.1, 101.6, 95.4, 93.2, 69.6, 59.1, 45.4, 43.8, 42.9, 31.8, 22.7, 20.4, 14.0, 11.7.

**4b1**

Yield 95%.

**^1^H NMR** (**400 MHz, CDCl_3_):** δ = 9.27 (s, 1H), 9.13 (s, 1H), 8.21 (t, *J* = 5 Hz, 1H), 6.87 (d, *J* = 8 Hz, 1H), 6.01 (dd, *J* = 8 Hz, 2.4 Hz, 1H), 5.97 (d, *J* = 2.4 Hz, 1H), 5.69 (s, 1H), 3.77 (br s, 1H), 3.68 (br s, 1H), 3.14 (t, *J* = 7 Hz, 2H), 3.08 (m, 2H), 2.98 (q, *J* = 6.8 Hz, 2H), 1.63 (m, 4H), 1.56 (m, 2H), 1.46 (sext, *J* = 7.2 Hz, 2H), 1.33-1.18 (br m, 10H), 0.98 (t, *J* = 7.2 Hz, 3H), 0.93 (t, *J* = 7.4 Hz, 3H), 0.86 (t, *J* = 6.8 Hz, 3H). **^13^C NMR (101 MHz, CDCl_3_):** δ = 149.7, 149.2, 148.6, 145.4, 129.5, 128.8, 125.0, 124.5, 112.1, 101.6, 95.3, 93.1, 44.8, 43.9, 43.6, 31.9, 31.8, 29.5, 29.4, 29.3, 27.2, 22.7, 21.7, 20.4, 14.2, 14.0, 11.5.

**4b2**

Yield 96%.

**^1^H NMR** (**400 MHz, CDCl_3_):** δ = 9.27 (s, 1H), 9.12 (s, 1H), 8.21 (t, *J* = 5.2 Hz, 1H), 6.87 (d, *J* = 8.4 Hz, 1H), 6.01 (dd, *J* = 8.4 Hz, 2.4 Hz, 1H), 5.96 (d, *J* = 2.4 Hz, 1H), 5.69 (s, 1H), 3.77 (t, *J* = 4.4 Hz, 1H), 3.68 (br s, 1H), 3.14 (t, *J* = 7.2 Hz, 2H), 3.08 (m, 2H), 3.01 (q, *J* = 6.4 Hz, 2H), 1.64 (quint, *J* = 7.2 Hz, 2H), 1.58 (m, 2H), 1.52 (m, 2H), 1.46 (sext, *J* = 7.4 Hz, 2H), 1.37-1.19 (br m, 12H), 0.98 (t, *J* = 7.2 Hz, 3H), 0.89 (t, *J* = 7.2 Hz, 3H), 0.86 (t, *J* = 7.2 Hz, 3H). **^13^C NMR (101 MHz, CDCl_3_):** δ = 149.7, 149.2, 148.6, 145.4, 129.5, 128.8, 125.0, 124.5, 112.1, 101.6, 95.3, 93.1, 43.8, 43.6, 42.9, 31.9, 31.8, 30.4, 29.5, 29.4, 29.3, 27.3, 22.7, 20.4, 20.2, 14.2, 14.0, 13.8.

**4b3**

Yield 88%.

**^1^H NMR** (**400 MHz, CDCl_3_):** δ = 9.27 (s, 1H), 9.13 (s, 1H), 8.21 (t, *J* = 5.2 Hz, 1H), 6.88 (d, *J* = 8.4 Hz, 1H), 6.01 (dd, *J* = 8.4 Hz, 2.4 Hz, 1H), 5.96 (d, *J* = 2.4 Hz, 1H), 5.69 (s, 1H), 3.77 (t, *J* = 5.2 Hz, 1H), 3.67 (br s, 1H), 3.14 (t, *J* = 7.2 Hz, 2H), 3.08 (m, 2H), 3.00 (q, *J* = 6.4 Hz, 2H), 1.64 (quint, *J* = 7.4 Hz, 2H), 1.56 (m, 4H), 1.44 (sext, *J* = 7.2 Hz, 2H), 1.34-1.18 (br m, 20H), 0.98 (t, *J* = 7.4 Hz, 3H), 0.89 (t, *J* = 7 Hz, 3H), 0.86 (t, *J* = 7 Hz, 3H). **^13^C NMR (101 MHz, CDCl_3_):** δ = 149.7, 149.2, 148.6, 145.39, 129.5, 128.8, 125.0, 124.5, 112.1, 101.6, 95.3, 93.1, 44.8, 43.9, 43.6, 31.9, 31.8, 29.5, 29.4, 29.3, 27.3, 22.7, 21.7, 20.4, 14.2, 14.1, 11.6. Five aliphatic carbon signals obscured or overlapping.

**4b4**

 Yield 82%.

**^1^H NMR** (**400 MHz, CDCl_3_):** δ = 9.27 (s, 1H), 9.14 (s, 1H), 8.37 (t, *J* = 4.6 Hz, 1H), 6.88 (d, *J* = 8.4 Hz, 1H), 6.01 (dd, *J* = 8.4 Hz, 2.4 Hz, 1H), 5.96 (d, *J* = 2.4 Hz, 1H), 5.71 (s, 1H), 3.78 (t, *J* = 4.8 Hz, 1H), 3.67 (br s, 1H), 3.55 (t, *J* = 5.4 Hz, 2H), 3.36 (s, 3H), 3.16 (m, 4H), 3.08 (q, *J* = 6.4 Hz, 2H), 1.64 (quint, *J* = 7.2 Hz, 2H), 1.56 (m, 2H), 1.46 (sext, *J* = 7.2 Hz, 2H), 1, 26 (br m, 10H), 0.98 (t, *J* = 7.4 Hz, 3H), 0.86 (t, *J* = 6.8 Hz, 3H). **^13^C NMR (101 MHz, CDCl_3_):** δ = 149.7, 149.3, 148.5, 145.4, 129.4, 128.8, 125.1, 124.5, 112.0, 101.6, 95.3, 93.1, 69.6, 59.1, 43.8, 43.6, 42.9, 31.9, 31.8, 29.5, 29.4, 29.3, 27.2, 22.7, 20.4, 14.2, 14.0.

**4c1**

Yield 99%.

**^1^H NMR** (**400 MHz, CDCl_3_):** δ = 9.27 (s, 1H), 9.12 (s, 1H), 8.21 (t, *J* = 5.2 Hz, 1H), 6.87 (d, *J* = 8.4 Hz, 1H), 6.01 (dd, *J* = 8 Hz, 2.4 Hz, 1H), 5.97 (d, *J* = 2 Hz, 1H), 5.70 (s, 1H), 3.81 (t, *J* = 5.2 Hz, 1H), 3.71 (br s, 1H), 3.11 (t, *J* = 7.2 Hz, 2H), 3.07 (m, 2H), 3.02 (m, 2H), 1.67 (sext, *J* = 7.2 Hz, 2H), 1.58 (m, 2H), 1.52 (m, 2H), 1.33 (sext, *J* = 7.4 Hz, 2H), 1.03 (t, *J* = 7.6 Hz, 3H), 0.92 (t, *J* = 7.4 Hz, 3H), 0.89 (t, *J* = 7.4 Hz, 3H). **^13^C NMR (101 MHz, CDCl_3_):** δ = 149.7, 149.2, 148.6, 145.3, 129.6, 128.8, 125.0, 124.5, 112.1, 101.6, 95.3, 93.1, 46.0, 45.4, 42.8, 30.3, 22.9, 22.7, 20.2, 13.7, 11.8, 11.7.

**4c2**

Yield 92%.

**^1^H NMR** (**400 MHz, CDCl_3_):** δ = 9.27 (s, 1H), 9.14 (s, 1H), 8.37 (t, *J* = 5 Hz, 1H), 6.87 (d, *J* = 8 Hz, 1H), 6.01 (dd, *J* = 8.4 Hz, 2.4 Hz, 1H), 5.97 (d, *J* = 2.4 Hz, 1H), 5.72 (s, 1H), 3.79 (br s, 2H), 3.55 (t, *J* = 5.2 Hz, 2H), 3.36 (s, 3H), 3.18 (q, *J* = 5.2 Hz, 2H), 3.11 (t, *J* = 7.2 Hz, 2H), 3.07 (t, *J* = 7.2 Hz, 2H), 1.68 (sext, *J* = 7.2 Hz, 2H), 1.59 (sext, *J* = 7.2 Hz, 2H), 1.03 (t, *J* = 7.4 Hz, 3H), 0.92 (t, *J* = 7.4 Hz, 3H). **^13^C NMR (101 MHz, CDCl_3_):** δ = 149.7, 149.4, 148.6, 145.5, 129.5, 128.9, 125.2, 124.6, 112.1, 101.6, 95.4, 93.2, 69.7, 59.1, 43.9, 43.3, 42.9, 31.8, 31.6, 20.4, 14.0.

**4d1**

Yield 99%.

**^1^H NMR** (**400 MHz, CDCl_3_):** δ = 9.27 (s, 1H), 9.12 (s, 1H), 8.21 (t, *J* = 5 Hz, 1H), 6.88 (d, *J* = 8.4 Hz, 1H), 6.00 (dd, *J* = 8.2 Hz, 2.6 Hz, 1H), 5.97 (d, *J* = 2 Hz, 1H), 5.69 (s, 1H), 3.78 (br s, 1H), 3.68 (br s, 1H), 3.15 (t, *J* = 7.2 Hz, 2H), 3.08 (m, 2H), 2.98 (q, *J* = 6.4 Hz, 2H), 1.63 (m, 4H), 1.56 (m, 2H), 1.46 (sext, *J* = 7.6 Hz, 2H), 1.35 (sext, *J* = 7.6 Hz, 2H), 0.98 (t, *J* = 7.2 Hz, 3H), 0.93 (t, *J* = 7.4 Hz, 3H), 0.91 (t, *J* = 7.2 Hz, 3H). **^13^C NMR (101 MHz, CDCl_3_):** δ = 149.7, 149.2, 148.6, 145.4, 129.5, 128.1, 125.0, 124.5, 112.1, 96.1, 94.0, 46.1, 44.8, 43.9, 43.3, 20.5, 20.4, 20.3, 19.3, 13.5, 13.4, 12.0. One aromatic carbon signal obscured or overlapping.

**4d2**

Yield 89%.

**^1^H NMR** (**400 MHz, CDCl_3_):** δ = 9.27 (s, 1H), 9.12 (s, 1H), 8.21 (t, *J* = 4.4 Hz, 1H), 6.88 (d, *J* = 8.4 Hz, 1H), 6.01 (dd, *J* = 8.4 Hz, 2.4 Hz, 1H), 5.97 (d, *J* = 2 Hz, 1H), 5.70 (s, 1H), 3.76 (br s, 2H), 3.14 (t, *J* = 6.8 Hz, 2H), 3.10 (t, *J* = 7 Hz, 2H), 3.02 (q, *J* = 6.8 Hz, 2H), 1.64 (quint, *J* = 7.2 Hz, 2H), 1.58 (m, 2H), 1.54 (m, 2H), 1.46 (sext, *J* = 7.4 Hz, 2H), 1.34 (quint, *J* = 7.4 Hz, 4H), 0.98 (t, *J* = 7.4 Hz, 3H), 0.91 (t, *J* = 7.2 Hz, 3H), 0.89 (t, *J* = 7.2 Hz, 3H). **^13^C NMR (101 MHz, CDCl_3_):** δ = 149.7, 149.2, 148.6, 145.4, 129.6, 128.8, 125.0, 124.5, 112.1, 101.6, 95.3, 93.1, 43.8, 43.3, 42.8, 31.8, 31.6, 30.3, 20.4, 20.3, 20.2, 14.09, 14.00, 13.7.

**4d3**

Yield 97%.

**^1^H NMR** (**400 MHz, CDCl_3_):** δ = 9.27 (s, 1H), 9.12 (s, 1H), 8.21 (t, *J* = 5 Hz, 1H), 6.87 (d, *J* = 8.4 Hz, 1H), 6.01 (dd, *J* = 8 Hz, 2.4 Hz, 1H), 5.97 (d, *J* = 2.4 Hz, 1H), 5.70 (s, 1H), 3.79 (br s, 1H), 3.70 (br s, 1H), 3.14 (t, *J* = 7.2 Hz, 2H), 3.09 (m, 2H), 3.01 (q, *J* = 6.2 Hz, 2H), 1.64 (quint, *J* = 7.2 Hz, 2H), 1.56 (m, 4H), 1.47 (quint, *J* = 7.2 Hz, 2H), 1.38-1.20 (br m, 10H), 0.98 (t, *J* = 7.2 Hz, 3H), 0.91 (t, *J* = 7.4 Hz, 3H), 0.88 (t, *J* = 7.4 Hz, 3H). **^13^C NMR (101 MHz, CDCl_3_):** δ = 149.6, 149.2, 148.5, 145.4, 129.5, 128.7, 124.9, 124.4, 112.1, 101.5, 95.3, 93.1, 43.8, 43.3, 43.2, 31.87, 31.85, 31.6, 29.3, 29.2, 28.3, 27.0, 22.7, 20.4, 20.3, 14.2, 14.0, 13.9.

**4e1**

Yield 89%

**^1^H NMR** (**400 MHz, CDCl_3_):** δ = 9.27 (s, 1H), 9.13 (s, 1H), 8.21 (t, *J* = 4.8 Hz, 1H), 6.88 (d, *J* = 8.4 Hz, 1H), 5.98 (dd, *J* = 8.3 Hz, 2.4 Hz, 1H), 5.96 (d, *J* = 2.4 Hz, 1H), 5.69 (s, 1H), 3.77 (br s, 1H), 3.69 (br s, 1H), 3.13 (t, *J* = 7.2 Hz, 2H), 3.08 (t, *J* = 6.7 Hz, 2H), 2.98 (q, *J* = 7 Hz, 2H), 1.65 (m, 4H), 1.56 (m, 2H), 1.42 (m 2H), 1.37-1.17 (br s, 18 H), 0.93 (t, *J* = 7.3 Hz, 3H), 0.89 (t, *J* = 6.7 Hz, 3H), 0.88 (t, *J* = 7.3 Hz, 3H). **^13^C NMR (101 MHz, CDCl_3_):** δ = 149.7, 149.2, 148.6, 145.3, 129.5, 128.8, 125.0, 124.5, 112.1, 101.6, 95.3, 93.1, 44.8, 44.2, 43.6, 31.97, 31.92, 29.7, 29.59, 29.53, 29.48, 29.41, 29.38, 27.35, 27.30, 22.81, 22.77, 21.70, 14.25, 14.22, 11.60.

**4e2**

Yield 77%.

**^1^H NMR** (**400 MHz, CDCl_3_):** δ = 9.27 (s, 1H), 9.01 (s, 1H), 8.40 (br s, 1H), 6.88 (d, *J* = 8 Hz, 1H), 6.01 (dd, *J* = 8.4 Hz, 2.4 Hz, 1H), 5.96 (d, *J* = 2.4 Hz, 1H), 5.91 (s, 1H), 3.81 (br s, 1H), 3.71 (br s, 1H), 3.13 (t, *J* = 7.2 Hz, 2H), 3.05 (t, *J* = 7.2 Hz, 2H), 1.63 (quint, *J* = 7.2 Hz, 2H), 1.52 (quint, *J* = 7.2 Hz, 2H), 1.41 (m 2H), 1.35-1.26 (br m, 12H), 1.25 (s, 9H), 1.22 (br m, 6H), 0.89 (t, *J* = 6.8 Hz, 3H), 0.86 (t, *J* = 7 Hz, 3H). **^13^C NMR (101 MHz, CDCl_3_):** δ = 149.8, 148.5, 147.5, 145.5, 129.6, 129.1, 125.6, 124.1, 112.2, 101.68, 95.8, 95.2, 52.1, 44.2, 43.6, 31.98, 31.91, 29.67, 29.61, 29.5, 29.46, 29.43, 29.36, 29.1, 27.3, 27.2, 22.8, 22.7, 14.2, 14.2. NMR characterization is comparable to the previously reported results.

**4e3**

 Yield 99%.

**^1^H NMR** (**400 MHz, CDCl_3_):** δ = 9.25 (s, 1H), 9.12 (s, 1H), 8.21 (t, *J* = 4.92 Hz, 1H), 6.88 (d, *J* = 7.6 Hz, 1H), 6.00 (d, *J* = 8.4 Hz, 1H), 5.96 (s, 1H), 5.69 (s, 1H), 3.79 (t, *J* = 4.6 Hz, 1H), 3.68 (br s, 1H), 3.13 (t, *J* = 7.1 Hz, 2H), 3.08 (t, *J* = 6.4 Hz, 2H), 2.99 (q, *J* = 6.6 Hz, 2H), 1.65 (quint, *J* = 7.2 Hz, 2H), 1.56 (m, 2H), 1.42 (m, 2H), 1.35-1.26 (br m, 30H), 0.86 (m, 9H). NMR characterization is comparable to the previously reported results.

**General procedure for triamino-phenazinium 5a-e**

A solution of compound **4a-e** (0.6 mmol, 1 equiv.) in MeOH (20 mL) was hydrogenated (20 bars) in the presence of Pd/C (5 wt. %, 5% mol) and HCl (12M, 0.3 mL) for 6 hours. Then the mixture was stirred under air for 16 h. Pd/C was removed by filtration through a Celite® plug which was rinsed multiple times with MeOH and DCM. After removal of the solvent under reduced pressure, the resulting solid was taken up with DCM (50 mL), and extracted with brine (2 x 50 mL), the organic layer was then dried over MgSO_4_, filtered and evaporated under vacuum to afford crude black solid which was then purified over alumina oxide gel chromatography column (neutral, Brucker activity I, DCM/ MeOH, 95/5, R_f_ = 0.5) and led to a deep purple solid.

**5a1**

Yield 34%.

**^1^H NMR** (**400 MHz, CD_3_OD):** δ = 7.64 (d, *J* = 9.2 Hz, 1H), 7.14 (d, *J* = 9.2 Hz, 1H), 6.93 (s, 1H), 6.65 (s, 1H), 6.49 (br s, 1H), 4.50 (t, *J* = 8 Hz, 2H), 3.29 (m, 2H), 3.19 (t, *J* = 7.2 Hz, 2H), 1.95 (sext, *J* = 8 Hz, 2H), 1.80 (sext, *J* = 7.4 Hz, 2H), 1.73 (quint, *J* = 7.2 Hz, 2H), 1.52 (sext, *J* = 7.6 Hz, 2H), 1.21 (t, *J* = 7.4 Hz, 3H), 1.10 (t, *J* = 7.4 Hz, 3H), 1.03 (t, *J* = 7.2 Hz, 3H). **^13^C NMR (101 MHz, CD_3_OD):** δ = 154.5, 152.0, 140.4, 139.6, 136.5, 133.8, 132.4, 132.0, 121.2, 103.9, 93.6, 90.4, 46.7, 44.0, 31.7, 22.5, 21.4, 21.0, 14.2, 12.1, 11.4, 11.3. **HRMS (ESI+)** calculated for [C^+^]: 366.2652 (C_22_H_32_N_5_^+^) found: 366.2648.

**5a2**

Yield 62%.

**^1^H NMR** (**400 MHz, CD_3_OD):** δ = 7.75 (d, *J* = 9.2 Hz, 1H), 7.21 (dd, *J* = 9.2 Hz, 2 Hz, 1H), 6.98 (s, 1H), 6.78 (s, 1H), 6.58 (br s, 1H), 4.57 (t, *J* = 8 Hz, 2H), 3.34 (traces of MeOH), 3.35 (t, *J* = 7.2 Hz, 2H), 3.29 (t, *J* = 7.6 Hz, 2H), 1.98 (sext, *J* = 7.6 Hz, 2H), 1.80-1.64 (m, 4H), 1.51 (quint, *J* = 7.2 Hz, 4H), 1.20 (t, *J* = 7.4 Hz, 3H), 1.02 (t, *J* = 7.4 Hz, 3H), 1.01 (t, *J* = 7.4 Hz, 3H). **^13^C NMR (101 MHz, CD_3_OD):** δ = 154.5, 152.1, 140.4, 139.7, 136.5, 133.9, 132.5, 132.0, 121.0, 104.0, 93.6, 90.5, 44.7, 44.0, 43.8, 31.8, 31.5, 21.6, 21.4, 21.0, 14.2 (2C), 11.4. **HRMS (ESI+)** calculated for [C^+^]: 380.2809 (C_23_H_34_N_5_^+^) found: 380.2808.

**5a3**

Yield 45%.

**^1^H NMR** (**400 MHz, CD_3_OD):** δ = 7.74 (d, *J* = 9.6 Hz, 1H), 7.21 (dd, *J* = 9.2 Hz, 2 Hz, 1H), 6.98 (s, 1H), 6.77 (s, 1H), 6.58 (br s, 1H), 5.48 (traces of DCM), 4.57 (t, *J* = 8.2 Hz, 2H), 3.34 (traces of MeOH), 3.30 (t, *J* = 7.4 Hz, 2H), 3.28 (t, *J* = 7.4 Hz, 2H), 1.99 (sext, *J* = 8 Hz, 2H), 1.82-1.66 (m, 4H), 1.49 (quint, *J* = 8 Hz, 4H), 1.41-1.28 (m, 8H), 1.22 (t, *J* = 7.4 Hz, 3H), 1.03 (t, *J* = 7.4 Hz, 3H), 0.91 (t, *J* = 7.4 Hz, 3H). **^13^C NMR (101 MHz, CD_3_OD):** δ = 154.6, 152.2, 140.5, 139.9, 136.9, 136.7, 134.0, 132.6, 132.2, 104.1, 93.7, 90.5, 44.9, 44.0, 33.0, 31.8, 30.6, 30.4, 29.4, 28.4, 23.7, 21.4, 21.0, 14.4, 14.2, 14.1, 11.4. **HRMS (ESI+)** calculated for [C^+^]: 436.3435 (C_27_H_42_N_5_^+^) found: 436.3433.

**5a4**

Yield 57%.

**^1^H NMR** (**400 MHz, CD_3_OD):** δ = 7.78 (d, *J* = 9.4 Hz, 1H), 7.24 (d, *J* = 9.4 Hz, 1H), 7.01 (s, 1H), 6.89 (s, 1H), 6.62 (br s, 1H), 4.60 (t, *J* = 8 Hz, 2H), 3.75 (t, *J* = 5.2 Hz, 2H), 3.51 (t, *J* = 5.2 Hz, 2H), 3.44 (s, 3H), 3.34 (t, *J* = 7.2 Hz, 2H), 2.01 (sext, *J* = 7.6 Hz, 2H), 1.73 (quint, *J* = 7 Hz, 2H), 1.53 (sext, *J* = 7 Hz, 2H), 1.21 (t, *J* = 7.4 Hz, 3H), 1.02 (t, *J* = 7.4 Hz, 3H). **^13^C NMR (101 MHz, CD_3_OD):** δ = 154.8, 152.2, 140.5, 139.7, 136.7, 134.1, 132.7, 132.2, 104.7, 93.8, 93.7, 71.2, 59.1, 50.2, 44.7, 44.0, 31.8, 21.4, 21.1, 21.0, 14.2, 11.4. **HRMS (ESI+)** calculated for [C^+^]: 382.2601 (C_22_H_35_N_5_O^+^) found: 382.2601.

**5b1**

Yield 43%.

**^1^H NMR** (**400 MHz, CD_3_OD):** δ = 7.61 (d, *J* = 9.2 Hz, 1H), 7.13 (d, *J* = 9.2 Hz, 2 Hz, 1H), 6.88 (s, 1H), 6.60 (s, 1H), 6.39 (br s, 1H), 4.46 (t, *J* = 7.6 Hz, 2H), 3.24 (t, J = 7.2 Hz, 2H), 3.18 (m, 2H), 1.88 (quint, *J* = 6.8 Hz, 2H), 1.80 (sext, *J* = 7.2 Hz, 2H), 1.73 (quint, *J* = 7.4 Hz, 2H), 1.61 (m, 2H), 1.53 (m, 4H) 1.43-1.32 (br m, 6H), 1.10 (t, *J* = 7.4 Hz, 3H), 1.03 (t, *J* = 7.2 Hz, 3H), 0.90 (t, *J* = 6.8 Hz, 3H). **^13^C NMR (101 MHz, CD_3_OD):** δ = 154.4, 152.0, 140.4, 139.7, 136.5, 133.7, 132.5, 131.9, 121.2, 104.0, 93.5, 90.3, 46.8, 44.1, 32.9, 31.8, 30.4, 27.9, 27.5, 23.7, 22.6, 21.4, 14.4, 14.3, 12.1. Two aliphatic carbon signals obscured or overlapping. **HRMS (ESI+)** calculated for [C^+^]: 436.3435 (C_27_H_42_N_5_^+^) found: 436.3432.

**5b2**

Yield 55%.

**^1^H NMR** (**400 MHz, CD_3_OD):** δ = 7.73 (d, *J* = 9.2 Hz, 1H), 7.19 (dd, *J* = 9.2 Hz, 2 Hz, 1H), 6.95 (s, 1H), 6.76 (s, 1H), 6.52 (br s, 1H), 4.59 (t, *J* = 4.2 Hz, 2H), 3.33 (m, 2H), 3.27 (m, 2H), 1.96 (quint, *J* = 7.8 Hz, 2H), 1.76 (m, 4H), 1.64 (quint, *J* = 7.6 Hz, 2H), 1.52 (m, 6H), 1.45-1.32 (br m, 6H), 1.04 (t, *J* = 7.4 Hz, 3H), 1.01 (t, *J* = 7.4 Hz, 3H), 0.90 (t, *J* = 6.8 Hz, 3H). **^13^C NMR (101 MHz, CD_3_OD):** δ = 154.6, 152.2, 140.6, 139.9, 136.7, 134.0, 132.6, 132.2, 121.4, 112.9, 104.1, 93.6, 44.7, 44.0, 32.9, 31.8, 31.5, 30.4, 27.9, 27.6, 23.7, 21.5, 21.4, 14.4, 14.2. Three aliphatic carbon signals obscured or overlapping. **HRMS (ESI+)** calculated for [C^+^]: 450.3591 (C_28_H_44_N_5_^+^) found: 450.3588.

**5b3**

Yield 34%.

**^1^H NMR** (**400 MHz, CD_3_OD):** δ = 7.73 (d, *J* = 9.2 Hz, 1H), 7.20 (dd, *J* = 9 Hz, 2 Hz, 1H), 6.96 (s, 1H), 6.76 (s, 1H), 6.52 (br s, 1H), 4.57 (t, *J* = 8 Hz, 2H), 3.32 (m, 2H), 3.24 (m, 2H), 1.94 (quint, *J* = 7.6 Hz, 2H), 1.77 (m, 4H), 1.64 (quint, *J* = 7.4 Hz, 2H), 1.49 (m, 6H), 1.45-1.32 (br, 14H), 1.03 (t, *J* = 7.4 Hz, 3H), 0.91 (m, 6H). **^13^C NMR (101 MHz, CD_3_OD):** δ = 154.6, 152.2, 140.6, 139.9, 136.7, 134.0, 132.6, 132.2, 104.1, 93.6, 45.0, 44.0, 33.0, 32.9, 31.8, 30.6, 30.4, 29.4, 28.4, 27.9, 27.6, 23.7, 21.4, 14.4, 14.2. Two aromatic and five aliphatic carbon signals obscured or overlapping. H**RMS (ESI+)** calculated for [C^+^]: 506.4217 (C_32_H_52_N_5_^+^) found: 506.4218.

**5b4**

Yield 47%.

**^1^H NMR** (**400 MHz, CD_3_OD):** δ = 7.56 (d, *J* = 9 Hz, 1H), 7.11 (dd, *J* = 9.2 Hz, 1.87 Hz, 1H), 6.84 (s, 1H), 6.60 (s, 1H), 6.34 (br s, 1H), 4.44 (t, *J* = 8 Hz, 2H), 3.76 (t, *J* = 5.3 Hz, 2H), 3.44 (s, 3H), 3.41 (t, *J* = 5.41 Hz, 2H), 3.27 (t, *J* = 7.1 Hz, 2H), 1.85-1.32 (mm, 16H), 1.04 (t, *J* = 7.2 Hz, 3H), 0.92 (t, *J* = 7.1 Hz, 3H). **^13^C NMR (101 MHz, CD_3_OD):** δ = 154.3, 151.8, 140.1, 139.1, 136.2, 133.4, 132.3, 131.5, 120.9, 104.3, 93.5, 90.5, 71.2, 59.2, 48.8 (overlapped with solvent peak, confirmed with ^13^C DEPT 135 analysis)44.7, 44.1, 32.9, 31.7, 30.5, 30.4, 27.8, 27.4, 23.7, 21.5, 14.5, 14.3. H**RMS (ESI+) c**alculated for [(C^+^A^-^)C^+^]: 939.6462 (C_27_H_42_ClN_5_O^+^); found: 939.6448.

**5c1**

Yield 50%.

**^1^H NMR** (**400 MHz, CD_3_OD):** δ = 7.74 (d, *J* = 9.2 Hz, 1H), 7.21 (dd, *J* = 9.2 Hz, 2 Hz, 1H), 6.97 (s, 1H), 6.76 (s, 1H), 6.57 (br s, 1H), 4.55 (t, *J* = 8 Hz, 2H), 3.33 (m, 2H), 3.27 (m, 2H), 1.97 (quint, *J* = 7.6 Hz, 2H), 1.76 (sext, *J* = 7.2 Hz, 4H), 1.56 (sext, *J* = 7.6 Hz, 2H), 1.20 (t, *J* = 7.4 Hz, 3H), 1.07 (t, *J* = 7.4 Hz, 3H), 1.02 (t, *J* = 7.4 Hz, 3H). **^13^C NMR (101 MHz, CD_3_OD):** δ = 154.7, 152.2, 140.6, 139.9, 136.7, 134.1, 132.6, 132.3, 107.5, 104.1, 93.7, 90.6, 50.2, 46.0, 44.6, 31.5, 22.9, 21.5, 21.0, 14.2, 11.9, 11.4. **HRMS (ESI+)** calculated for [C^+^]: 366.2652 (C_22_H_32_N_5_^+^) found: 366.2650.

**5c2**

Yield 59%.

**^1^H NMR** (**400 MHz, CD_3_OD):** δ = 7.59 (d, *J* = 9.2 Hz, 1H), 7.13 (dd, *J* = 9.2 Hz, 1.6 Hz, 1H), 6.87 (s, 1H), 6.63 (s, 1H), 6.43 (br s, 1H), 4.45 (t, *J* = 8 Hz, 2H), 3.75 (t, *J* = 5.2 Hz, 2H), 3.43 (s, 3H), 3.40 (t, *J* = 5.2 Hz, 2H), 3.27 (t, *J* = 6.8 Hz, 2H), 1.92 (sext, *J* = 7.6 Hz, 2H), 1.77 (sext, *J* = 7.2 Hz, 2H), 1.19 (t, *J* = 7.3 Hz, 3H), 1.08 (t, *J* = 7.3 Hz, 3H). **^13^C NMR (101 MHz, CD_3_OD):** δ = 154.7, 152.0, 140.3, 139.5, 136.5, 133.9, 132.6, 131.9, 121.0, 104.6, 93.7, 90.4, 71.2, 59.1,50.2, 46.0, 44.7, 22.9, 21.0, 12.0, 11.4. **HRMS (ESI+)** calculated for [C^+^]: 368.2445 (C_21_H_30_N_5_O ^+^) found: 368.2442.

**d1**

Yield 50%.

**^1^H NMR** (**400 MHz, CD_3_OD):** δ = 7.69 (d, *J* = 9.4 Hz, 1H), 7.18 (dd, *J* = 9.2 Hz, *J* = 1.9 Hz, 1H), 6.94 (s, 1H), 6.71 (s, 1H), 6.50 (br s, 1H), 4.56 (t, *J* = 8 Hz, 2H), 3.33 (m, 2H), 3.23 (t, *J* = 7.2 Hz, 2H), 1.90 (m, 2H), 1.81 (quint, *J* = 7.4 Hz, 2H), 1.70 (m, 4H), 1.50 (quint, *J* = 7.4 Hz, 2H), 1.11 (m, 6H), 1.03 (t, *J* = 7.4 Hz, 3H).**^13^C NMR (101 MHz, CD_3_OD):** δ = 154.2, 151.9, 140.2, 139.4, 136.2, 133.5, 132.3, 131.6, 120.9, 103.8, 93.4, 90.2, 46.8, 44.1, 31.7, 30.4, 29.4, 22.6, 21.4, 21.1, 14.3, 14.1, 12.2.**HRMS (ESI+)** calculated for [C^+^]: 380.2809 (C_23_H_34_N_5_^+^) found: 380.2805.

**5d2**

Yield 56%.

**^1^H NMR** (**400 MHz, CD_3_OD):** δ = 7.72 (d, *J* = 9.6 Hz, 1H), 7.20 (dd, *J* = 9.4 Hz, 2 Hz, 1H), 6.96 (s, 1H), 6.74 (s, 1H), 6.53 (br s, 1H), 4.59 (t, *J* = 8.1 Hz, 2H), 3.34 (m, 2H), 3.26 (t, *J* = 7.15 Hz, 2H), 1.96 (quint, *J* = 7.4 Hz, 2H), 1.73 (m, 6H), 1.53 (quint, *J* = 7.4 Hz, 4H), 1.11 (t, *J* = 7.4 Hz, 3H), 1.01 (m, 6H). **^13^C NMR (101 MHz, CD_3_OD):** δ = 154.6, 152.2, 140.6, 139.9, 136.7, 134.0, 132.6, 132.2, 121.2, 104.1, 93.6, 90.4, 44.7, 44.0, 31.8, 31.5, 29.5, 21.5, 21.4, 21.1, 14.2, 14.2, 14.1. One aliphatic carbon signal obscured or overlapping. **HRMS (ESI+)** calculated for [C^+^]: 394.2965 (C_24_H_36_N_5_^+^) found: 394.2962.

**5d3**

Yield 69%.

**^1^H NMR** (**400 MHz, CD_3_OD):** δ = 7.71 (d, *J* = 9.2 Hz, 1H), 7.19 (dd, *J* = 9.2 Hz, 2 Hz, 1H), 6.95 (s, 1H), 6.71 (s, 1H), 6.52 (br s, 1H), 4.56 (t, *J* = 8 Hz, 2H), 3.30 (m, 2H), 3.24 (t, *J* = 7.2 Hz, 2H), 1.92 (quint, *J* = 7.2 Hz, 2H), 1.78-1.64 (m, 6H), 1.51 (quint, *J* = 7.6 Hz, 4H), 1.33 (br m, 8H), 1.11 (t, *J* = 7.4 Hz, 3H), 1.02 (t, *J* = 7.4 Hz, 3H), 0.90 (t, *J* = 6.8 Hz, 3H). **^13^C NMR (101 MHz, CD_3_OD):** δ = 154.6, 152.1, 140.5, 139.8, 136.7, 133.9, 132.6, 132.1, 121.2, 104.1, 93.6, 45.0, 44.0, 33.0, 31.8, 30.6, 30.4, 29.5, 29.4, 28.4, 23.7, 21.4, 21.1, 14.4, 14.2, 14.1. One aromatic and one aliphatic carbon signals obscured or overlapping. **HRMS (ESI+)** calculated for [C^+^]: 450.3591 (C_28_H_44_N_5_^+^) found: 450.3589.

**5e1**

Yield 55%.

**^1^H NMR** (**400 MHz, CD_3_OD):** δ = 7.67 (d, *J* = 9.3 Hz, 1H), 7.15 (dd, *J* = 9.2 Hz, 2 Hz, 1H), 6.92 (s, 1H), 6.70 (s, 1H), 6.46 (br s, 1H), 4.53 (t, *J* = 8 Hz, 2H), 3.25 (m, 2H), 3.22 (t, *J* = 7.6 Hz, 2H), 1.92 (quint, *J* = 7.6 Hz, 2H), 1.81 (sext, *J* = 7.4 Hz, 2H), 1.74 (quint, *J* = 7.2 Hz, 2H), 1.63 (quint, *J* = 7.4 Hz, 2H), 1.46 (m, 4H), 1.43-1.30 (br m, 14H), 1.10 (t, *J* = 7.4 Hz, 3H), 0.90 (m, 6H). **^13^C NMR (101 MHz, CD_3_OD):** δ = 154.4, 152.1, 140.5, 139.8, 136.6, 133.9, 132.6, 132.0, 121.1, 104.1, 93.6, 90.3, 46.8, 44.4, 33.0, 33.0, 30.6, 30.5, 29.7, 28.4, 27.9, 27.5, 23.7, 22.6, 14.4, 14.4, 12.1. Four aliphatic carbon signals obscured or overlapping. **HRMS (ESI+)** calculated for [C^+^]: 492.4061 (C_31_H_50_N_5_^+^) found: 492.4059.

**5e2**

Yield 79%.

**^1^H NMR** (**400 MHz, CD_3_OD):** δ = 7.81 (d, *J* = 9.6 Hz, 1H), 7.25 (d, *J* = 2 Hz, 1H), 7.23 (s, 1H), 7.05 (s, 1H), 6.60 (br s, 1H), 4.62 (t, *J* = 8 Hz, 2H), 3.37 (t, *J* = 7.2 Hz, 2H), 1.97 (quint, *J* = 7.4 Hz, 2H), 1.76 (quint, *J* = 7.4 Hz, 2H), 1.65 (m, 2H), 1.57 (s, 9H), 1.49 (m, 2H), 1.49 (quint, *J* = 8 Hz, 2H), 1.45-1.37 (m, 14H), 0.90 (m, 6H). **^13^C NMR (101 MHz, CD_3_OD):** δ = 154.9, 153.5, 142.7, 139.1, 136.7, 134.4, 132.5, 132.3, 94.3, 44.3, 33.0, 32.9, 30.6, 30.5, 30.4, 29.7, 29.0, 28.3, 27.9, 27.6, 23.7, 14.4, 14.4. Three aromatic carbon and four aliphatic carbon signals obscured or overlapping. NMR characterization and HRMS is comparable to the previously reported results. **HRMS (ESI+)** calculated for [C^+^]: 506.4217 (C_32_H_52_N_5_^+^) found: 506.4215.

**5e3**

Yield 61%.

**^1^H NMR** (**400 MHz, CD_3_OD):** δ = 7.72 (d, *J* = 9.2 Hz, 1H), 7.20 (dd, *J* = 9.2 Hz, 1.6 Hz, 1H), 6.96 (s, 1H), 6.75 (s, 1H), 6.51 (br s, 1H), 4.57 (t, *J* = 8 Hz, 2H), 3.28 (m, 2H), 3.24 (t, *J* = 7.2 Hz, 2H), 1.95 (quint, *J* = 7.6 Hz, 2H), 1.77 (m, 4H), 1.64 (quint, *J* = 7.6 Hz, 2H), 1.49 (m, 6H), 1.44-1.26 (br, 22H), 0.91 (m, 9H). **^13^C NMR (101 MHz, CD_3_OD):** δ = 154.5, 152.1, 140.5, 139.9, 136.4, 134.0, 132.6, 132.1, 121.4, 104.1, 93.6, 90.2, 45.0, 44.4, 33.0, 30.6, 30.5, 29.7, 29.4, 28.5, 28.4, 27.9, 27.5, 23.7, 14.4. Eleven aliphatic carbon signals obscured or overlapping. NMR characterization and HRMS is comparable to the previously reported results. **HRMS (ESI+)** calculated for [C^+^]: 562.4843 (C_36_H_60_N_5_^+^) found: 562.4843.

1. NMR SPECTRA
2.

Figure S 1. ^1^H NMR (400 MHz, CDCl_3_) of compound **1a.**

Figure S 2. ^13^C NMR (101 MHz, CDCl_3_) of compound **1a.**

Figure S 3 ^1^H NMR (400 MHz, CDCl_3_) of **1b.**

Figure S 4 ^13^C NMR (101 MHz, CDCl_3_) of **1b.**

Figure S 5 ^1^H NMR (400 MHz, CDCl_3_) of **2a.**

Figure S 6 ^13^C NMR (101 MHz, CDCl_3_) of **2a.**

Figure S 7 ^1^H NMR (400 MHz, CDCl_3_) of **2b.**

Figure S 8 ^13^C NMR (101 MHz, CDCl_3_) of **2b.**

Figure S 9 ^1^H NMR (400 MHz, CDCl_3_) of **2c.**

Figure S 10 ^13^C NMR (101 MHz, CDCl_3_) of **2c.**

Figure S 11 ^1^H NMR (400 MHz, CDCl_3_) of **2d.**

Figure S 12 ^13^C NMR (101 MHz, CDCl_3_) of **2d.**

Figure S 13 ^1^H NMR (400 MHz, CDCl_3_) of **2e.**

Figure S 14 ^13^C NMR (101 MHz, CDCl_3_) of **2e.**

Figure S 15 ^1^H NMR (400 MHz, CDCl_3_) of **3a.**

Figure S 16 ^13^C NMR (101 MHz, CDCl_3_) of **3a.**

Figure S 17 ^1^H NMR (400 MHz, CDCl_3_) of **3b.**

Figure S 18 ^13^C NMR (101 MHz, CDCl_3_) of **3b.**

Figure S 19 ^1^H NMR (400 MHz, CDCl_3_) of **3c.**

Figure S 20 ^13^C NMR (101 MHz, CDCl_3_) of **3c.**

Figure S 21 ^1^H NMR (400 MHz, CDCl_3_) of **3d.**

Figure S 22 ^13^C NMR (101 MHz, CDCl_3_) of **3d.**

Figure S 23 ^1^H NMR (400 MHz, CDCl_3_) of **3e.**

Figure S 24 ^13^C NMR (101 MHz, CDCl_3_) of **3e.**

Figure S 25 ^1^H NMR (400 MHz, CDCl_3_) of **4a1.**

Figure S 26 ^13^C NMR (101 MHz, CDCl_3_) of **4a1.**

Figure S 27 ^1^H NMR (400 MHz, CDCl_3_) of **4a2.**

Figure S 28 ^13^C NMR (101 MHz, CDCl_3_) of **4a2.**

Figure S 29 ^1^H NMR (400 MHz, CDCl_3_) of **4a3.**

Figure S 30 ^13^C NMR (101 MHz, CDCl_3_) of **4a3.**

Figure S 31 ^1^H NMR (400 MHz, CDCl_3_) of **4a4.**

Figure S 32 ^13^C NMR (101 MHz, CDCl_3_) of **4a4.**

Figure S 33 ^1^H NMR (400 MHz, CDCl_3_) of **4b1.**

Figure S 34 ^13^C NMR (101 MHz, CDCl_3_) of **4b1.**

Figure S 35 ^1^H NMR (400 MHz, CDCl_3_) of **4b2.**

Figure S 36 ^13^C NMR (101 MHz, CDCl_3_) of **4b2.**

Figure S 37 ^1^H NMR (400 MHz, CDCl_3_) of **4b3.**

Figure S 38 ^13^C NMR (101 MHz, CDCl_3_) of **4b3.**

Figure S 39 ^1^H NMR (400 MHz, CDCl_3_) of **4b4.**

Figure S 40 ^13^C NMR (101 MHz, CDCl_3_) of **4b4.**

Figure S 41 ^1^H NMR (400 MHz, CDCl_3_) of **4c1.**

Figure S 42 ^13^C NMR (101 MHz, CDCl_3_) of **4c1.**

Figure S 43 ^1^H NMR (400 MHz, CDCl_3_) of **4c2.**

Figure S 44 ^13^C NMR (101 MHz, CDCl_3_) of **4c2.**

Figure S 45 ^1^H NMR (400 MHz, CDCl_3_) of **4d1.**

Figure S 46 ^13^C NMR (101 MHz, CDCl_3_) of **4d1.**

Figure S 47 ^1^H NMR (400 MHz, CDCl_3_) of **4d2.**

Figure S 48 ^13^C NMR (101 MHz, CDCl_3_) of **4d2.**

Figure S 49 ^1^H NMR (400 MHz, CDCl_3_) of **4d3.**

Figure S 50 ^13^C NMR (101 MHz, CDCl_3_) of **4d3.**

Figure S 51 ^1^H NMR (400 MHz, CDCl_3_) of **4e1.**

Figure S 52 ^13^C NMR (101 MHz, CDCl_3_) of **4e1.**

Figure S 53 ^1^H NMR (400 MHz, CDCl_3_) of **4e2.**

Figure S 54 ^13^C NMR (101 MHz, CDCl_3_) of **4e2.**

Figure S 55 ^1^H NMR (400 MHz, CDCl_3_) of **4e3.**

Figure S 56 ^1^H NMR (400 MHz, CD_3_OD) of **5a1.**

Figure S 57 ^13^C NMR (101 MHz, CD_3_OD) of **5a1.**

Figure S 58 ^1^H NMR (400 MHz, CD_3_OD) of **5a2.**

Figure S 59 ^13^C NMR (101 MHz, CD_3_OD) of **5a2.**

Figure S 60 ^1^H NMR (400 MHz, CD_3_OD) of **5a3.**

Figure S 61 ^13^C NMR (101 MHz, CD_3_OD) of **5a3.**

Figure S 62 ^1^H NMR (400 MHz, CD_3_OD) of **5a4.**

Figure S 63 ^13^C NMR (101 MHz, CD_3_OD) of **5a4.**

Figure S 64 ^1^H NMR (400 MHz, CD_3_OD) of **5b1.**

Figure S 65 ^13^C NMR (101 MHz, CD_3_OD) of **5b1.**

Figure S 66 ^1^H NMR (400 MHz, CD_3_OD) of **5b2.**

Figure S 67 ^13^C NMR (101 MHz, CD_3_OD) of **5b2.**

Figure S 68 ^1^H NMR (400 MHz, CD_3_OD) of **5b3.**

Figure S 69 ^13^C NMR (101 MHz, CD_3_OD) of **5b3.**

Figure S 70 ^1^H NMR (400 MHz, CD_3_OD) of **5b4.**

Figure S 71 ^13^C NMR (101 MHz, CD_3_OD) of **5b4.**

Figure S 72 ^13^C DEPT135 NMR (101 MHz, CD_3_OD) of **5b4.**

Figure S 73 ^1^H NMR (400 MHz, CD_3_OD) of **5c1.**

Figure S 74 ^13^C NMR (101 MHz, CD_3_OD) of **5c1.**

Figure S 75 ^1^H NMR (400 MHz, CD_3_OD) of **5c2.**

Figure S 76 ^13^C NMR (101 MHz, CD_3_OD) of **5c2.**

Figure S 77 ^1^H NMR (400 MHz, CD_3_OD) of **5d1.**

Figure S 78 ^13^C NMR (101 MHz, CD_3_OD) of **5d1.**

Figure S 79 ^1^H NMR (400 MHz, CD_3_OD) of **5d2.**

Figure S 80 ^13^C NMR (101 MHz, CD_3_OD) of **5d2.**

Figure S 81 ^1^H NMR (400 MHz, CD_3_OD) of **5d3.**

Figure S 82 ^13^C NMR (101 MHz, CD_3_OD) of **5d3.**

Figure S 83 ^1^H NMR (400 MHz, CD_3_OD) of **5e1.**

Figure S 84 ^13^C NMR (101 MHz, CD_3_OD) of **5e1.**

Figure S 85 ^1^H NMR (400 MHz, CD_3_OD) of **5e2.**

Figure S 86 ^13^C NMR (101 MHz, CD_3_OD) of **5e2.**

Figure S 87 ^1^H NMR (400 MHz, CD_3_OD) of **5e3.**

Figure S 88 ^13^C NMR (101 MHz, CD_3_OD) of **5e3.**

1. MASS SPECTROMETRY


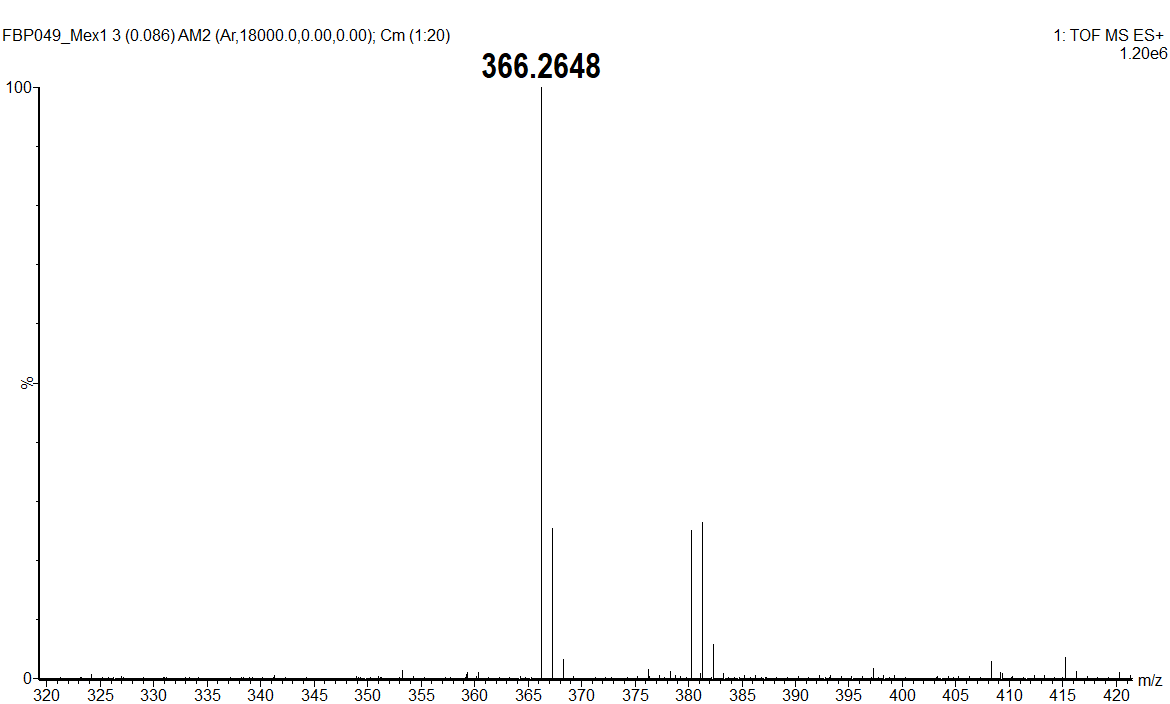


Figure S 89 HRMS spectrum of **5a1.**


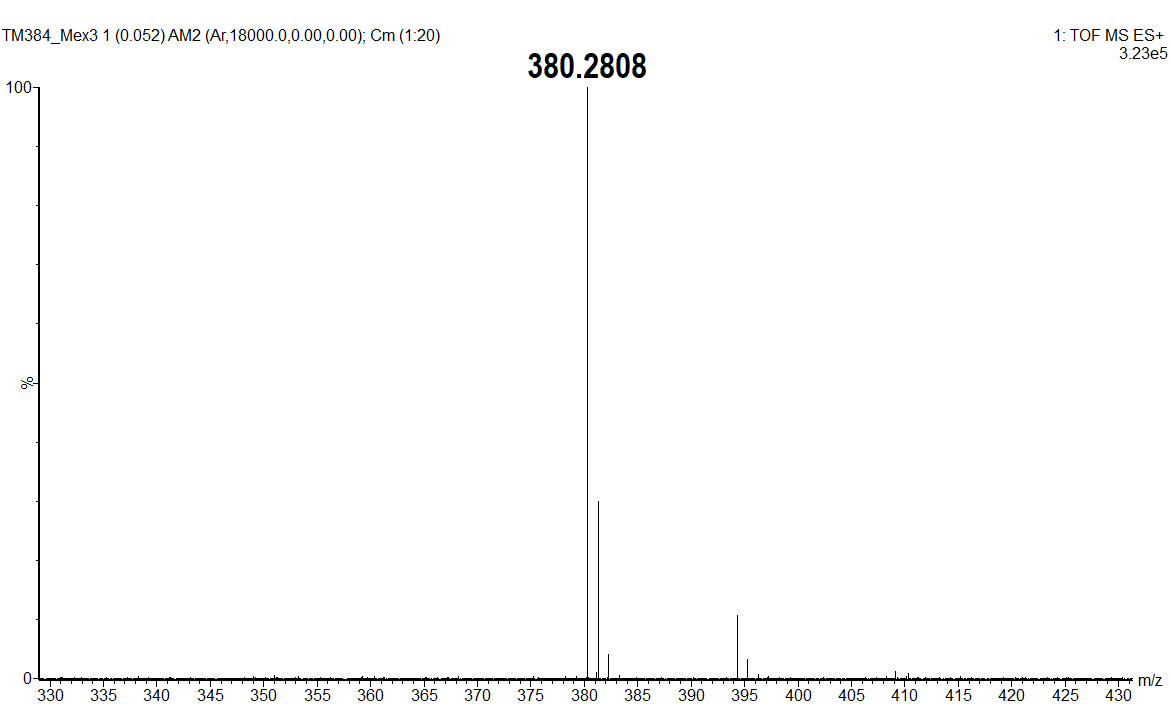


Figure S 90 HRMS spectrum of **5a2.**

Figure S 91. HRMS spectrum of **5a3.**

Figure S 92 HRMS spectrum of **5a4.**

Figure S 93 HRMS spectrum of **5b1.**

Figure S 94 HRMS spectrum of **5b2.**

Figure S 95 HRMS spectrum of **5b3.**


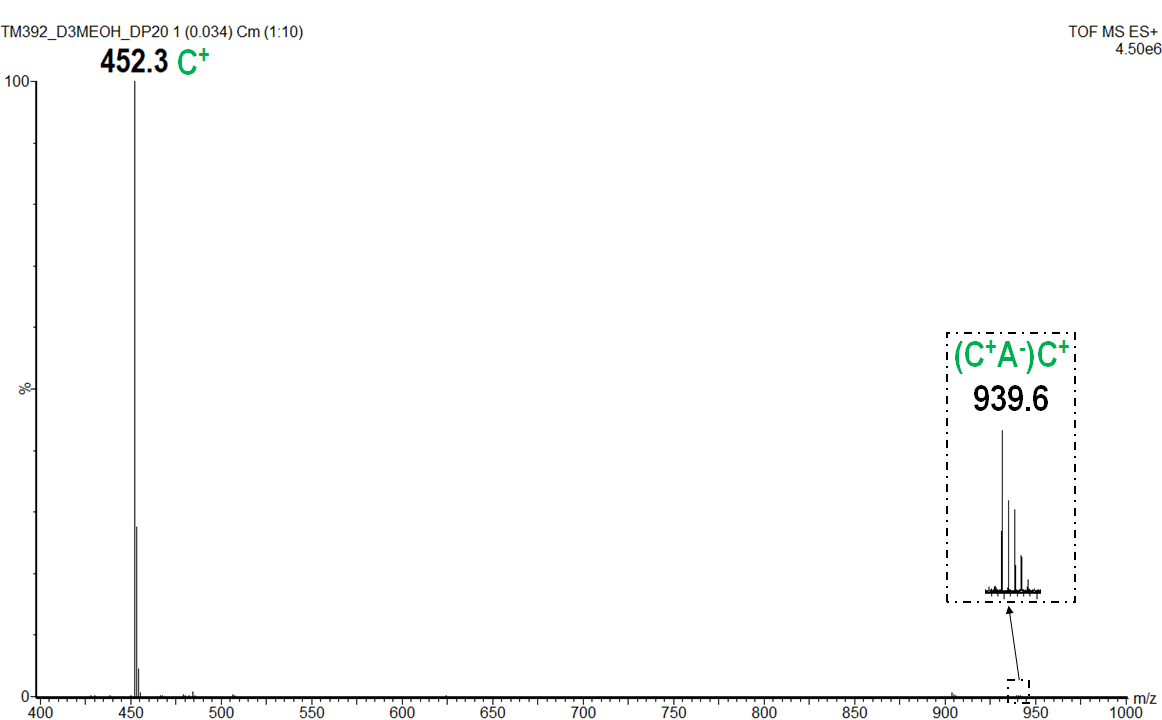


Figure S 96 LRMS spectrum of **5b4** detected as an agglomerate type (C^+^A^-^)C^+^


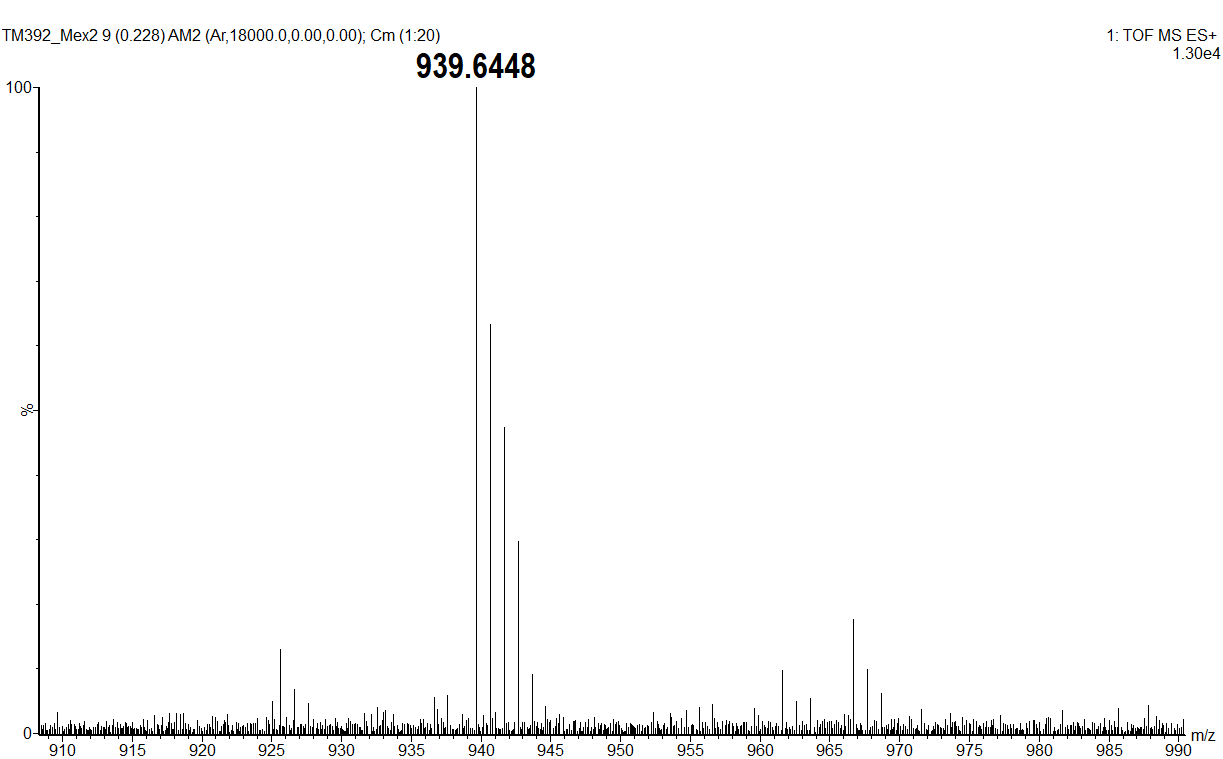


Figure S 97 HRMS spectrum of **5b4** detected as an agglomerate type (C^+^A^-^)C^+^. Figure S 98 HRMS spectrum of **5c1.**


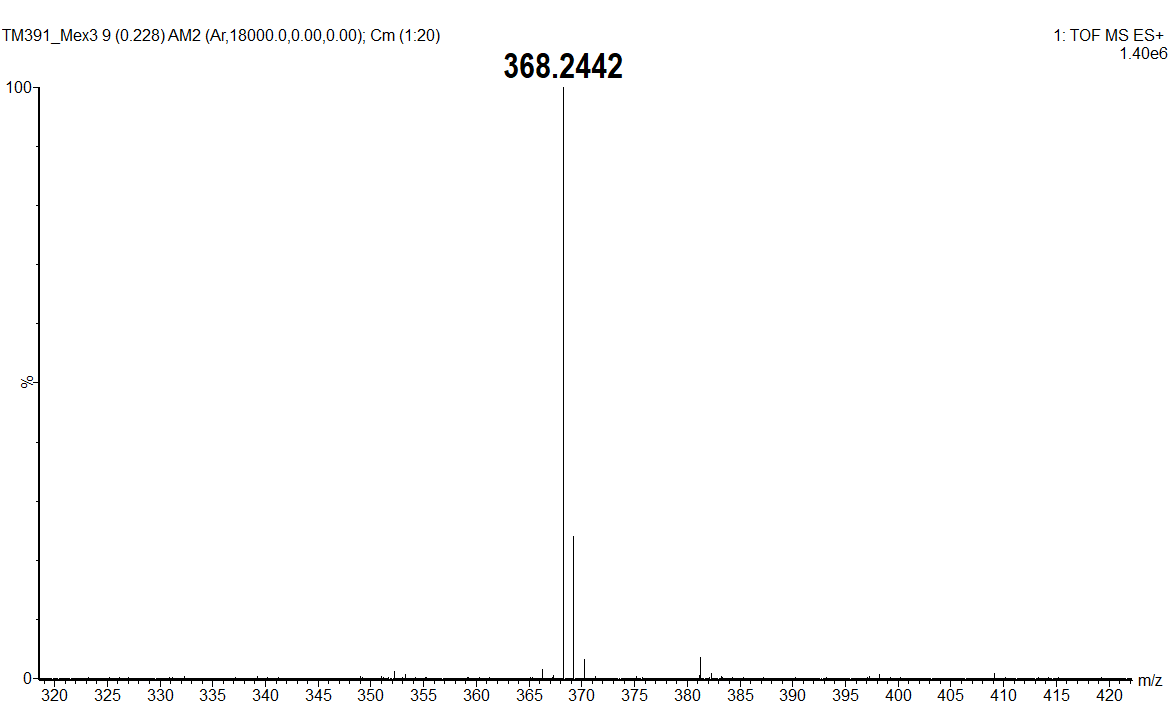


Figure S 99 HRMS spectrum of **5c2.**


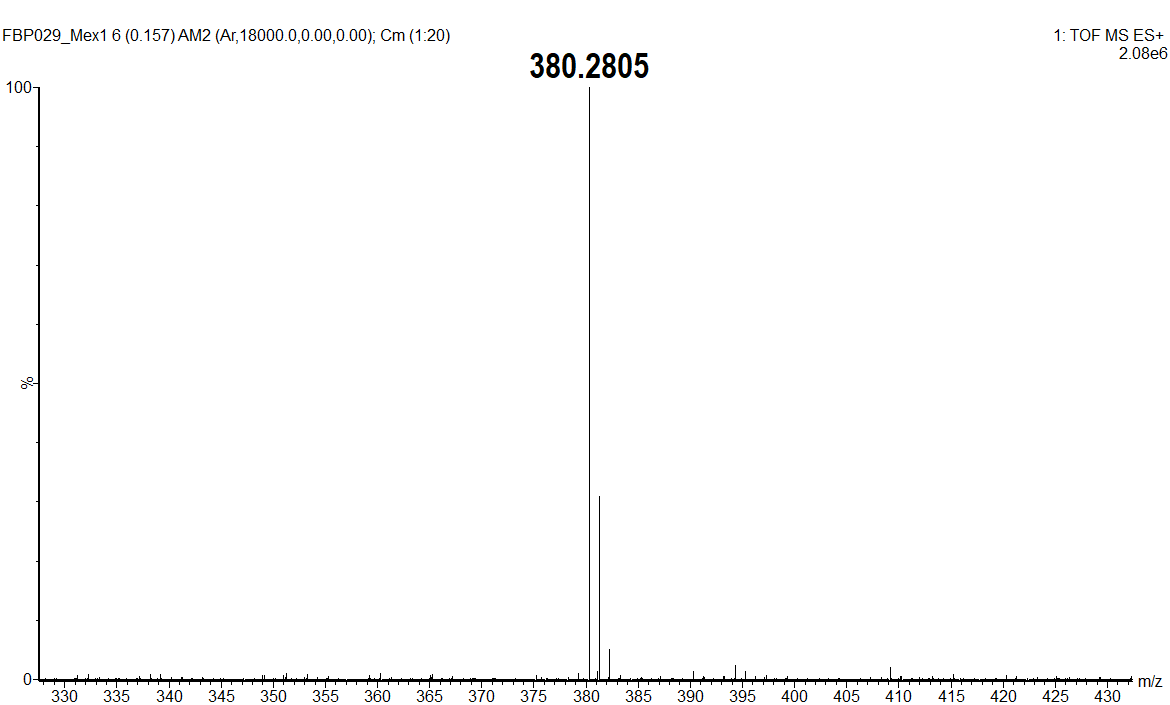


Figure S 100 HRMS spectrum of **5d1.**


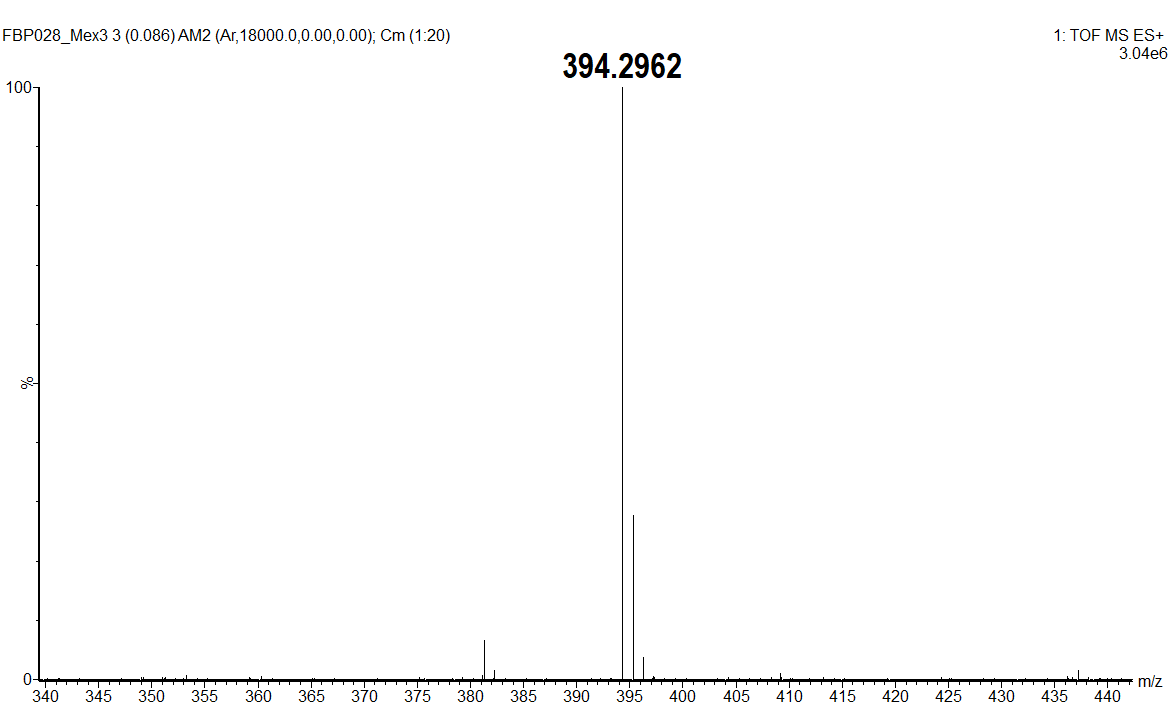


Figure S 101 HRMS spectrum of **5d2.**


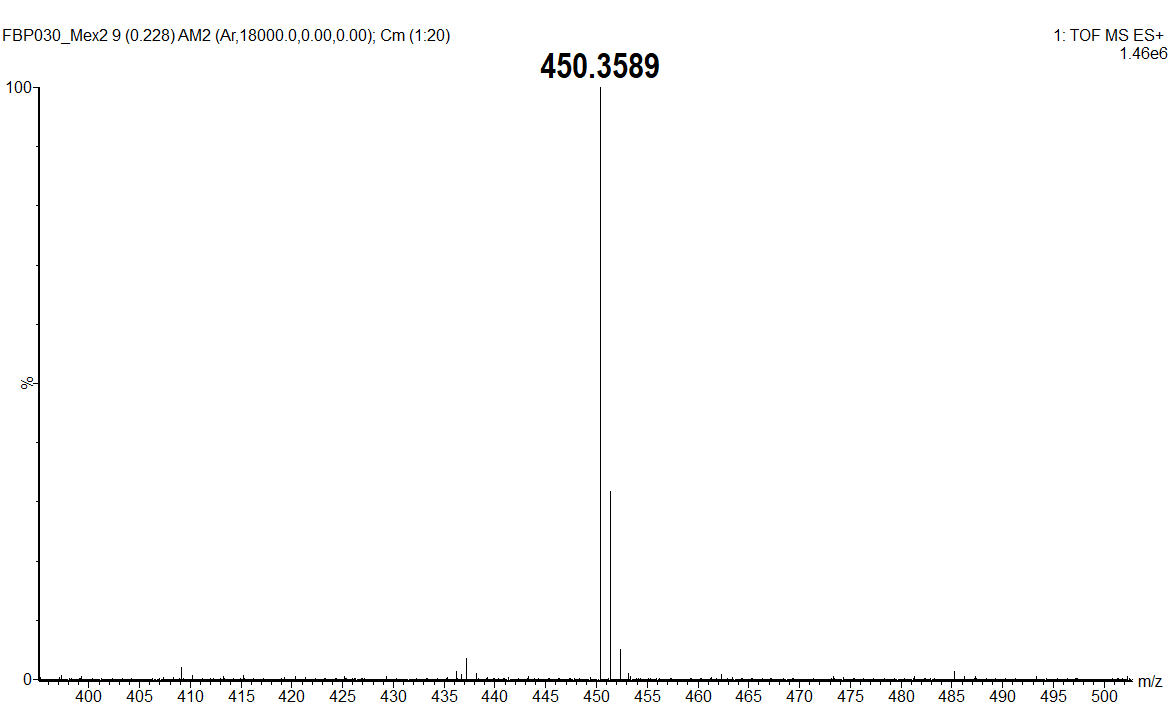


Figure S 102 HRMS spectrum of **5d3.**


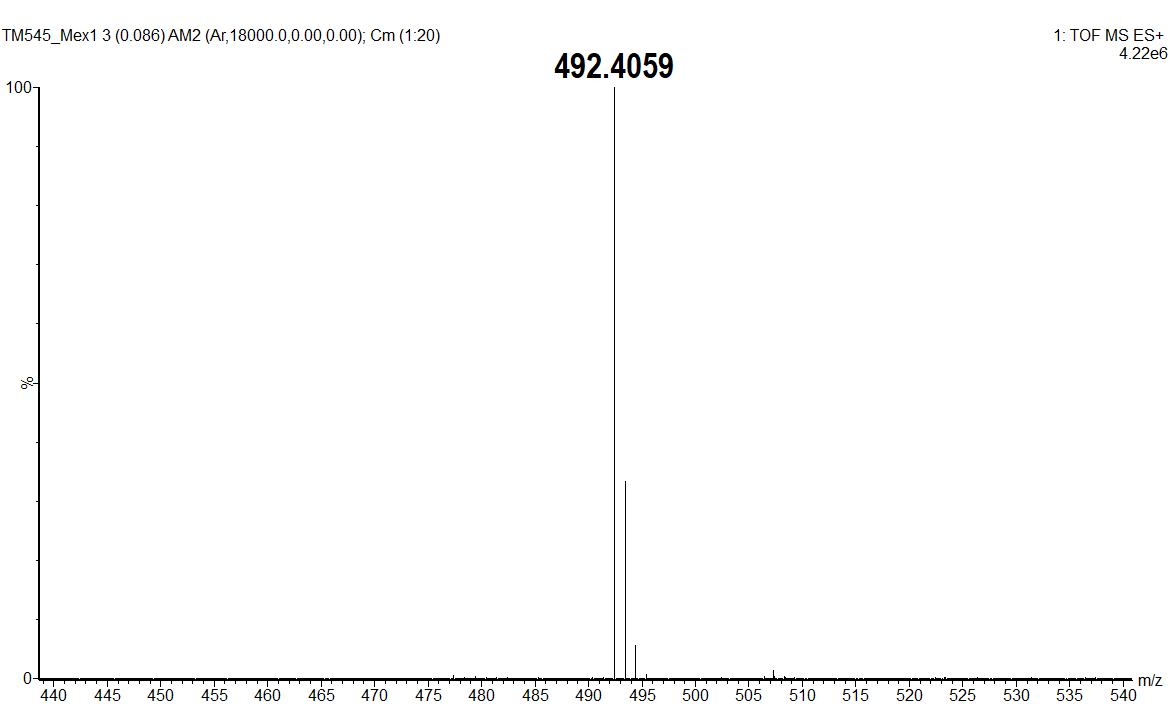


Figure S 103 HRMS spectrum of **5e1.**


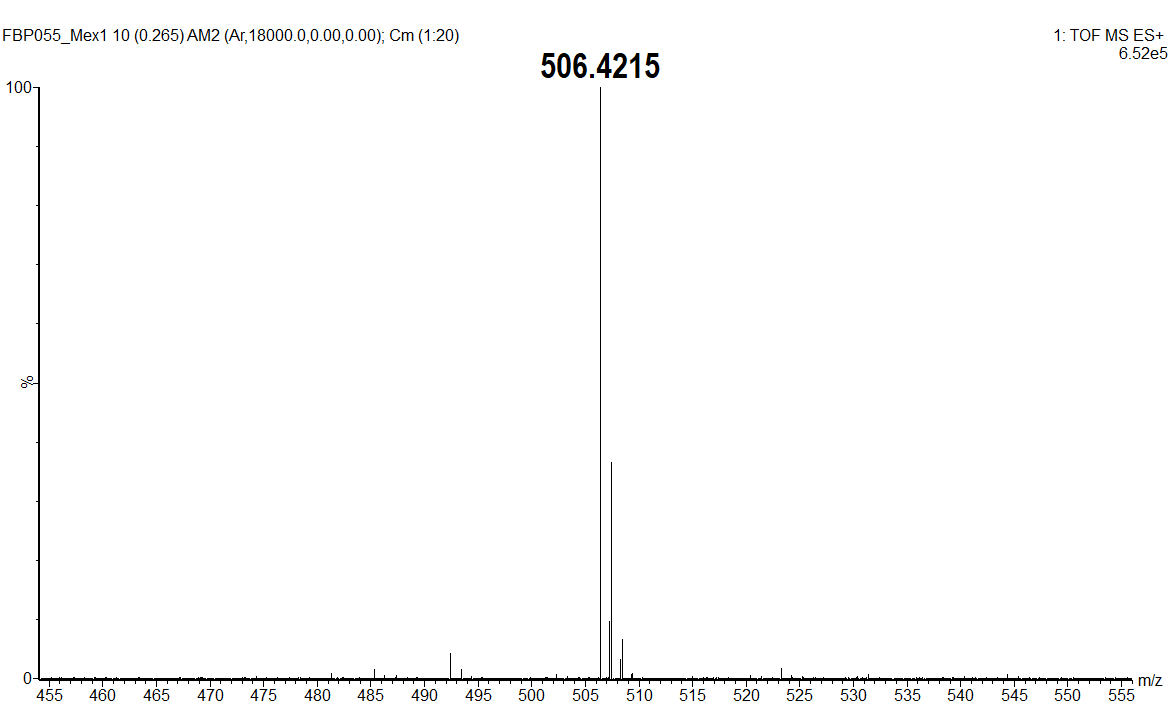


Figure S 104 HRMS spectrum of **5e2.**


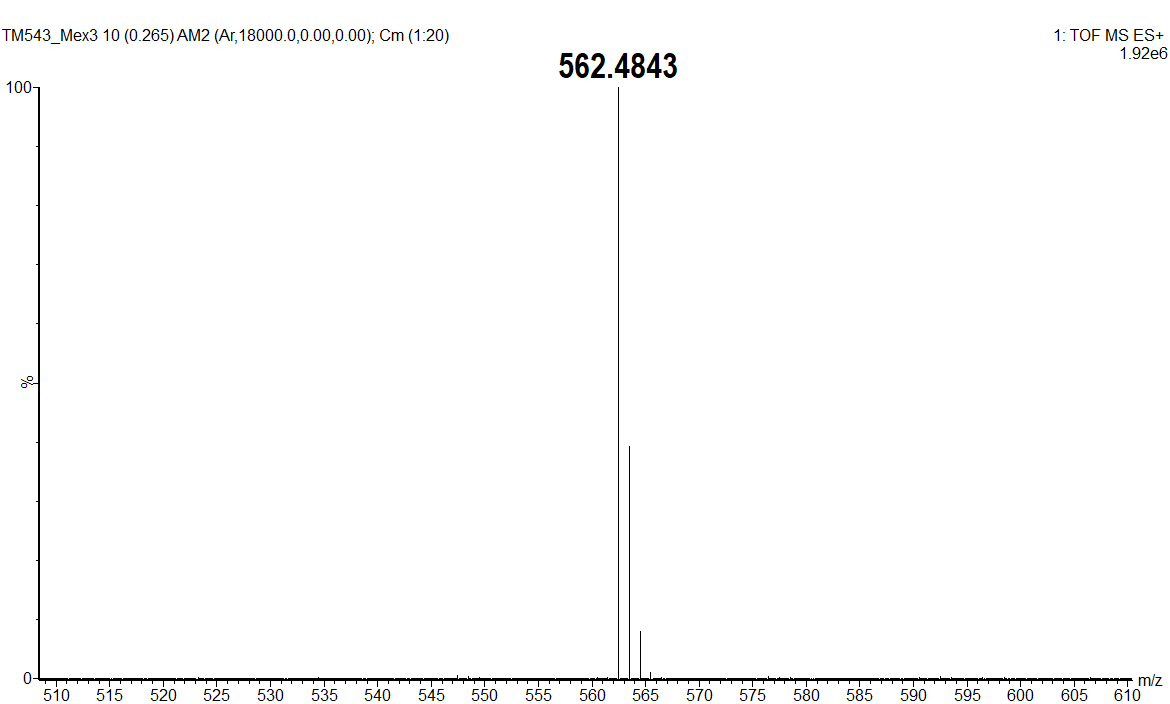


Figure S 105 HRMS spectrum of **5e3.**

2. **Strains-related supplementary information**

**Table S1: List of clinical strains with their characteristics**

| **Strains** | **Source** | **Species** | **Susceptibility profile** | | | **QRDR mutations** | | **Efflux detection method** | | **Reference** |
| --- | --- | --- | --- | --- | --- | --- | --- | --- | --- | --- |
|  |  |  | **CIP** | **MXF** | **MDR/**  **MRSA status** | **grlA** | **gyrA** | **5a4** | **EtBrCW** |  |
| SA-1199 | As mentioned in Table 2 | *S. aureus* | S | S | MSSA/non-MDR | WT | WT | Negative | Negative | ^[2]^ |
| SA-1199B |  | *S. aureus* | R | S | NorA MDR | A116E | - | Positive | Positive |  |
| SA-K1758 |  | *S. aureus* | S | S | MSSA/non-MDR | ND | ND | Negative | Negative |  |
| SA-K1712 |  | *S. aureus* | S | S | MSSA/non-MDR | ND | ND | Negative | ND | ^[3]^ |
| SA-K1748 |  | *S. aureus* | R | R | MDR mutant of SA-K1712 | S80F | - | Positive | Positive |  |
| SA-K2068 |  | *S. aureus* | S | R | MDR mutant of NCTC 8325-4 | Non-QRDR mutations | | Positive | Positive |  |
| ATCC25923 | Reference strain | *S. aureus* | I | S | MSSA/non-MDR | WT | WT | Negative | Negative | ^[4]^ |
| ATCC25923_EtBr | Couto’s Lab | *S. aureus* | I | ND | - | WT | WT | Positive | Positive |  |
| ATCC12228 | Reference strain | *S. epidermidis* | I | S | MSSE/non-MDR | WT | WT | Negative | Negative | ^[5]^ |
| ATCC12228_EtBr | Couto’s Lab | *S. epidermidis* | R | R | - | WT | WT | Positive | Positive |  |
| SM 1 | Human | *S. aureus* | R | R | MRSA/MDR | S80Y/E84G | S84L | Positive | Positive | ^[6]^ |
| SM 10 | Human | *S. aureus* | R | R | MRSA/MDR | S80Y/E84G | S84L | Positive | Positive |  |
| SM 14 | Human | *S. aureus* | R | R | MRSA/MDR | S80Y/E84G | S84L | Positive | Positive |  |
| SM 17 | Human | *S. aureus* | R | R | MRSA/MDR | S80Y/E84G | S84L | Positive | Positive |  |
| SM 25 | Human | *S. aureus* | R | R | MRSA/MDR | S80Y/E84G | S84L | Positive | Positive |  |
| SM 27 | Human | *S. aureus* | R | R | MRSA/MDR | S80Y/E84G | S84L | Positive | Positive |  |
| SM 43 | Human | *S. aureus* | R | R | MRSA/MDR | S80Y/E84G | S84L | Positive | Positive |  |
| SM 46 | Human | *S. aureus* | R | R | MRSA/MDR | S80Y/E84G | S84L | Positive | Positive |  |
| SM 47 | Human | *S. aureus* | R | R | MRSA/MDR | S80Y/E84G | S84L | Positive | Positive |  |
| SM 48 | Human | *S. aureus* | R | R | MRSA/MDR | S80Y/E84G | S84L | Positive | Positive |  |
| SM 50 | Human | *S. aureus* | R | R | MRSA/MDR | S80F/E84K | S84L | Negative | Negative |  |
| SM 52 | Human | *S. aureus* | R | R | MRSA/MDR | S80Y | S84L | Negative | Negative |  |
| SM 2 | Human | *S. aureus* | R | R | MRSA/MDR | S80F/E84K | S84L | Negative | Negative^*^ |  |
| SM 3 | Human | *S. aureus* | R | R | MRSA/MDR | S80F/E84G | S84L | Negative | Negative^*^ |  |
| SM 4 | Human | *S. aureus* | R | R | MRSA/MDR | S80F | S84L | Negative | Negative |  |
| SM 5 | Human | *S. aureus* | R | R | MRSA/MDR | S80F/E84G | S84L | Negative | Negative |  |
| SM 6 | Human | *S. aureus* | R | R | MRSA/MDR | S80F | E88K | Negative | Negative |  |
| SM 7 | Human | *S. aureus* | R | R | MRSA/MDR | S80F | S84L | Negative | Negative |  |
| SM 8 | Human | *S. aureus* | R | R | MRSA/MDR | S80F | E88K | Negative | Negative^*^ |  |
| SM 12 | Human | *S. aureus* | R | R | MRSA/MDR | S80F | S84L | Negative | Negative |  |
| SM 16 | Human | *S. aureus* | R | R | MSSA/non-MDR | S80F | E88K | Negative | Negative |  |
| SM 22 | Human | *S. aureus* | R | R | MRSA/MDR | S80Y/E84G | S84L | Positive | Negative^*^ |  |
| SM 34 | Human | *S. aureus* | R | R | MSSA/MDR | S80F/E84K | S84L | Negative | Negative |  |
| SM 36 | Human | *S. aureus* | R | R | MRSA/MDR | S80F | S84L | Negative | Negative |  |
| SM 39-L | Human | *S. aureus* | I | S | MRSA/MDR | WT | WT | Positive | Positive | This study |
| SM 40 | Human | *S. aureus* | R | R | MRSA/MDR | S80F | S84L | Positive | Positive | ^[6]^ |
| BIOS-H1 | Human | *S. aureus* | I | S | MSSA/non-MDR | ND | ND | Negative | Negative | ^[7]^ |
| BIOS-H2 | Human | *S. aureus* | I | S | MSSA/non-MDR | ND | ND | Negative | Negative |  |
| BIOS-H3 | Human | *S. aureus* | I | S | MSSA/non-MDR | ND | ND | Negative | Negative |  |
| BIOS-H4 | Human | *S. aureus* | R | R | MSSA/MDR | ND | ND | Negative | Negative^*^ |  |
| BIOS-H5 | Human | *S. aureus* | I | S | MRSA/non-MDR | ND | ND | Negative | Negative |  |
| BIOS-H6 | Human | *S. aureus* | I | S | MRSA/non-MDR | ND | ND | Negative | Negative |  |
| BIOS-H7 | Human | *S. aureus* | R | R | MSSA/MDR | S80Y/E84G | S84L | Positive | Negative |  |
| BIOS-H8 | Human | *S. aureus* | R | R | MRSA/MDR | S80Y/E84G | S84L | Positive | Negative |  |
| BIOS-H9 | Human | *S. aureus* | R | R | MSSA/MDR | ND | ND | Negative | Negative |  |
| BIOS-H10 | Human | *S. aureus* | R | R | MRSA/MDR | S80Y/E84G | S84L | Positive | Negative^*^ |  |
| BIOS-H11 | Human | *S. aureus* | R | R | MSSA/MDR | S80Y/E84G | S84L | Positive | Negative |  |
| BIOS-H12 | Human | *S. aureus* | I | S | MSSA/non-MDR | ND | ND | Negative | Negative |  |
| BIOS-H13 | Human | *S. aureus* | I | S | MRSA/non-MDR | ND | ND | Negative | Negative |  |
| BIOS-H14 | Human | *S. aureus* | R | R | MRSA/MDR | S80Y | S84L | Positive | Negative |  |
| BIOS-H16 | Human | *S. aureus* | R | R | MRSA/MDR | ND | ND | Negative | Negative |  |
| BIOS-H17 | Human | *S. aureus* | I | S | MSSA/non-MDR | ND | ND | Negative | Negative |  |
| BIOS-H19 | Human | *S. aureus* | R | R | MRSA/MDR | S80Y/E84G | ND | Positive | Negative |  |
| BIOS-H20 | Human | *S. aureus* | I | S | MSSA/non-MDR | ND | ND | Negative | Negative |  |
| BIOS-H21 | Human | *S. aureus* | I | S | MSSA/non-MDR | ND | ND | Negative | Negative |  |
| BIOS-H22 | Human | *S. aureus* | I | S | MSSA/non-MDR | ND | ND | Negative | Negative |  |
| BIOS-H23 | Human | *S. aureus* | R | R | MRSA/MDR | S80Y/E84G | S84L | Positive | Negative |  |
| BIOS-H24 | Human | *S. aureus* | R | R | MSSA/non-MDR | ND | ND | Negative | Negative |  |
| BIOS-H25 | Human | *S. aureus* | I | S | MSSA/non-MDR | ND | ND | Negative | Negative |  |
| BIOS-H28 | Human | *S. aureus* | I | S | MSSA/non-MDR | ND | ND | Negative | Negative |  |
| BIOS-H29 | Human | *S. aureus* | I | S | MSSA/non-MDR | WT | WT | Negative | Negative |  |
| BIOS-H30 | Human | *S. aureus* | I | S | MSSA/non-MDR | ND | S84L | Negative | Negative |  |
| BIOS-H31 | Human | *S. aureus* | R | R | MRSA/MDR | S80F | ND | Positive | Negative |  |
| BIOS-H32 | Human | *S. aureus* | I | S | MSSA/non-MDR | WT | WT | Negative | Negative |  |
| BIOS-H33 | Human | *S. aureus* | R | R | MRSA/MDR | S80Y/E84G | S84L | Positive | Negative |  |
| BIOS-H34 | Human | *S. aureus* | R | R | MRSA/MDR | ND | ND | Negative | Negative |  |
| BIOS-H35 | Human | *S. aureus* | R | R | MRSA/MDR | ND | ND | Negative | Negative |  |
| BIOS-V4 | Dog | *S. aureus* | R | R | MRSA/MDR | ND | ND | Negative | Negative | ^[8]^ |
| BIOS-V5 | Dog | *S. aureus* | R | R | MRSA/non-MDR | ND | ND | Negative | Negative |  |
| BIOS-V21 | Cat | *S. aureus* | I | S | MSSA/non-MDR | ND | ND | Negative | Negative |  |
| BIOS-V31 | Dog | *S. aureus* | I | S | MSSA/non-MDR | ND | ND | Negative | Negative |  |
| BIOS-V60 | Horse | *S. aureus* | I | S | MSSA/non-MDR | ND | ND | Negative | Negative |  |
| BIOS-V61 | Dog | *S. aureus* | I | S | MSSA/non-MDR | ND | ND | Negative | Negative |  |
| BIOS-V62 | Cat | *S. aureus* | I | S | MSSA/non-MDR | ND | ND | Negative | Negative |  |
| BIOS-V70 | Dog | *S. aureus* | R | R | MRSA/non-MDR | S80Y/S81P | S84L | Negative | Negative |  |
| BIOS-V74 | Dog | *S. aureus* | I | S | MSSA/non-MDR | ND | ND | Negative | Negative |  |
| BIOS-V75 | Dog | *S. aureus* | I | S | MSSA/non-MDR | ND | ND | Negative | Negative |  |
| BIOS-V85 | Dog | *S. aureus* | I | S | MSSA/non-MDR | ND | ND | Negative | Negative |  |
| BIOS-V118 | Dog | *S. aureus* | I | S | MSSA/non-MDR | ND | ND | Negative | Negative |  |
| BIOS-V128 | Cat | *S. aureus* | I | S | MSSA/non-MDR | ND | ND | Negative | Negative |  |
| BIOS-V129 | Rabbit | *S. aureus* | R | S | MSSA/non-MDR | ND | ND | Negative | Negative |  |
| BIOS-V147 | Rabbit | *S. aureus* | I | S | MSSA/non-MDR | ND | ND | Negative | Negative |  |
| BIOS-V151 | Cat | *S. aureus* | I | S | MSSA/MDR | ND | ND | Negative | Negative |  |
| BIOS-V153 | Dog | *S. aureus* | R | R | MRSA/MDR | S80Y/E84G | S84L | Positive | Negative |  |
| BIOS-V155 | Cat | *S. aureus* | R | R | MRSA/non-MDR | ND | ND | Negative | Negative |  |
| BIOS-V156 | Dog | *S. aureus* | I | S | MSSA/non-MDR | ND | ND | Negative | Negative |  |
| BIOS-V157 | Dog | *S. aureus* | I | S | MRSA/non-MDR | ND | ND | Negative | Negative |  |
| BIOS-V158 | Cat | *S. aureus* | I | S | MSSA/non-MDR | ND | ND | Negative | Negative |  |
| BIOS-V159 | Cat | *S. aureus* | R | R | MRSA/non-MDR | ND | ND | Negative | Negative |  |
| BIOS-V160 | Cat | *S. aureus* | R | R | MRSA/non-MDR | ND | ND | Negative | Negative |  |
| BIOS-V161 | Rabbit | *S. aureus* | R | R | MRSA/non-MDR | ND | ND | Negative | Negative |  |
| BIOS-V168 | Cat | *S. aureus* | I | S | MSSA/non-MDR | ND | ND | Negative | Negative |  |
| BIOS-V172 | Dog | *S. aureus* | I | S | MSSA/non-MDR | ND | ND | Negative | Negative |  |
| BIOS-V178 | Cat | *S. aureus* | I | S | MSSA/MDR | WT | ND | Negative | Negative |  |
| BIOS-V183 | Dog | *S. aureus* | R | R | MRSA/non-MDR | ND | ND | Negative | Negative |  |
| BIOS-V186 | Dog | *S. aureus* | R | R | MRSA/non-MDR | ND | ND | Negative | Negative |  |
| BIOS-V187 | Dog | *S. aureus* | R | R | MRSA/non-MDR | ND | ND | Negative | Negative |  |
| BIOS-V200 | Dog | *S. aureus* | R | R | MRSA/non-MDR | S80F | S84L | Positive | Negative |  |
| BIOS-V201 | Dog | *S. aureus* | R | R | MRSA/non-MDR | ND | ND | Negative | Negative |  |
| BIOS-V202 | Cat | *S. aureus* | R | R | MRSA/non-MDR | ND | ND | Negative | Negative |  |
| BIOS-V203 | Dog | *S. aureus* | I | S | MSSA/non-MDR | ND | ND | Negative | Negative |  |
| BIOS-V204 | Rabbit | *S. aureus* | R | R | MRSA/non-MDR | ND | ND | Negative | Negative |  |
| BIOS-V245 | Dog | *S. aureus* | I | S | MSSA/non-MDR | ND | ND | Negative | Negative |  |
| BIOS-V250 | Dog | *S. aureus* | I | S | MSSA/non-MDR | ND | ND | Negative | Negative |  |
| BIOS-V255 | Dog | *S. aureus* | R | R | MRSA/MDR | S80Y/E84G | S84L/E88Q | Positive | Negative^*^ |  |
| BIOS-V257 | Cat | *S. aureus* | I | S | MSSA/non-MDR | ND | ND | Negative | Negative |  |
| BIOS-V258 | Rabbit | *S. aureus* | R | R | MRSA/non-MDR | ND | ND | Negative | Negative |  |
| BIOS-V279 | Cat | *S. aureus* | I | S | MSSA/non-MDR | ND | ND | Negative | Negative |  |
| BIOS-V295 | Unknown | *S. aureus* | I | S | MSSA/non-MDR | ND | ND | Negative | Negative |  |
| BIOS-V296 | Dog | *S. aureus* | R | R | MRSA/non-MDR | S80F | S84L | Positive | Negative^*^ |  |
| BIOS-V300 | Dog | *S. aureus* | R | R | MRSA/MDR | ND | ND | Negative | Negative |  |
| BIOS-V24 | Dog | *S. epidermidis* | S | S | MRSE/MDR | ND | ND | Negative | Negative | ^[9]^ |
| BIOS-V100 | Dog | *S. epidermidis* | R | R | MRSE/MDR | ND | ND | Negative | Negative | ^[8]^ |
| BIOS-V216 | Dog | *S. epidermidis* | R | R | MSSE/MDR | ND | ND | Negative | Negative |  |
| BIOS-V282 | Dog | *S. epidermidis* | S | S | MRSE/MDR | ND | ND | Positive | Positive |  |

ND: Not determined, WT: Wild-type, S: Susceptible, R: Resistant, I: Intermediate, MRSA: Methicillin-resistant Staphylococcus aureus, MRSE: Methicillin-resistant Staphylococcus epidermidis, MDR: Multi-drug resistant, EtBrCW: Ethidium Bromide (EtBr)-agar Cartwheel method. The term “Positive” indicates that the diagnostic method used to detect efflux identified strong efflux activity. For the EtBrCW method, isolates were considered positive if they exhibited fluorescence at the highest EtBr concentration tested (> 2.5 mg/L). Isolates marked with (*) displayed fluorescence at intermediate EtBr concentrations (1–2 mg/L). The term “Negative” signifies that the diagnostic method used to detect efflux identified basal/low efflux activity.

1. **In-vitro cytotoxicity test and Micronucleus assay on HaCaT keratinocytes cells**

**Method:** The in-vitro cytotoxicity test assessed cell viability based on Neutral Red uptake, a dye that accumulates in lysosomes and decreases in response to membrane damage. HaCaT keratinocytes were cultured in DMEM with 10% calf serum and incubated at 37°C, 5% CO₂. Cells were seeded in 96-well plates, treated with eight concentrations of the **5a4**, and incubated for 24 hours. After washing, cells were exposed to Neutral Red medium for 3 hours, followed by washing and destaining. Optical density (OD_540_) was measured using a fluorescence reader, and IC_50_ values were calculated using Phototox Version 2.0, comparing treated wells to untreated controls.^[10]^

The micronucleus assay is a mutagenicity assay which is based on the detection of micronuclei (MNC) in the cytoplasm of interphase cells. Briefly, Human keratinocytes (HaCaT, <50 passages) were cultured in DMEM with 1 mM glutamine and 10% inactivated calf serum. Cells (50,000/well) were plated, incubated overnight, and treated with five **5a4** concentrations for 3 h. A solvent control assessed spontaneous micronuclei levels, and mitomycin C (0.05 µg/mL) served as a positive control. Post-exposure, cells were rinsed and incubated with cytochalasin B (3 µg/mL) for 48 h to block cytokinesis. Fixed, Giemsa-stained slides were examined at 1000x magnification. Cytotoxicity was measured by the Proliferative Index (PI), calculated from mononucleated (M1), binucleated (M2), and trinucleated (M3) cells:

**PI = (M1 + 2x M2 + 3x M3) / 500**.

Micronucleated cell rates were assessed in conditions reducing PI by <50%. Micronuclei were counted in 2000 binucleated cells, with statistical differences determined via the χ^2^ test. A positive result was defined by a dose-dependent increase in micronucleated cells, with at least one concentration inducing a significant rise compared to the control.^[11]^

**Table S2: Results of cytotoxicity of 5a4 on HaCaT keratinocytes cells using the Neutral Red uptake method**

| **Samples** | **Conc.** | **Optical Densities**  **(n=3)** | | | **Cell viability (% as compared to the negative control)** | | |
| --- | --- | --- | --- | --- | --- | --- | --- |
|  | **µg/ml** |  |  |  |  |  |  |
| **Blank** | **-** | 0.05 | 0.05 | 0.05 | - | - | - |
| **Negative control** | **-** | 0.678 | 0.665 | 0.657 | 100 | 100 | 100 |
| **5a4** | 0.00025 | 0.67 | 0.666 | 0.648 | 98.73 | 100.16 | 98.52 |
|  | 0.0005 | 0.671 | 0.658 | 0.633 | 98.89 | 98.86 | 96.05 |
|  | 0.001 | 0.598 | 0.584 | 0.579 | 87.26 | 86.83 | 87.15 |
|  | 0.025 | 0.552 | 0.548 | 0.597 | 79.95 | 80.96 | 90.16 |
|  | 0.05 | 0.468 | 0.492 | 0.458 | 66.56 | 71.87 | 67.22 |
|  | 0.1 | 0.413 | 0.425 | 0.426 | 57.8 | 60.91 | 61.94 |
|  | 0.25 | 0.323 | 0.319 | 0.325 | 43.44 | 43.67 | 45.37 |
|  | 0.5 | 0.284 | 0.274 | 0.289 | 37.26 | 36.42 | 39.37 |
|  | 1 | 0.123 | 0.101 | 0.134 | 11.62 | 8.29 | 13.84 |
|  | 2.5 | 0.095 | 0.1 | 0.098 | 7.17 | 8.13 | 7.91 |

**Table S2: Genotoxic effect of 5a4 on HaCaT keratinocytes cells using micronucleus assay**

| **Conc.**  **µg/ml** | | **Assay performed without S9 mix** | | | **Assay performed with UVA/visible Irradiation** | | |
| --- | --- | --- | --- | --- | --- | --- | --- |
|  |  | **% PI** | **MNC** | **P** | **% PI** | **MNC** | **P** |
| **Negative control** | | 100 | 9 .5 ± 0.7 | - | 100 | 9.5 ± 0.7 | - |
| **Positive control^*^** | | 98.3 | 33.5 ± 0.7 | <0.001 | 99.6 | 35.5 ± 0.7 | <0.001 |
| **5a4** | **0.025** | 99.8 | 9 ± 1.4 | NS | 99.2 | 11 ± 1.4 | NS |
|  | **0.05** | 88.2 | 8.5 ± 0.7 | NS | 94.6 | 9 ± 2.1 | NS |
|  | **0.1** | 78.5 | 11 ± 1.4 | NS | 92.1 | 9.5 ± 0.7 | NS |
|  | **0.25** | 56.1 | 10 ± 1.4 | NS | 89.2 | 11.5 ± 2.1 | NS |
|  | **0.5** | **TOXIC** | | | | | |

Note: MNC refers to micronucleated cells per 1,000. % PI represents the Proliferative Index. NS indicates non-significant results. *Mitomycin C (0.05 µg/mL) was used as the positive control. P indicates the probability of significance, comparing the negative control and tested dosages using the Chi-squared test.

**Results:** The Neutral Red uptake assay (**Table S2**) showed a concentration-dependent decline in HaCaT cell viability. At the lowest test concentration (0.00025 µg/mL), the cell viability remained above 96% compared to the negative control, indicating negligible cytotoxicity. A significant reduction in viability (<50%) was observed from 0.25 µg/mL onward, with the IC_50_ occurring between 0.1 and 0.25 µg/mL. At the highest concentration (2.5 µg/mL), viability dropped drastically to approximately 7.91%, confirming strong cytotoxicity.

The proliferative index (PI) decreased with increasing concentrations of the test compound, suggesting inhibition of cell division. Without metabolic activation (S9 mix), the MNC frequency remained statistically insignificant (NS) across all tested concentrations, indicating no clear genotoxicity. Under UVA/visible light irradiation, a slight increase in MNC frequency was observed at 0.5 µg/mL (11.5 ± 2.1 per 1000 cells), but this increase was not statistically significant (NS). The positive control induced a significant rise in MNC frequency (P < 0.001), validating assay reliability. The test compound exhibited toxicity at 0.5 µg/mL, preventing further analysis (**Table S3**).

**Correlation Analysis:** A clear dose-dependent cytotoxicity was observed, with a steep decline in cell viability at higher concentrations (>0.1 µg/mL). Despite marked cytotoxicity, the micronucleus assay did not indicate a dose-dependent mutagenic effect. The lack of significant micronucleus formation suggested that the test compound predominantly exhibited cytotoxic effects rather than direct genotoxicity. Under UVA/visible irradiation, a slight increase in MNC was observed, but it was within the NS range, implying that phototoxicity did not significantly enhance genotoxic potential.

1. **Safety Handling Recommendations**

- **Skin Contact Precautions:** Strong cytotoxic effects in HaCaT cells suggest potential skin toxicity. Proper personal protective equipment (PPE), including gloves, lab coats, and safety goggles, should be used to prevent dermal exposure.
- **Handling Under Light Conditions:** Since slight phototoxic effects were noted under UVA/visible light irradiation, avoid direct exposure to strong light sources during handling.
- **General Laboratory Precautions:** In case of accidental contact, immediately rinse affected areas with water and seek medical advice if necessary. Proper waste disposal and decontamination protocols should be followed to prevent unintended exposure.

1. **Reference**

[1] Z. Chen, S. Pascal, M. Daurat, L. Lichon, C. Nguyen, A. Godefroy, D. Durand, L. M. A. Ali, N. Bettache, M. Gary-Bobo, et al., *ACS Appl. Mater. Interfaces* **2021**, *13*, 30337.

[2] G. W. Kaatz, V. V. Moudgal, S. M. Seo, J. E. Kristiansen, *Antimicrob. Agents Chemother.* **2003**, *47*, DOI 10.1128/AAC.47.2.719-726.2003.

[3] G. W. Kaatz, F. McAleese, S. M. Seo, *Antimicrob. Agents Chemother.* **2005**, *49*, 1857.

[4] I. Couto, S. S. Costa, M. Viveiros, M. Martins, L. Amaral, *J. Antimicrob. Chemother.* **2008**, *62*, DOI 10.1093/jac/dkn217.

[5] S. S. Costa, M. Viveiros, C. Pomba, I. Couto, *J. Antimicrob. Chemother.* **2018**, *73*, DOI 10.1093/jac/dkx400.

[6] S. Costa, C. Falcão, M. Viveiros, D. MacHado, M. Martins, J. Melo-Cristino, L. Amaral, I. Couto, *BMC Microbiol.* **2011**, *11*, DOI 10.1186/1471-2180-11-241.

[7] C. Ferreira, S. S. Costa, M. Serrano, K. Oliveira, G. Trigueiro, C. Pomba, I. Couto, *Antibiotics* **2021**, *10*, DOI 10.3390/antibiotics10040345.

[8] S. S. Costa, R. Ribeiro, M. Serrano, K. Oliveira, C. Ferreira, M. Leal, C. Pomba, I. Couto, *Antibiotics* **2022**, *11*, DOI 10.3390/antibiotics11050599.

[9] I. C. SS Costa, Carolina Ferreira, C Morais, P. Abrantes, Constança Pomba, in *Microbiotec’21 Congr.*, **2021**, pp. 7823–7830.

[10] M. Blanchet, D. Borselli, A. Rodallec, F. Peiretti, N. Vidal, J. M. Bolla, C. Digiorgio, K. R. Morrison, W. M. Wuest, J. M. Brunel, *ChemMedChem* **2018**, *13*, DOI 10.1002/cmdc.201800073.

[11] C. Botta, C. Di Giorgio, A. S. Sabatier, M. De Méo, *Environ. Toxicol.* **2009**, *24*, DOI 10.1002/tox.20455.
